# Ferrocenyl-Substituted Triazatruxenes: Synthesis, Electronic Properties, and the Impact of Ferrocenyl Residues on Directional On-Surface Switching on Ag(111)

**DOI:** 10.1021/acs.inorgchem.3c03009

**Published:** 2023-09-21

**Authors:** Lars Vogelsang, Tobias Birk, Fabian Paschke, Anja Bauer, Vivien Enenkel, Lukas M. Holz, Mikhail Fonin, Rainer F. Winter

**Affiliations:** † Fachbereich Chemie, Universität Konstanz, 78467 Konstanz, Germany; ‡ Fachbereich Physik, Universität Konstanz, 78467 Konstanz, Germany

## Abstract

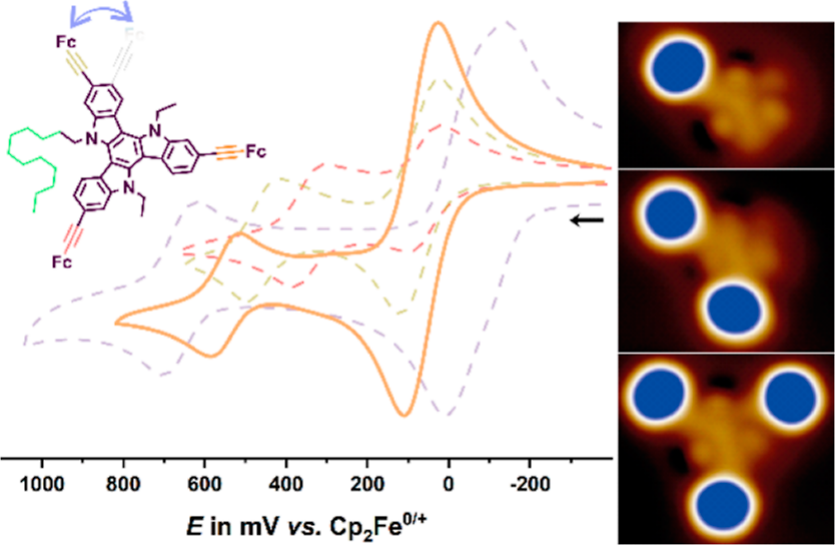

We report on seven new ferrocenyl-(**1**, **3**)- and ferrocenylethynyl-modified *N*,*N*′,*N*″-triethyltriazatruxenes (^**Et**^**TAT**s) **4**–**7** as well as the dodecyl counterpart **2** of compound **1** and their use as molecular switching units when deposited on a Ag(111) surface. Such functional units may constitute a new approach to molecule-based high-density information storage and processing. Besides the five compounds **1**–**3**, **6**, and **7**, where the 3-fold rotational symmetry of the triazatruxene (TAT) template is preserved, we also included 2-ethynylferrocenyl-TAT **4** and 2,2′-di(ethynylferrocenyl)-TAT **5**, whose mono- and disubstitution patterns break the 3-fold symmetry of the TAT core. Voltammetric studies indicate that the ferrocenyl residues of compounds **1**–**7** oxidize prior to the oxidation of the TAT core. We have noted strong electrostatic effects on TAT oxidation in the 2,2′,2″-triferrocenyl-TAT derivatives **1** and **2** and the 3,3′,3″-isomer **3**. The oxidized complexes feature multiple electronic excitations in the near-infrared and the visible spectra, which are assigned to d_δ/δ^*^_ transitions of the ferrocenium (Fc^+^) moieties, as well as TAT → Fc^+^ charge-transfer transitions. The latter are augmented by intervalence charge-transfer contributions Fc → Fc^+^ in mixed-valent states, where only a part of the available ferrocenyl residues is oxidized. ^**Et**^**TAT** was previously identified as a directional three-level switching unit when deposited on Ag(111) and constitutes a trinary-digit unit for on-surface information storage. The symmetrically trisubstituted compound **6** retains this property, albeit at somewhat reduced switching rates due to the additional interaction between the ferrocenyl residues and the Ag surface. In particular, the high directionality at low bias and the inversion of the preferred sense of the on-surface rocking motion with either a clockwise or counterclockwise switching sense, depending on the identity of the surface enantiomer, are preserved. Unsymmetrical substitution in mono- and diferrocenylated **4** and **5** alters the underlying ratchet potential in a manner such that a two-state switching between the two degenerate surface conformations of **4** or a pronounced suppression of switching (**5**) is observed.

## Abstract 2

Seven new triazatruxenes (TATs) with one to three directly attached or ethynyl-linked ferrocenyl donors are reported. The electronic absorptions in their oxidized states are assigned as ferrocenium-localized d_δ_ and TAT → Fc^+^ charge-transfer excitations, augmented by Fc → Fc^+^ intervalence charge-transfer (IVCT) or the IVCT band of the TAT^+^ chromophore. When deposited on Ag(111), the trisubstituted congener retains the directional three-level switching previously observed for the pristine TAT at slightly reduced switching rates.

## Introduction

Ferrocene,^1,2^ and acyclic as well as polycyclic aryl-substituted amines (PAAs) constitute paradigmatic (metal-)organic redox systems. Their hallmarks are their synthetic versatility and stability in both the reduced and the oxidized states.^3–18^ Well-known representatives of the redox-active triarylamines are tris(4-bromophenyl)aminium hexachloroantimonate (“magic blue”) and its more robust “blues cousin”.^19^ Both are widely used, powerful oxidants for preparative or in situ redox reactions.^20^ Furthermore, aryl-substituted and polycyclic triarylamines are frequently employed as dopant-free, cost-effective hole-conducting materials or as one-component hole-conductor and emitter materials for (organic) solar cells or organic light-emitting diodes (OLEDs).^6–8,21–28^ The planarized PAAs constitute an increasingly important subfamily of this compound class. The rigidity of their molecular scaffold can lead to a superior π-conjugation when compared with their propeller-shaped, open congeners. Therefore, they often have delocalized frontier molecular orbitals (MOs), which contribute to the extraordinary stability of their corresponding radical cations.^29^

**Figure 1:**
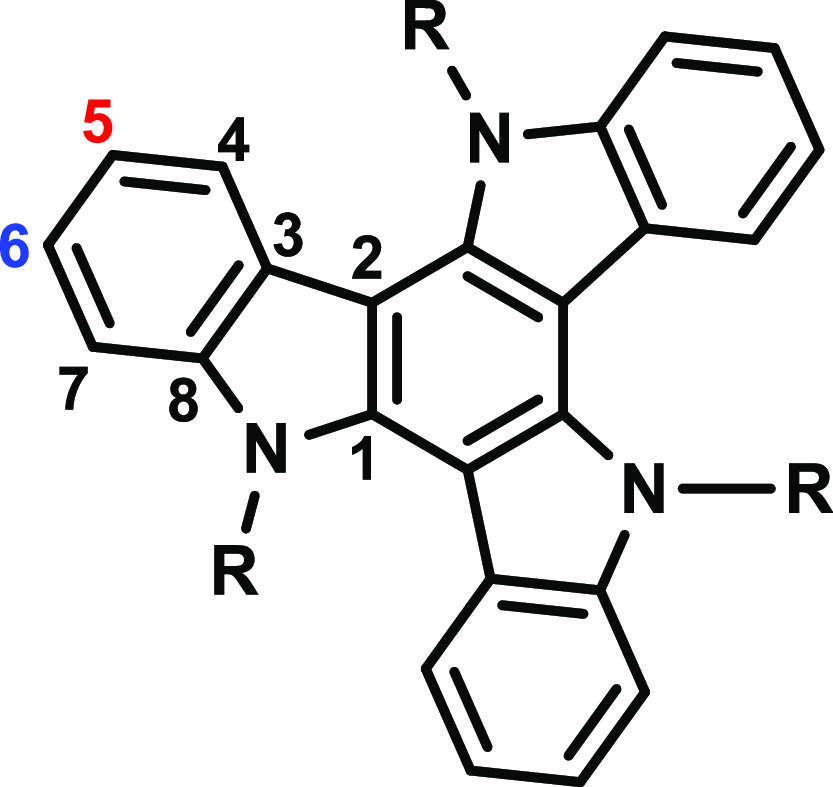
Structure of the TAT template with the numbering of the carbon atoms. The positions marked in red and blue are usually named the 2-/3-positions in terms of the substitution of the TAT core. This numbering is used hereupon.

The pivotal representatives of this compound class are triazatruxenes [(TATs) Figure 1]. TATs possess a planar, π-extended, and C_3_ symmetric skeleton comprising three indoles, which are fused to a common, central benzene ring. *N*-alkylated TATs (R = alkyl) undergo two consecutive one-electron oxidations at half-wave potentials of ca. 300 and 900 mV against the ferrocene/ferrocenium standard (Cp_2_Fe^0/+^). The first oxidation is chemically and electrochemically reversible, whereas the chemical reversibility of the second step depends on the nature of the substituent R and the supporting electrolyte.^19^ Radical cations and symmetrically trisubstituted TATs are mixed-valent systems of Class III according to the Robin and Day classification scheme with complete charge-delocalization over the entire π-conjugated scaffold.^30^ Furthermore, TATs can be readily deposited on substrates by vapor deposition or solution film processing techniques.^29,31^ This makes them potent constituents of hole-conducting materials in solar cells or emitters for OLEDs.^31–36^ Moreover, appropriately functionalized TATs form columnar mesophases with electrical conductivity along the stacking axis on doping.^37–41^

We have recently shown that *N*,*N*′,*N*″-triethyltriazatruxene (^**Et**^**TAT**, Figure 1, R = Et) serves as a programmable molecular switch when deposited on Ag(111).^42^ Binding to the substrate results in two degenerate, enantiomeric configurations, *R* (clockwise rotation for the symmetry equivalent transformations) and *S* (counterclockwise rotation for symmetry equivalent transformations). Isolated ^**Et**^**TAT** molecules on Ag(111) adsorb with their central benzene ring atop an Ag atom and the pyrrole N atoms either above the face-centered cubic (atop-*fcc*) or hexagonally close packed (atop-*hcp*) hollow sites. Owing to a slight mismatch between the positions of the surface Ag and the TAT N atoms, the molecules adopt a skewed adsorption geometry with two shorter and one longer Ag–N distances. This results in three distinguishable states on the surface. The reversible and directional switching in either a clockwise (*R* surface enantiomer) or counterclockwise (*S* enantiomer) direction between these three states was achieved by placing the tip of a scanning tunneling microscope (STM) in a lateral position above a TAT molecule and adjusting the tunneling current and bias voltage. The switching relies on a rocking motion of the ^**Et**^**TAT** molecules in a ratchet-like potential yet not on a rotational motion.^42^ Hence, the TAT molecules on Ag(111) constitute three-state, trinary-digit (trit) units for on-surface information storage. This adds to other current proof-of-principle realizations of molecular on-surface switching that rely on the different charge states of molecules,^43,44^ intramolecular atom (usually proton) transfer,^45^ reversible conformational changes,^46–48^ rotational motions of molecules,^49,50^ or the reversible formation and breaking of chemical bonds.^51,52^ Individual TAT molecules or small ensembles thereof may hence serve as information storage units for high-density logic and memory devices with the potential to outperform the conventional electronic components of the silicon-based semiconductor industries.

To achieve still higher levels of control and addressability of the TAT molecules, we envision decorating them with active functional units. The ideal candidates for such an endeavor would be nickelocene-modified TATs. Nickelocenes show magnetic anisotropy, resulting from their degenerate *e*_2g_*^2^ electronic ground state.^53^ However, it is unclear whether and to what extent the attachment of metallocenyl units will modify the adsorption behavior and on-surface geometries of the TAT molecules on Ag(111) and whether the so-modified TATs may still undergo on-surface switching. As the first step toward the realization of our goal, we set out to prepare and investigate mono-, di-, and triferrocenyl derivatives of ^**Et**^**TAT** and study their switching behavior on Ag(111).

Despite the importance and advantageous properties of both ferrocenes and PAAs, only a few examples of coupled redox systems that combine both types of constituents were reported.^54,55^ The direct connection between the five-membered Cp and the six-membered phenyl rings of the TAT framework allows for their rather coplanar arrangement, similar to what is seen for aryl-substituted ferrocenes.^55–58^ Small torsions at these linkages are highly advantageous for on-surface deposition and STM studies.^59^ From a chemist’s viewpoint, Fc-appended TATs are also of interest with regard to their rich redox chemistry. The direct coupling of 1–3 ferrocenyl units to a common TAT template provides multistate redox systems with three to five potentially accessible redox states. Several of them are mixed-valent in nature and are hence expected to exhibit intramolecular intervalence and intercomponent charge-transfer excitations. This dimension can be studied by voltammetric and spectroscopic investigations of the molecules in their various redox states. The results of our forays into these topics are discussed in the following.

## Discussion

### Synthesis of Ferrocenyl-Modified TATs

**Figure 2:**
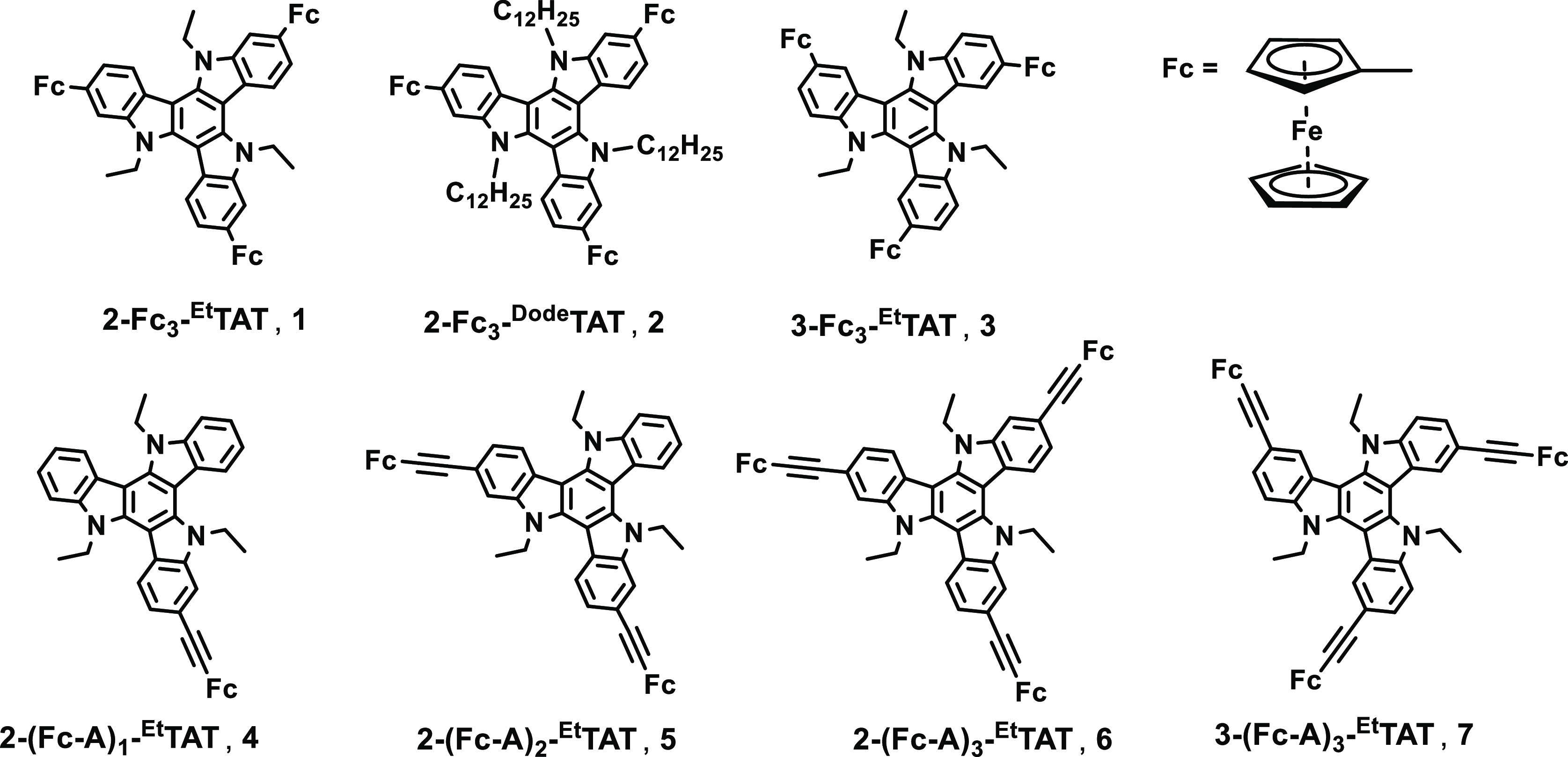
Chemical structures of the ferrocenyl-TAT conjugates of this study.

In pursuit of the goals of our study, we prepared the seven different ferrocenyl-substituted TATs (Fc-TATs) shown in Figure 2. The individual representatives belong to two different families with either direct C–C linkages between a ferrocenyl cyclopentadienyl ring and the TAT scaffold (compounds **1**–**3**) or a connection over ethynyl spacers (compounds **4**–**7**). For both the families, the positional 2,2′,2″-(abbreviated as 2-) and 3,3′,3″-(abbreviated as 3-) isomers of the trisubstituted congeners were realized (compounds **1** and **3**, **6**, and **7**. They differ with respect to the locations of the ferrocenyl residues at the indolic benzene rings. Additional variations include the substitution of the *N*-ethyl by *N*-dodecyl substituents (compounds **1** and **2**) and, for the ethynyl-linked 2-isomers, the number of the attached ferrocenyl nuclei (one to three, compounds **4**–**6**). The synthetic details as well as the proton (^1^H) and carbon-13 nuclear magnetic resonance (^13^C NMR) and the mass spectra are provided in the Supporting Information (see Figures S1–S33).

**Scheme 1:**
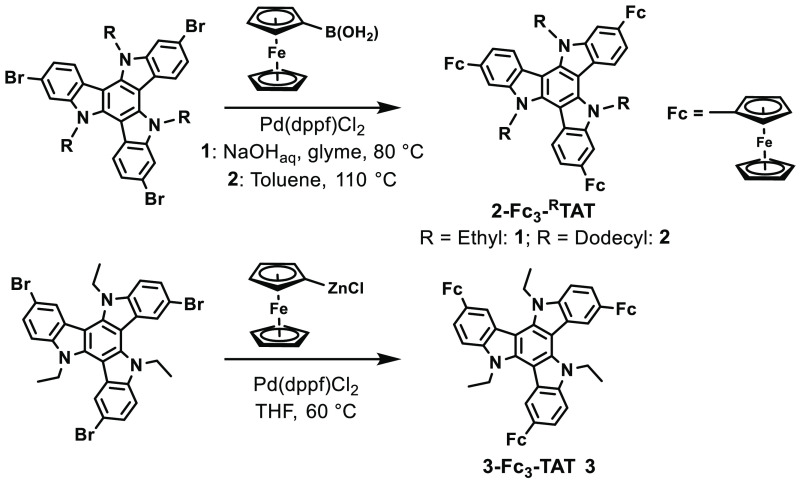
Synthesis of the Triferrocenyl-TAT Conjugates **1**–**3** with Direct C–C Linkages between the Cyclopentadienyl Rings and the TAT Core

The key synthesis step is the C–C-cross-coupling between an appropriate ferrocenylating agent and the corresponding TAT building block. The Suzuki- or Negishi-type cross-couplings provided the best results for preparing Fc-TATs with direct Fc-TAT linkages (Scheme 1).

Thus, when literature-known 2,2′,2″-tribromo-^Et^TAT (**2-Br**_**3**_**-**^**Et**^**TAT**)^10^ was coupled with excess ferrocenyl boronic acid^13^ (6 equiv), trisubstituted **2-Fc**_**3**_**-**^**Et**^**TAT** (**1**) was obtained in an isolated yield of 10% after purification. Dodecyl-substituted **2-Fc**_**3**_**-**^**Dode**^**TAT** (**2**) was prepared in the same manner (54% yield) from the appropriate tribromo-TAT precursor. Compound **2** offers significantly enhanced solubility in unpolar solvents, including *n*-pentane, which may be advantageous for further processing. Moreover, the TATs with long *N*-alkyl substituents are known for their superior film-forming and -processing properties and liquid crystalline behavior.^13,38,60,61^ For synthesizing **3-Fc**_**3**_**-**^**Et**^**TAT** (**3**), the Negishi cross-coupling reaction of **3-Br**_**3**_**-**^**Et**^**TAT** with in situ prepared ferrocenyl zinc chloride gave superior results over that from the Suzuki protocol. The synthesis of the isomeric, ethynyl-linked trisubstituted Fc-TATs **6** and **7** employed standard protocols for the Sonogashira cross-coupling reactions using ethynylferrocene and the respective tribromo-TAT as the precursors. The synthetic details are provided in the Supporting Information.

**Scheme 2:**
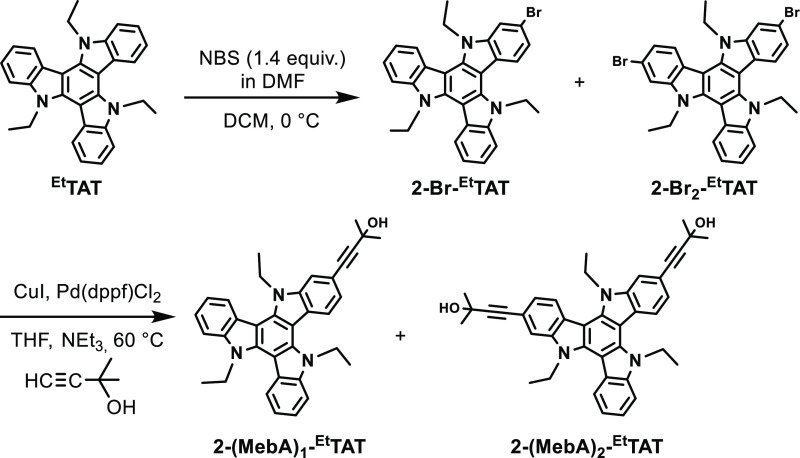
Synthesis of the Mebynol-Protected Precursors of Alkynes **2-A**_**1**_**-**^**Et**^**TAT** and **2-A**_**2**_**-**^**Et**^**TAT**

A further point of interest was to study whether and how an increasing number of ferrocenyl substituents affect the redox, optical, and on-surface switching properties of the ethynylferrocenyl-TATs. We therefore also synthesized the 2-mono- and the 2,2′-di(ferrocenylethynyl) derivatives **4** and **5**. Their synthesis involves desymmetrization of the TAT core. The 2- isomers of the monobrominated or monoethynylated TATs with *N*-methyl or longer *N*-alkyl substituents were reported in the literature,^62–66^ but not for ^**Et**^**TAT**. When applied to ^**Et**^**TAT**, the reported methods yielded inseparable mixtures of the 2-ethynyl- and the 2,2′-di(ethynyl)-substituted TATs as a result of the unselective bromination, even in the presence of less than 1 equiv of the brominating agent. We therefore devised a new synthetic strategy that allows for an efficient separation of the non-, mono-, and diethynylated TATs. As shown in Scheme 2, our improved procedure employs the 3,3-dimethylprop-1-yn-3-ol (mebynol) functionality for that purpose. Thus, the mixture of non-, mono-, and di(brominated) TATs obtained from the bromination of ^**Et**^**TAT** with 1.4 equiv of *N*-bromosuccinimide (NBS) was subjected to Sonogashira coupling with mebynol. Owing to their different polarities, the resulting mixture of unreacted ^**Et**^**TAT** and the 2-mono- and 2,2′-disubstituted derivatives are readily separated on a preparative column. After purification, the mebynol-protecting groups are easily removed by thermal treatment with KOH in toluene/MeOH/H_2_O to provide the corresponding terminal alkynes **2-A**_**1**_**-**^**Et**^**TAT** and **2-A**_**2**_**-**^**Et**^**TAT** (see Chemical Synthesis and Characterization in the Supporting Information).

Mebynol protection thus grants reliable and convenient access to the pure mono- and diethynyl-substituted TATs and reproducibly provides **2-A**_**1**_**-**^**Et**^**TAT** in 55% yield and dialkyne **2-A**_**2**_**-**^**Et**^**TAT** in a yield of 30%. Starting from the ethynyl-TAT precursors and iodoferrocene, the target 2-ethynylferrocenyl- and 2,2′-di(ethynylferrocenyl)-substituted TATs **4** and **5** were obtained in yields of 29% and 34%, respectively, under the conditions of the Negishi coupling reactions (see the Supporting Information for details).

In the following, we discuss the properties of the different Fc-TATs. We will use the triferrocenylated compound **1** as the representative example and then highlight the differences in compounds **2**–**7**.

### Redox Properties

The alkylated TATs are known to undergo two consecutive one-electron oxidations: first to the radical cation and then the dication with a half-wave potential separation Δ*E*_1/2_ of about 600 mV. The first oxidation usually fulfills the criterion of a chemically and electrochemically reversible Nernstian process. The second oxidation may however suffer from chemical irreversibility subject to the solvent and the counterion or, when electron-withdrawing substituents are present, may even be shifted to outside the accessible potential range.^19,60,61,65,67–73^ Attaching the redox-active substituents enhances the redox properties of the TATs even further.^60,67,73^ This is also the case for the present ferrocenyl-modified TATs. We studied their redox properties and those of some of their crucial precursors in the CH_2_Cl_2_/NBu_4_^+^ [B{C_6_H_3_(CF_3_)_2_–3,5}_4_]^−^ (0.04 M) electrolyte. The very weakly ion-pairing [B{C_6_H_3_(CF_3_)_2_–3,5}_4_]^−^ anion was chosen in order to render even the highly charged, oxidized forms soluble and to provide a non-nucleophilic environment.^74,75^ The relevant data are listed in Table 1.

**Table 1:** CV Data of the Ferrocenyl-Modified TATs of This Study Recorded in CH_2_Cl_2_/NBu_4_^+^ [B{C_6_H_3_(CF_3_)_2_–3,5}_4_]^−^ (0.04 M) at r. t. Potentials Are Provided Relative to the Cp_2_Fe/Cp_2_Fe^+^ Standard

	*E*_1/2_^Fc/Fc+^	*E*_1/2_^TAT/TAT+^
^**Et**^**TAT**		298
**1**	–133, −60, 0^a^	667
**2**	–88, −13, +41^a^	710
**3**	–35^b^	758
**4**	59	354
**5**	61	455
**6**	69	554
**7**	72	595
**2-A**_**1**_**-**^**Et**^**TAT**		331
**2-A**_**2**_**-**^**Et**^**TAT**		335
**2-A**_**3**_**-**^**Et**^**TAT**		385

a Obtained by digital simulation of the experimental voltammograms.

b The average value.

**Figure 3:**
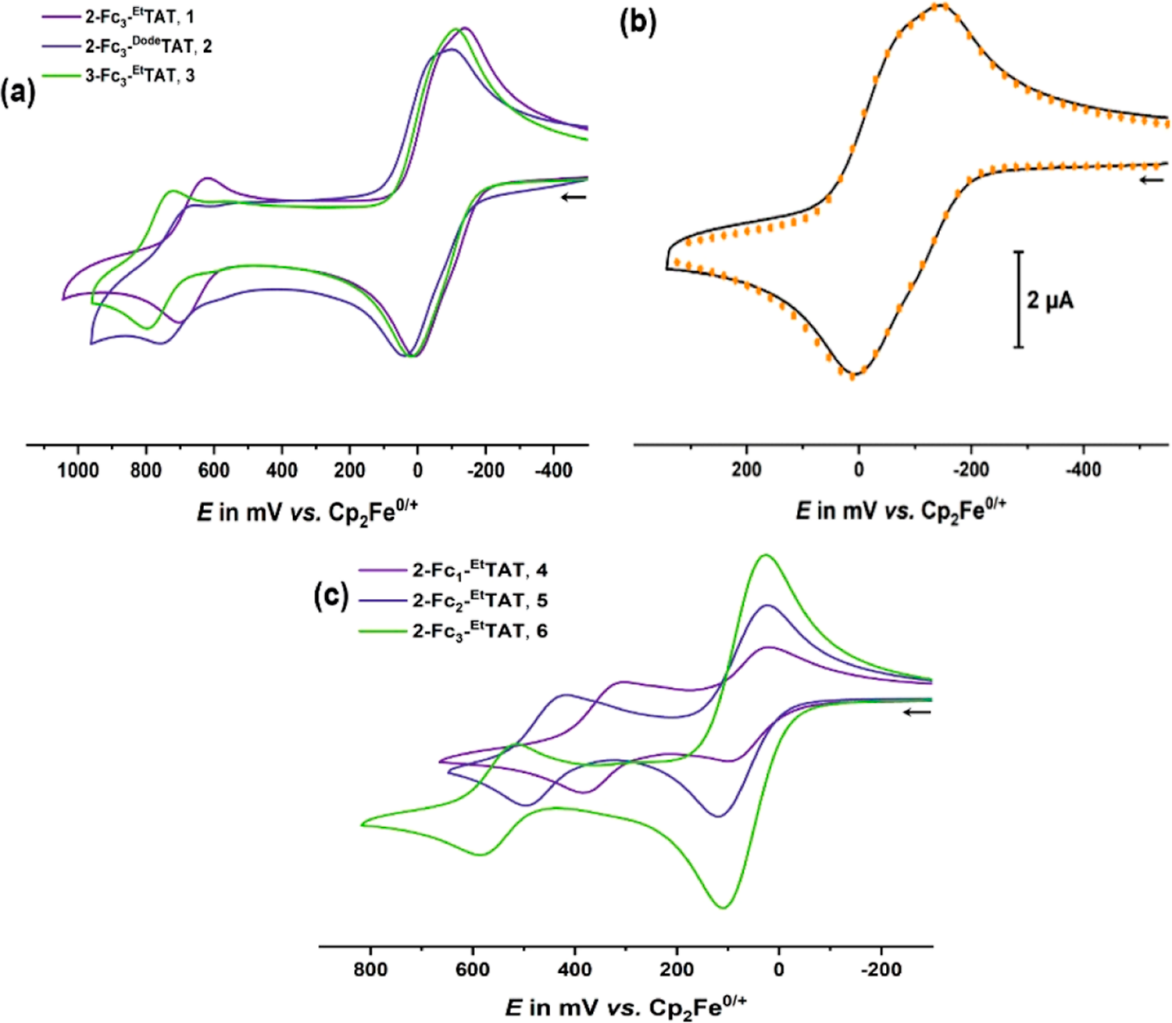
(a) Cyclic voltammograms of triferrocenyl-TATs **1**–**3** in CH_2_Cl_2_/NBu_4_^+^ [B{C_6_H_3_(CF_3_)_2_–3,5}_4_]^−^ (0.04 M) at room temperature (r.t.) and *v* = 100 mV/s. (b) Overlay of the experimental (black line) and digitally simulated (red dots) cyclic voltammograms of **1** at *v* = 800 mV/s. (c) Cyclic voltammograms of ethynylferrocenyl-substituted TATs **4**–**6** in CH_2_Cl_2_/NBu_4_^+^ [B{C_6_H_3_(CF_3_)_2_–3,5}_4_]^−^ (0.04 M) at r. t. and *v* = 100 mV/s.

Cyclic voltammograms of triferrocenylated **2-Fc**_**3**_**-**^**Et**^**TAT** (**1**) and **2-Fc**_**3**_**-**^**Dode**^**TAT** (**2**) as well as the 3-isomer **3** are shown in Figure 3a. The redox reactions of the three equivalent ferrocenyl pendants of **1** merge into a composite wave centered at an apparent half-wave potential *E*_1/2_ of −67 mV with small yet discernible splittings between the individual one-electron processes. Digital simulation of the experimental voltammograms provided half-wave potentials of −133 mV (*E*_1/2_^0/+^), −60 mV (*E*_1/2_^+/2+^), and 0 mV (*E*_1/2_^2+/3+^).^76,77^Figure 3b demonstrates the level of agreement between the simulated and experimental data for a scan recorded at a sweep rate of *v* = 800 mV/s. Figure S34 shows a comparison between the experimental (black line) and simulated square wave voltammograms of **1** (broken green line) as obtained from three separate square wave peaks at the half-wave potentials taken from a digital simulation of the cyclic voltammograms. The first one-electron oxidation of the TAT core is observed at *E*_1/2_ = 667 mV. Due to the combined effects of electron withdrawal by the three peripheral ferrocenium moieties and Coulombic repulsion, the TAT^0/+^ wave is shifted anodically by more than 350 mV when compared to that of ^**Et**^**TAT** itself (*E*_1/2_ = 298 mV as measured at *v* = 100 mV/s in CH_2_Cl_2_ (DCM) (0.1 M NBu_4_^+^ PF_6_^–^, see Figure S35; literature value: 380 mV against Cp_2_Fe^0/+^ in CH_3_CN/0.1 M NBu_4_^+^ PF_6_^–^).^19^ The second oxidation of the TAT core could not be observed within the accessible solvent window of the electrolyte.

The cyclic voltammograms of the Fc-TATs **2** and **3**, which also have direct C–C linkages between the Fc residues and the TAT core, are very similar with only minor shifts of half-wave potentials (see Figure 3a, Table 1, and Figures S36 and S37). Replacing the *N*-ethyl by the *N*-dodecyl substituents (compound **2**) causes anodic shifts of ca. 45 mV for all the redox processes but hardly any differences in the half-wave potential splittings. Changing the sites of the ferrocenyl attachment from the 2, 2′,2″- to the 3, 3′,3″-positions, i.e., from meta to para with respect to the indole *N* atoms, induces slight anodic shifts of ca. 30 mV for the ferrocene-based redox steps and a larger one of ca. 90 mV for TAT oxidation. This indicates a stronger interaction between the 3,3′,3″-positions and the TAT core as opposed to the 2,2′, and 2″-positions, which is in line with the previous observations.^78^

The ethynylene spacers act as mildly electron-withdrawing substituents and separate the individual redox centers spatially and electronically from each other. They thereby attenuate the electronic coupling between the ferrocenyl residues and the TAT core and between the ferrocenyl pendants themselves. Hence, the one-electron oxidations of the ferrocenyl residues in tri(ethynylferrocenyl)-TATs **6** and **7** shift anodically by an average of ca. 135 mV (**6** vs **1**) or 105 mV (**7** vs **3**) and fall into a single, overall three-electron wave without any discernible redox splitting (see Figures 3c and S38). The diminished electronic interactions, the larger spatial separation from the ferrocenium residues, and a concomitant decrease of Coulombic repulsion result in the lowering of the redox potential for TAT oxidation by ca. 110 and 160 mV when compared to that of **1** and **3**. By the same token, the influence of the attachment site on the half-wave potentials for the ferrocene oxidations vanishes altogether, while that on TAT oxidation decreases from 91 (**3** vs **1**) to 41 mV (**7** vs **6**).

**Figure 4:**
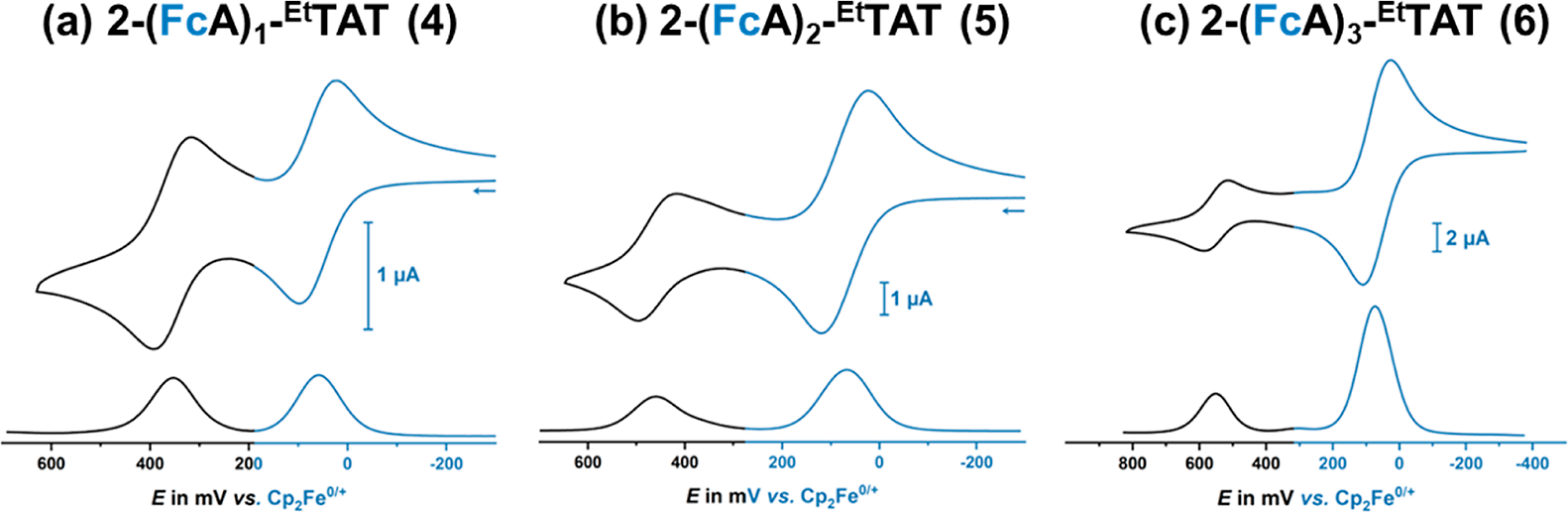
Cyclic (top) and square wave (bottom) voltammograms of (a) 2-ethynylferrocenyl-TAT **4**, (b) 2,2′-di(ferrocenylethynyl)-TAT **5**, and (c) 2,2′,2″-tri(ferrocenylethynyl)-TAT **6** in CH_2_Cl_2_/NBu_4_^+^ [B{C_6_H_3_(CF_3_)_2_–3,5}_4_]^−^ (0.04 M) at r. t. and *v* = 100 mV/s.

Mono- and di(ethynylferrocenyl)-TATs **4** and **5** show similar redox waves but with 1:1 or 2:1 ratios of the peak currents associated with the ferrocene- and the TAT-based redox processes instead of the 3:1 ratio of **6** and **7** (Figure 4a–c). Comparison of the half-wave potentials shows that *E*_1/2_ for the TAT^0/+^ wave increases in a linear fashion by 100 mV per added ethynylferrocenyl (or, rather, ethynylferrocenium) residue (see Figures 3c and S39, as well as the data in Table 1). This implies that substituent effects on TATs are additive, as expected for a planar, fully delocalized, and extended π-system.

### Charge-Transfer Excitations, as Probed by UV/Vis/NIR Spectroelectrochemistry

**Figure 5:**
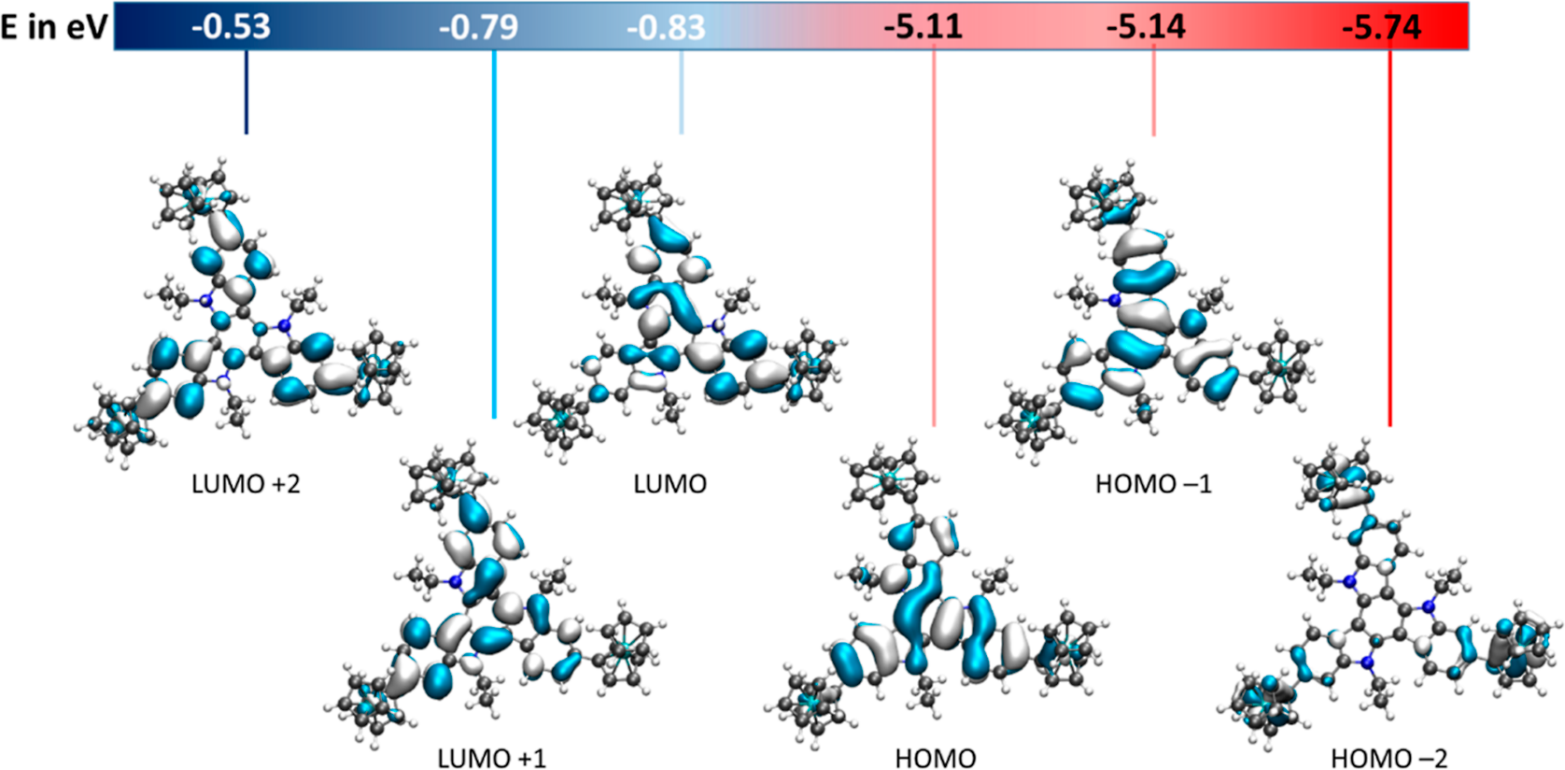
Computed frontier orbitals of **2-Fc**_**3**_**-TAT** (**1**) with the corresponding energies in electron volts (eV) from higher (blue) to lower energies (red).

As imposed by their 3-fold rotational symmetry, the TATs and their symmetrically trisubstituted derivatives have a peculiar electronic structure with nearly degenerate occupied and unoccupied frontier orbitals HOMO to HOMO – 2 (HOMO = highest occupied MO) and LUMO to LUMO + 2 (LUMO = lowest unoccupied MO).^68,79–81^ The frontier MOs are delocalized over all three indolyl rings of the π-conjugated framework. This also holds for triferrocenyl-TAT **1**, as indicated by our quantum chemical calculations based on density functional theory (DFT). Details of the time-dependent (TD-)DFT studies are provided in the Supporting Information. Figure 5 displays the six frontier molecular orbitals (MOs) from HOMO – 2 to LUMO + 2; the plots of additional MOs of relevance to the electronic excitations are provided in Figures S52 and S53.

**Figure 6:**
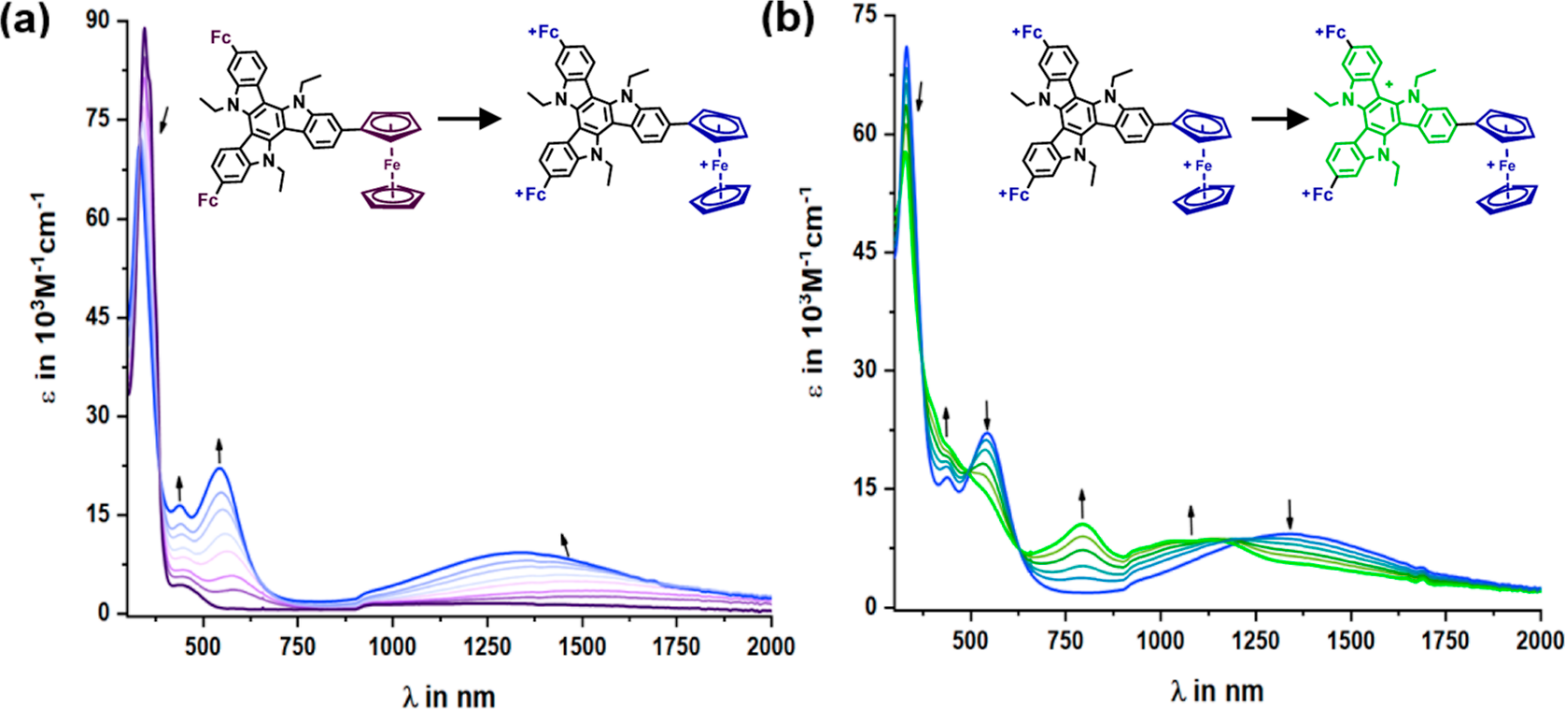
Changes in the UV/vis/NIR spectrum of **2-Fc**_**3**_**-TAT** (**1**) during the (a) first three ferrocenyl-based oxidations and (b) fourth TAT-based oxidation, as recorded during electrolysis in an OTTLE cell in the 0.04 M 1,2-C_2_H_4_Cl_2_/NBu_4_^+^ [B{C_6_H_3_(CF_3_)_2_–3,5}_4_]^−^ electrolyte at r. t. The color coding of the structure formulas reflects the coloring of the lines of the corresponding spectra.

The electronic spectra of the neutral triferrocenyl-TATs **1**–**3** and ethynylferrocenyl-substituted TATs **4**–**7** feature a weak band at ca. 450 nm, which has no equivalent in simple TATs,^68,80,82^ their ethynylated analogues,^63,83^ and other donor-appended TATs (see Figure 6).^84,85^ A similar absorption is however seen in related triferrocenyl truxenes.^86^ Our TD-DFT calculations on compound **1** assign this band to excitations which are accompanied by some transfer of electron density from the Fe atoms of two ferrocenyl donors to the other side arm with the respective phenyl-cyclopentadienide entity acting as the acceptor (see Figure S52). The position of this band is nearly invariant among complexes **1**–**7** (see Table 2). As usual, the UV absorption envelope with a prominent peak at ca. 320 to 340 nm entails several π → π* transitions within the TAT chromophore. Our TD-DFT calculations indicate that the HOMO – 1 → LUMO and the HOMO – 1 → LUMO + 1 excitations have the highest oscillator strengths. The corresponding electron density difference maps (EDDMs) are also included in the Supporting Information (see Figure S52).

**Table 2:** UV/Vis/NIR Spectroscopic Data of the Ferrocenyl-Modified TATs; Wavelength in nm (Extinction Coefficient in L·mol^–1^·cm^–1^)

	neutral	*n* Fc^+^	TAT^+^
**2Fc**_**3**_**-TAT**	450 (4370); 344 (87,300)	1337 (9000); 543 (22,000); 437 (16,100); 331 (69,700)	1489 (4800), 1370 (8400); 1080 (8400), 795 (10,400); 542 (14,400); 444 (19,800); 328 (56,600)
**2Fc3-NDodeTAT**	451 (3000); 344 (85,400)	1415 (4800); 553 (14,600); 442 (9500); 333 (66,400)	1924 (4700); 1367 (9700); 1199 (10,270); 766 (11,200); 539 (21,200); 439 (16,600)
**3Fc3-TAT**	440 (1470); 322 (65,600)	1150 (3100); 502 (11,700); 315 (57,800); 268 (46,700)	1900 (3200); 1188 (5600); 774 (6500); 512 (5200); 392 (10,500)
**2FcA1-TAT**	550 (2400); 363 (37,700)	1483 (3200); 560 (9200); 462 (8000); 363 (30,400)	1484 (3200); 1188 (6300); 1020 (6400); 739 (13,500); 469 (11,800)
**2FcA2-TAT**	455 (3500); 355 (76,300)	1399 (5600); 552 (19,000); 467 (15,000)	1415 (7500); 1193 (11,300); 1020 (11,800); 816 (19,700); 470 (23,600); 425 (29,800)
**2FcA3-TAT**	440 (4450); 355 (110,000)	1196 (5100); 520 (17,900); 445 (19,700); 344 (84,700)	1200 (3800); 808 (2500); 532 (7300); 445 (17,200); 340 (54,600)
**3FcA3-TAT**	442 (3100); 340 (71,800); 323 (74,400)	1093 (4500); 499 (18,200); 401 (20,000); 334 (70,700); 302 (77,100)	1107 (2800); 742 (4300); 572 (7000); 439 (17,000); 335 (31,200)

Stepwise oxidation of the ferrocenyl-modified TATs is expected to give rise to different kinds of electronic excitations: Fc → Fc^+^ IVCT in mixed-valent states, where neutral and oxidized ferrocenyl units coexist (i.e., where only some of the available ferrocenyl residues are oxidized), TAT → Fc^+^ charge transfer (CT), ferrocenium-localized d_δ/δ^*^_ transitions between the nondegenerate Fe-centered orbitals d_*x*_^2^_–*y*_^2^ and d_*xy*_, and π → π* transitions within the TAT chromophore itself. After the final TAT-based oxidation, the bands associated with the TAT^+^ chromophore should also be present. The radical cation of the *N*,*N*′,*N*″-trioctyl-substituted TAT was previously identified as a genuinely delocalized mixed-valent system of Class III,^36^ as indicated by the solvent-independent, vibrationally structured IVCT band.^30^ The same probably applies to the *N*,*N*′,*N*″-triphenyl TAT radical cation, which shows identical hyperfine splittings to the three nitrogen atoms in electron paramagnetic resonance (EPR) spectroscopy.^87^

In order to monitor the oxidation-induced changes in the electronic absorption spectra of the new Fc-TAT dyads, we resorted to the in situ method of UV/vis/NIR spectroelectrochemistry (SEC). In SEC, the spectra are continuously recorded, while the compound of interest is subjected to electrolysis at an appropriate applied working potential inside an optically transparent thin-layer electrolysis (OTTLE) cell. The latter was constructed according to the design of Hartl and co-workers.^88^ In the case of the present di- and triferrocenyl-TATs, the close proximity or coincidence of the individual ferrocene-based oxidations precludes us from obtaining the genuine spectra for any intermediate redox state where only part of the ferrocenyl residues are oxidized. Rather, the spectra recorded while traversing the first overall three-electron process of triferrocenyl-TATs **1**–**3**, **6**, and **7** or the two-electron wave of **5** corresponds to mixtures of species that differ with respect to the number of already oxidized and remaining reduced ferrocenyl residues.

Figure 6 displays the results of our UV/vis/NIR SEC study on **2-Fc**_**3**_**-TAT** (**1**), shown as a representative example. Compilations of the results of SEC experiments on all other Fc-TATs are collected in the Supporting Information. Table 2 summarizes the corresponding spectroscopic data. Successive oxidation of the ferrocenyl pendants gives rise to a continuously intensifying absorption in the NIR spectrum (see Figure 6a). The band maximum shifts gradually to higher energies, from initially about 1600 nm (6250 cm^–1^) to ultimately 1340 nm (7690 cm^–1^). Concomitant with progressing oxidation is the growth of another absorption band at ca. 540 nm. Similar to the NIR envelope, this feature also exhibits a gradual blue shift as more ferrocenyl residues are oxidized, from ca. 584 to 543 nm in **1**^**3+**^. Another band is found at 437 nm. This feature retains its position during the entire oxidation sequence up to the tetracation level. In the near UV, some decrease and blue shift of the prominent π → π* excitations within the TAT chromophore are noted.

**Figure 7:**
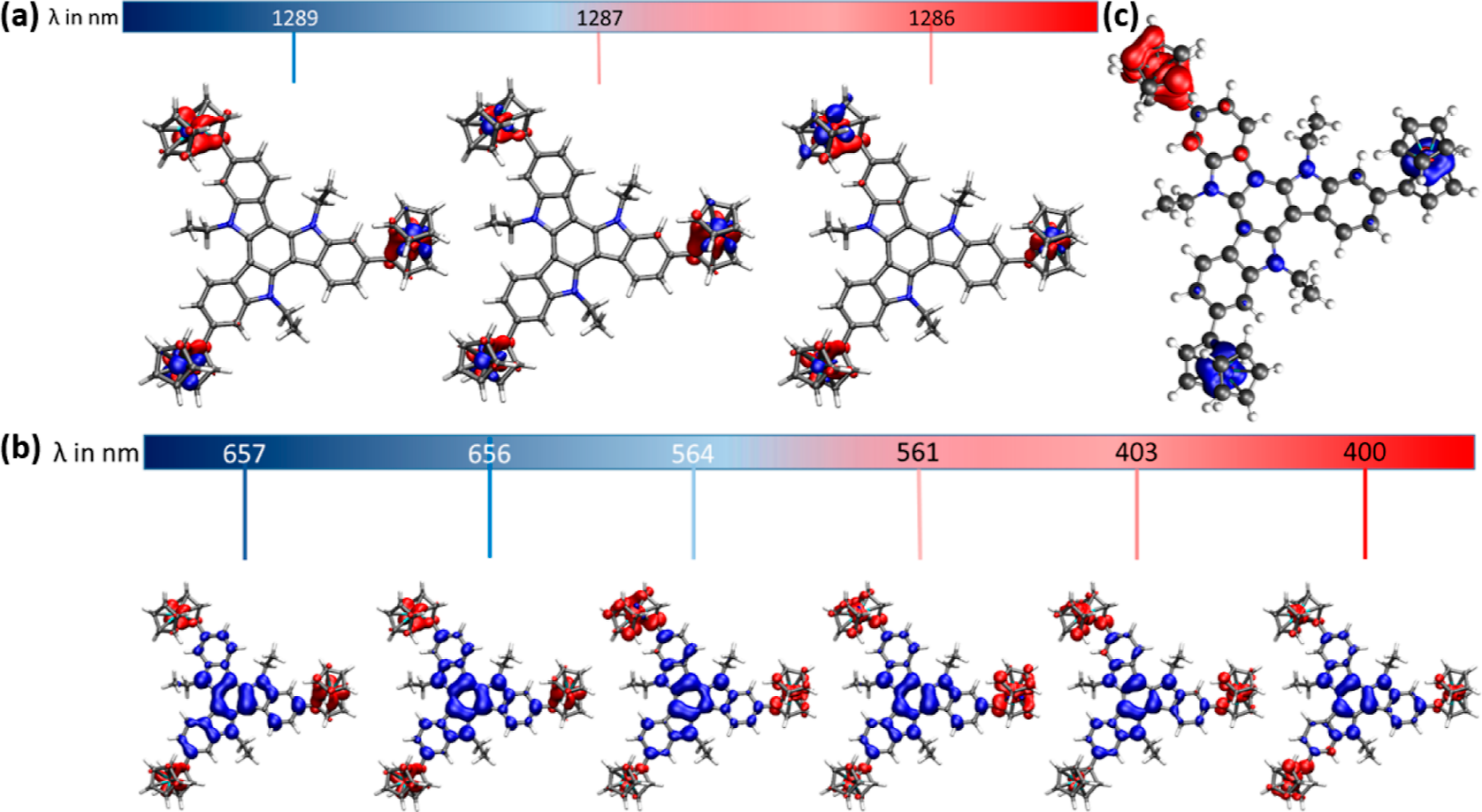
EDDMs for important computed transitions of triferrocenium species **1**^**3+**^, ordered from lower to higher energy (left to right; blue to red color coding). Electron density loss is indicated in red color and electron density gain in blue color. (a) Fe d_δ/δ^*^_ transitions in the NIR region; (b) TAT → Fc^+^ CT transitions in the vis region; and (c) representative IVCT transition in **1**^**+**^ (λ_calc_ = 471 nm).

Our assignment of the electronic excitations under the absorption envelope is aided by TD-DFT calculations on neutral **1**, monooxidized **1**^**+**^, and the triferrocenium-species **1**^**3+**^ and **1**^**4+**^. As indicated by the spin density plots in Figure S57, our calculations agree with the experiments in assigning the first three oxidations to the ferrocenyl pendants, although occupied frontier MOs with dominant ferrocene contributions of **1** appear only starting from HOMO – 2. The absorption in the NIR spectrum that forms during the ferrocene-based oxidations of **1** results from excitations of mainly d_δ/δ^*^_ character, which are confined to the ferrocenium substituents. Figure 7 provides the EDDMs of the three computed transitions that contribute to the NIR envelope. Ferrocene is appended to a π-extended, planar scaffold, and the degeneracy of the δ-orbitals d_*x*_^2^_–*y*_^2^ and d_*xy*_ is lifted as one of these MOs interacts more strongly with the arene π-system. The HOMO of such a ferrocene derivative is represented by an antibonding interaction between the respective δ orbital and a π MO of the attached arene.^89^ In the case of **1** and, by inference, the other triferrocenyl-TATs, the occupied frontier MOs below the HOMO (here, HOMO – 2 to HOMO – 7) have such character and represent different out-of-phase combinations of the Fe d_δ_ orbitals at the individual ferrocenyl residues. These MOs become the β-LUMOs in the first three oxidized states (see Figures S53, S55, and S56 of the Supporting Information). Discrepancies between a ferrocene-centered oxidation and a delocalized HOMO have already been noted for other ferrocenes with a π-extended substituent or another redox-active constituent attached.^90,91^ They are ascribed to the extra stabilization of a ferrocenium ion imposed by a sizable lowering of the Fe d_*z*_^2^ orbital during ferrocene oxidation. We note that the same effect also underlies the inverted energy ordering of the frontier MOs in ferrocene as well as phenylferrocene and their corresponding ferrocenium ions (i.e., d_*x*_^2^_–*y*_^2^, d_*xy*_ < d_*z*_^2^ in ferrocene, but d_*z*_^2^ < d_*x*_^2^_–*y*_^2^, d_*xy*_ in the ferrocenium ion).^92–94^

Our calculations on monooxidized **1**^**+**^ provide no indication of any additional Fc → Fc^+^ IVCT excitation in the NIR. However, such IVCT contributions are noted for the more prominent vis absorption. The spectral envelope of **1**^**3+**^ in the vis region with an absorption onset at ca. 650 nm and the main peak at 543 nm result from several individual excitations, all of which have a TAT → Fc^+^ CT character. Individual excitations differ with respect to the nature of the TAT donor and Fc^+^ acceptor orbitals. Figure 7 provides the EDDMs of the transitions associated with the largest oscillator strengths. In **1**^**+**^, the remaining reduced ferrocenyl entities also contribute, so that these transitions assume a mixed CT/IVCT character (see Figure S53). Our calculations also reproduce the gradual blue-shift of the band envelope on progressing oxidation as bands of such character are computed at wavelengths from 764 to 748 nm and from 646 to 620 nm in **1**^**+**^ but at 650 to 610 nm and 562 to ca. 510 nm in **1**^**3+**^. The blue-shift reflects the decreasing energy of the TAT-based donor MOs as more and more electron-donating ferrocenyl residues are altered to ferrocenium acceptors. Transitions with the same mixed CT/IVCT character are also discerned at higher energy and even in the near UV, as exemplified by the computed transitions at 471, 385, and 367 nm for **1**^**+**^ (see also Figure S53).

Our calculations even reproduce the spectral features of tetracation **1**^**4+**^ with the additional positive charge on the TAT scaffold (see Figure S56). **1**^**4+**^ was computed to adopt a quintet ground state with four unpaired spins. The transitions at the lowest energies retain their Fe d_δ/δ^*^_ character. The structured band at a computed wavelength of 1067 nm, which is the equivalent of the experimentally observed, structured absorption that stretches from 800 to ca. 1300 nm, can be identified as the IVCT band reported by Gopidas and co-workers for the tris-*N*-octyl-TAT^+^ radical cation.^30^ The band at a calculated wavelength of 760 nm (exp. value 795 nm) can likewise be identified as arising from a π → π* transition within the oxidized TAT^+^ core. Excitation into this band shifts electron density from all three peripheral phenyl rings to the central benzene ring of the TAT^+^ scaffold.

All the assets of **1**^***n*+**^ are shared by the other triferrocenyl-TATs **2** and **3** with only moderate shifts in the peak positions and intensities (Table 2), so we assume identical band assignments. The blue-shift of the CT bands in **3**^**3+**^ as compared to those in **1**^**3+**^ suggests that the TAT-based donor orbitals are displaced to lower energies, which also matches with the higher experimental TAT redox potential (Table 1).

On introducing the ethynylene spacers, as present in compounds **6** and **7**, the weak vis bands associated with the Fe → TAT CT character remain unaffected, while the TAT π → π* absorption shifts by 11 nm (900 cm^–1^; **6** vs **1**) or 18 nm (1640 cm^–1^, **7** vs **3**) to lower energy as a result of the larger extension of the π-conjugated template. In the trioxidized state, the energies of the Fe d_δ/δ^*^_ and the CT/IVCT transitions are increased when compared to their directly linked analogues. Again, the energies of these transitions are higher for 3-isomer **7**^**3+**^ than for 2-isomer **6**^**3+**^. The blue-shift of the ferrocenium-localized NIR transitions signals that an ethynyl linker enhances the splitting between the π-antibonding δ* and the less-interacting δ orbitals. The blue-shift of the TAT → Fc^+^ CT band seems, however, counterintuitive when referring to the trends in the redox potentials. The small half-wave potential separation between the TAT and the ferrocene oxidations in **6** and **7** suggests a small energy gap between the Fc^+^ acceptor and the TAT donor orbitals, so that one would expect a red-shift of the corresponding CT bands. We thus conclude that the large anodic shift of the half-wave potential for TAT oxidation in triferrocenyl-TATs with direct ferrocenyl-TAT C–C linkages is due to the repulsive Coulomb interactions. Thus, the electrostatic effects override the intrinsic effects of the ethynyl substituents, which one would otherwise expect to increase the potential of TAT oxidation. Such Coulombic effects will be particularly pronounced in the presence of the very weakly coordinating [B{C_6_H_3_(CF_3_)_2_–3,5}_4_]^−^ counteranion.^74^

We note in this vein an anodic shift of the TAT redox couple by ca. 20 mV for every added ethynyl substituent in the order **2-A**_**1**_**-**^**Et**^**TAT** < **2-A**_**2**_**-**^**Et**^**TAT** < **2-A**_**3**_**-**^**Et**^**TAT** (Table 1), indicating that the occupied TAT-based frontier MOs indeed decrease in energy on consecutive ethynyl substitution. The contribution of the ethynyl substituent to the HOMO of **2-A**_**1**_**-**^**Et**^**TAT** is reflected by the DFT calculations (see Figure S58). We also note that, contrary to the ethynyl-induced anodic shift of half-wave potential in the series of the 2-, 2,2′- and 2,2′,2″-mono/di/tri(ethynylated) TATs, the TAT oxidation in **6**^**3+**^ and **7**^**3+**^ occurs at 113 and 163 mV lower potential than in **1**^**3+**^ and **3**^**3+**^. An increasing stabilization of the TAT donor MOs with a higher degree of ethynyl substitution is also in line with the gradual blue-shift of the TAT → Fc^+^ CT band in the series **4**^**+**^ < **5**^**2+**^ < **6**^**3+**^ (see Table 2).

In their IR spectra, complexes **4**–**7** also offer the diagnostic C≡C stretching vibration(s) of the ethynyl linker(s), whose intensity depends strongly on the difference in polarity around the ethynyl bond. Figures S48 and S49 and Table S1 summarize the intensity and position changes during the individual oxidations as obtained from IR SEC experiments. For the neutral molecules, the C≡C band is too weak to be observed. Gradual oxidation of the ferrocenyl moieties provides a highly intense band, which is located at 2198 cm^–1^ for **6**^**3+**^. For **7**^**3+**^, a small splitting between the symmetric and asymmetric combinations of the C≡C stretching vibrations can be discerned. Subsequent TAT oxidation decreases the oscillator strength of the alkynyl band and causes a slight blue-shift by ca. 10 cm^–1^. The unsymmetrically substituted mono- and diethynylated complexes **4**^**2+**^ and **5**^**3+**^ retain a higher intensity of the C≡C stretching vibration after TAT oxidation than their trisubstituted counterparts **6**^**4+**^ and **7**^**4+**^.

### On-Surface STM-Switching of Ferrocenyl-Appended TATs 4–6 on Ag(111)

An important motivation for the present study was to probe whether and how the appended ferrocenyl substituents affect the on-surface switching of ^**Et**^**TAT** molecules on Ag(111). For this purpose, mono-, di-, and tri(ferrocenylethynyl)-modified TATs **4**–**6** were deposited on the Ag(111) substrate, kept at room temperature, via electrospray deposition (ESD).^95^

**Figure 8:**
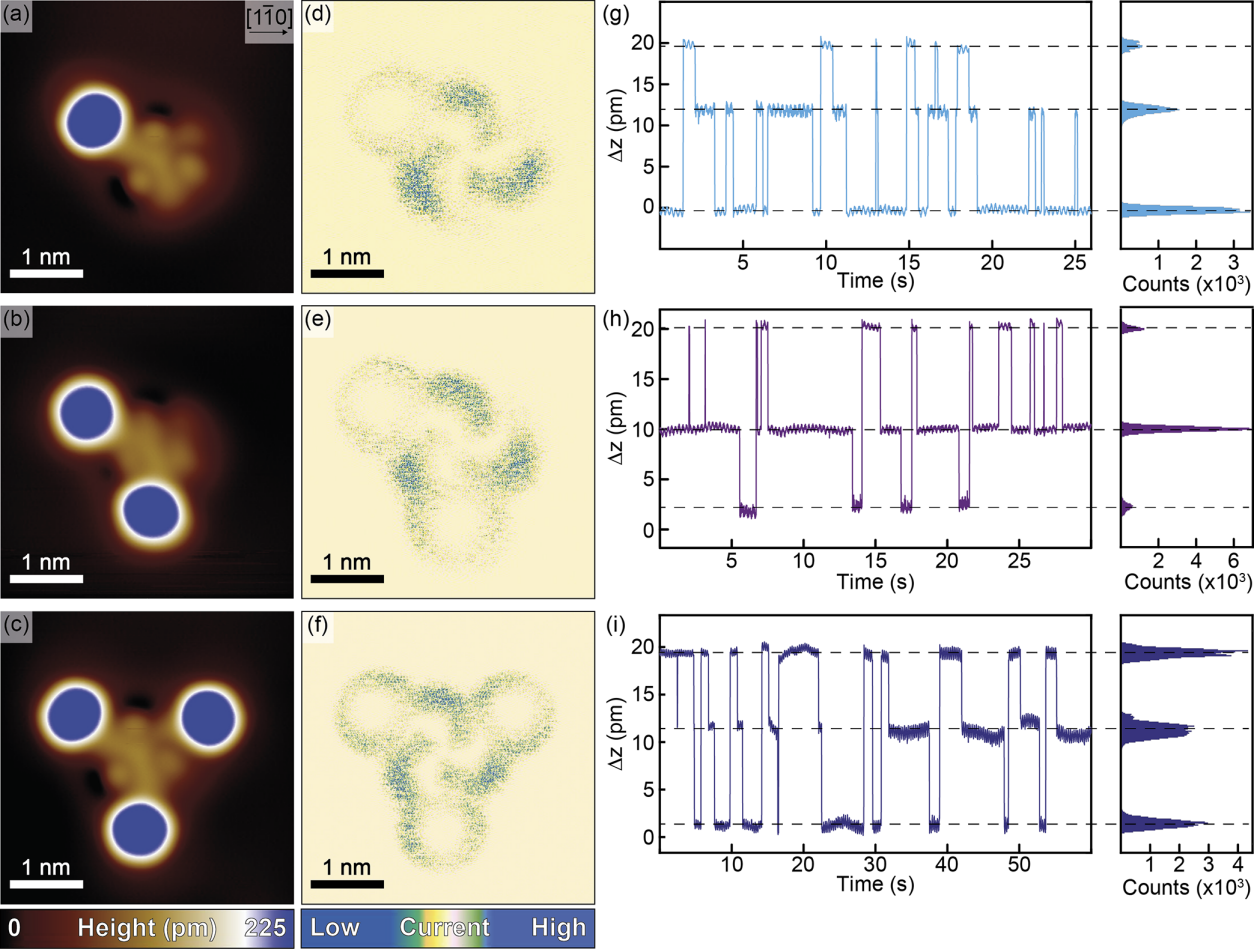
(a–c) Close-up topographic STM images of **4**–**6** on Ag(111). Scanning parameters: 4 × 4 nm^2^, *I* = 50 pA, *U* = 10 mV, and *T* = 5.9 K. (d–f) Corresponding tunneling current images showing the conductance fluctuations due to molecular switching. The chirality of the noise clouds identifies the molecules as *R* enantiomers. Scanning parameters: *I* = 500 pA, *U* = 200 mV, and *T* = 5.9 K. (g–i) Time traces of the *z*-position of the scanner recorded on the TAT cores of **4**–**6**. On the right side, the height histograms showing the occupation time of the individual states are presented. Measurement parameters: *I* = 100 pA, *U* = 60 mV, and *T* = 5.9 K.

Figure 8a–c shows close-up topographic STM images of the ferrocenylethynyl-appended TATs **4**–**6** on Ag(111) in the *R* surface enantiomer configuration. The TAT core is readily recognized by the characteristic 3-fold symmetric molecular backbone surrounded by the three distinct circular-shaped maxima, which are associated with the *N*-bonded ethyl groups. Close inspection of the images in Figure 8a–c shows the depletion areas resulting from charge redistribution between the TAT molecule and the substrate at only two of the three maxima. This particular pattern originates from a tilting of the TATs and the local differences in the N–Ag bond lengths, which are themselves due to a slight mismatch between the positions of the TAT N atoms and the Ag atoms of the substrate.^42^ Taking into account the 3-fold symmetry of the TAT core, three bonding configurations within the same adsorption geometry exist, depending on which the N atom is further lifted from the underlying Ag atoms. The ferrocenyl substituents of compounds **4**–**6** are readily discerned as round protrusions appearing in dark blue color in Figure 8a–c with an apparent height of about 225 pm. Their apparent height changes to ca. 300 pm at 1.8 V (see Figure S64), which agrees with the results of STM measurements on other surface-confined ferrocenes.^96–98^

As presented in Figures 8d–f, S59, and S60, increasing the bias voltage and the current induces switching between the three adsorption configurations of the TAT core unit, as discussed above. The changes in the tilting of the entire molecule during this process lead to fluctuations of the tunneling conductance and accordingly to variations of the *z*-position of the STM scanner.^42^ Configurational on-surface switching is then visible in the tunneling current images as noise clouds localized at the positions of the amine *N* atoms and ethyl side arms as they move closer toward or further away from the substrate. The behavior of **4**–**6** is phenomenologically identical to that of pristine ^**Et**^**TAT**, confirming that on-surface switching is fully retained, notwithstanding the appended ferrocenylethynyl residues. Obviously, the variations of the adsorption geometry upon switching are not confined to the TAT core but extend over the entire molecule. This can be deduced from the noise clouds, which are spread over the whole molecule, including the ferrocenylethynyl residues.

**Figure 9:**
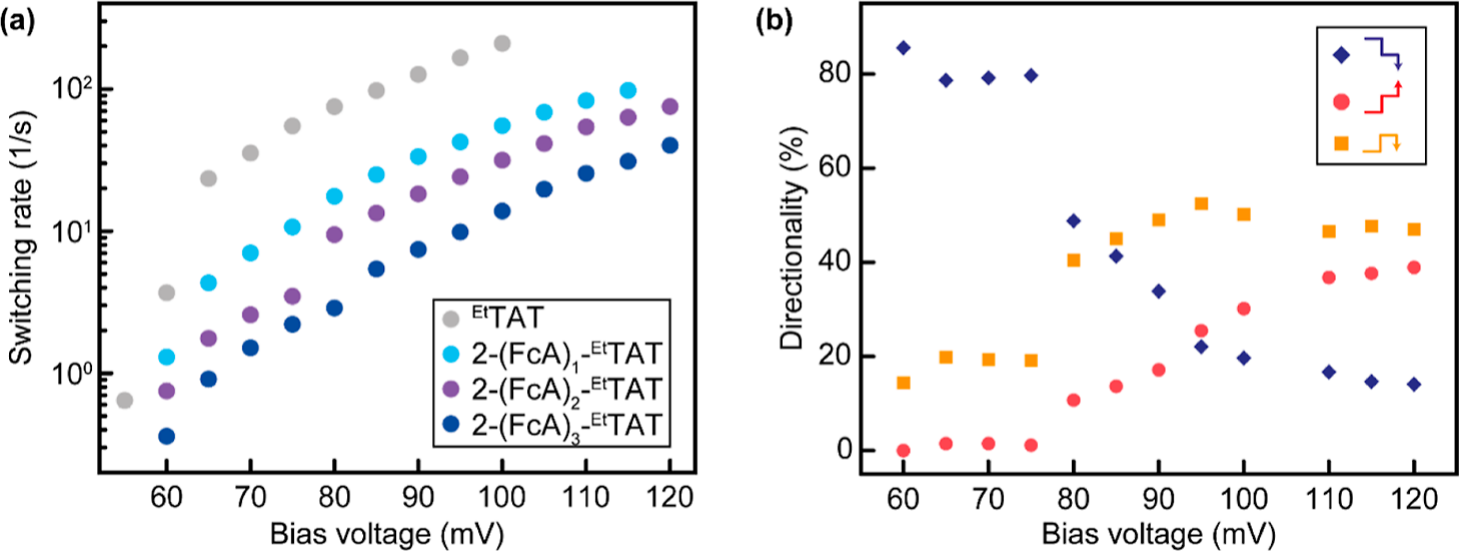
Switching characteristics of **2-(FcA)**_***n***_**-TAT**. (a) Bias voltage-dependent switching rate *r*(*U*) [s^–1^] of **2-(FcA)**_***n***_**-TAT** (*n* = 0 to 3) and ^**Et**^**TAT** at *I*_set_ = 100 pA. The measurements were performed on a noise cloud of the TAT core. (b) Switching directionality of the *R* surface enantiomer of **2-(FcA)**_**3**_**-TAT** (**6**) in percent measured on the TAT core and its dependence on the bias voltage. The blue diamonds represent the sequence high → middle → low (HML), the red dots represent the inverse sequence low → middle → high (LMH), and the orange squares represent nondirectional two-state switching. The measurements were performed at *I*_set_ = 100 pA.

We further investigated the switching behavior of **4**–**6** by positioning the STM tip above a molecule and recording the time dependence of the *z*-position of the tip. Figure 8g–i displays the background-corrected *z*(*t*)-traces recorded within the noise clouds of the TAT cores of **4**–**6**. All *z*(*t*)-traces show a three-level telegraph noise signal corresponding to the switching between the three adsorption states associated with minima of the potential landscape of the system. The switching characteristic of trisubstituted **6**, where these energy minima are degenerate, closely resembles that of pristine ^**Et**^**TAT** with roughly equal occupation times for all the three states (Figure 8i). However, the bias-dependent switching rates are slightly reduced compared to those of ^**Et**^**TAT**, which results from enhanced coupling to the substrate owing to the ferrocenyl substituents (see Figures 9a and S59 and S60). For monoferrocenyl compound **4**, we observe a strong decrease of the occupation time for one of the states. We attribute this to the additional pinning due to the ferrocenyl unit, which modifies the adsorption potential with one energy minimum being pushed up in energy, thus leading to an effective two-level switching between the low-lying minima. Adding a second ferrocenyl unit (compound **5**) leads thus to a potential landscape with one low-energy minimum and two higher-lying minima, which results in long occupation times for the preferred state. As expected, the overall switching rate under identical conditions decreases in the order ^**Et**^**TAT** > **4** > **5** > **6** according to the increasing coupling between the molecule and the substrate (Figure 9a).

Importantly, not only the switching behavior itself but also its high level of directionality at low bias are preserved as exemplified by the time traces in Figure 8g–i. The *z*(*t*) measurements show that the repetition of the same sequence of states (low-high-middle-low) is strongly preferred for all of the investigated compounds. The directionality of switching upon excitation by inelastic electron tunneling events can be understood in the frame of the Brownian ratchet theory,^99^ where the excitation and subsequent relaxation into an asymmetric potential lead to a directionality. In order to statistically analyze the data regarding the switching directionality, we count all two-step switching sequences reflecting the three successive states related by two transition steps between these states and plot the normalized result for each set of measurement parameters. Figure 9 together with Figures S61–S63 shows the results of such an analysis for compounds **4**–**6**. Data recorded at the TAT core of the surface *R* enantiomer of **6** (Figure 9b) indicate a directionality of ca. 86% at low bias (60 mV), which is only modestly inferior to the value of 94% realized by ^**Et**^**TAT** under identical conditions.^42^ Directionality is retained for switching events that are triggered by placing the STM tip over the ferrocenyl unit (Figure S61). The deduced directionality for on-surface switching amounts to about 80%, which compares well with the value of 86% at the position of the TAT core. The results of the STM studies on mono- and di(ferrocenylethynyl) TATs **4** and **5**, which lack the 3-fold molecular symmetry, are displayed in Figures S62 and S63. The data indicate that nondirectional two-state switching dominates (>60%) at all investigated bias voltages from 60 to 120 mV, irrespective of over which ethyl group the measurements are performed. This indicates that the potential landscape underlying the switching is modified as discussed above, thus effectively suppressing the three-level switching. For sequences that correspond to directional switching, clockwise switching at low bias and counterclockwise switching at high bias are observed, roughly following the trend of compounds **6** and ^Et^**TAT**. Our STM results thus indicate that symmetrically trisubstituted TATs with paramagnetic and, in particular, magnetically anisotropic metallocenyl residues are viable candidates for active trit units, whose switching behavior can be controlled through electric stimuli and, possibly, magnetic fields.

### Summary and Conclusions

We reported the preparation and characterization of seven ferrocenyl-substituted triazatruxenes (TATs) **1**–**7**. They belong to a relatively unexplored compound class, which combines polycyclic aryl-substituted amines (PAAs) with the versatile ferrocene motif. The newly synthesized **Fc-TATs** differ with respect to whether the ferrocenyl units are directly linked to the TAT core (**1**–**3**) or whether they connect via an ethynylene spacer (**4**–**7**), the positioning of the ferrocenyl substituents and, for the series of 2-ethynylferrocenyl-substituted *N*-ethyl TATs, the degree of substitution. We also developed a convenient route that provides pure samples of mono- and diethynylated TATs en route to complexes **4** and **5** by installing the 2-methylbut-3-yn-2-ol (mebynol) functionality, whose polarity allows for easy chromatographic separation of the monosubstituted and disubstituted congeners.

With compounds **1**–**7** in hand, we investigated their redox properties and their optical UV/vis- and NIR-excitations in different redox states. All investigated TATs undergo one reversible one-electron oxidation per ferrocenyl residue, which is followed by one additional oxidation of the TAT core. The potential for TAT oxidation is subject to the electronic and electrostatic effects. The latter becomes particularly important in complexes **1**–**3** with direct ferrocenyl-TAT linkages. We also note additive substituent effects in mono-, di-, and tri(ethynylferrocenyl)-TATs **4** to **6**.

We then probed the impact of stepwise oxidations of the ferrocenyl units to ferrocenium cations and of the TAT core by UV/vis/NIR spectroscopy under in situ conditions of spectroelectrochemistry. Oxidation of the ferrocenyl residues gives rise to fairly intense absorption in the near-infrared (NIR), which gradually shifts to higher energies as more ferrocenyls are oxidized. Our TD-DFT calculations on **1**^***n*+**^ (*n* = 1, 3) as a representative example assign these bands to d_δ/δ^*^_ transitions that are largely confined to the ferrocenium units. This is in line with previous observations on ferrocenes with appended aryl substituents.^89^ TD-DFT studies on cation **1**^**+**^ with only one oxidized ferrocenyl unit indicate that intervalence charge-transfer (IVCT) from the remaining, reduced ferrocenyl moieties to the oxidized one contributes to higher energy absorption bands, where it mingles with the TAT → Fc^+^ charge-transfer excitations. Our calculations even provide a good match between the calculated and experimental spectra of **1**^**4+**^, which is computed to adopt a quintet ground state.

Most importantly, ferrocenyl-substituted TATs retain the propensity of parent ^**Et**^**TAT** for on-surface switching on Ag(111), as probed by the STM methods. The switching relies on the skewed adsorption geometry of the TAT molecules on this substrate with two shorter and one longer N–Ag bonds, which itself is due to a slight mismatch between the atomic positions of the indolyl *N* and the Ag surface atoms. Just like ^**Et**^**TAT** itself, trisubstituted **6** shows directional switching between the three degenerate states at low bias voltages, albeit with a slightly diminished reliability (86% versus 93.6% for the *R* surface enantiomer) and a lower switching rate due to the stronger bonding to the substrate. We observe the same switching behavior, irrespective of whether the STM tip is positioned next to a ferrocene protrusion or next to one of the noise clouds near an N atom of the TAT core. Desymmetrization of the TAT core in monoferrocenyl-substituted **4** leads to dominant two-state switching between two degenerate states, which each have a short Ag–N contact to the ferrocenyl-substituted indolyl ring. For disubstituted **5**, we observed a strong suppression of the switching, leading to large occupation times for one single state, namely, the one where both ferrocenyl-substituted indolyl rings are close to the surface. Thus, the results of the present study confirm that TATs with appended magnetically anisotropic metallocenyl substituents are viable candidates as magnetically and electrically addressable, active trit units for high-density on-surface information storage.

## Supporting Information Available

Methods, materials and instrumentation, synthesis procedures, and characterization data including NMR and HR ESI-MS, cyclic and square wave voltammograms, results of UV/vis/NIR and IR spectroelectrochemical studies, TD- DFT calculations on **1**, **1^+^**, **1^3+^**, and **1^4+^**, and STM experiments for on-surface switching of molecules **4**–**6** on Ag(111) (PDF)
