# Supplementary material for: Ferrocenyl-Substituted Triazatruxenes: Synthesis, Electronic Properties, and the Impact of Ferrocenyl Residues on Directional On-Surface Switching on Ag(111)
Source: Inorg Chem. 2023 Sep 21;62(39):16236–49. doi: 10.1021/acs.inorgchem.3c03009 (PMC10548419; doi:10.1021/acs.inorgchem.3c03009)
Supplement: Supplementary file 1 — ic3c03009_si_001.pdf [file ic3c03009_si_001.pdf]

Ferrocenyl-Substituted Triazatruxenes: Synthesis, Electronic Properties and Impact of Ferrocenyl Residues on Directional On-Surface Switching On Ag(111)

Lars Vogelsang,[a] Tobias Birk,[b] Fabian Paschke,[b] Anja Bauer,[b] Vivien Enenkel,[b] Lukas M. Holz,[a] Mikhail Fonin[b]*, and Rainer F. Winter[a]*

[a] *Fachbereich Chemie, Universität Konstanz, 78457 Konstanz, Germany*
[b] *Fachbereich Physik, Universität Konstanz, 78457 Konstanz, Germany*

Email: rainer.winter@uni-konstanz.de; mikhail.fonin@uni-konstanz.de

Supporting information

List of contents

Materials and Methods 2
NMR spectroscopy 2
Mass spectrometry 2
Electrochemical investigations 2
DFT-calculations 3
STM 4
Chemical Synthesis and Characterization 4
NMR and Mass Spectra 17
Voltammetric measurements 34
UV/Vis/NIR and IR Spectroelectrochemistry 37
DFT Calculations 42
STM switching 49
References 52

Materials and Methods

NMR spectroscopy

NMR-spectra were recorded on either a Bruker Avance III 400 (^{1}H-NMR: 400 MHz, ^{13}C-NMR: 101 MHz), a Bruker Avance Neo 800 (^{1}H-NMR: 800 MHz, ^{13}C-NMR: 202 MHz), a Bruker Avance III 600 (^{1}H-NMR: 600 MHz, ^{13}C-NMR: 152 MHz) or a Jeol JNM-ECZR (^{1}H-NMR: 500 MHz), ^{13}C-NMR: 121 MHz) spectrometer. All spectra were recorded in deuterated solvents. The spectra were referenced to the residual protonated solvent for ^{1}H-NMR spectra or the ^{13}C-signal of the deuterated solvent for ^{13}C-NMR spectra.

Mass spectrometry

Mass spectrometry was performed in CH_2Cl_2 with a *LTC Orbitrap Velos* by *Thermo Fischer Scientific* in positive ion mode with ESI. The minimum resolution was 60000 FWHM. Precalibration was done with a *Pierce LTQ Velos Es* Positive Solution by *Thermo Fischer Scientific*. Simulation was performed using the natural abundance of the respective elements using the software Xcalibur™ by *Thermo Fischer Scientific.*

Electrochemical investigations

Cyclic voltammograms were measured under an argon atmosphere in the $CH_2Cl_2/NBu_4^+[B\{C_6H_3(CF_3)_2\text{-}3,5\}_4]^-$ electrolyte. $Na^+[B\{C_6H_3(CF_3)_2\text{-}3,5\}_4]^-$ was prepared from 3,5-bis(trifluoromethyl)bromobenzene according to a published procedure,[1] followed by the exchange of the Na^+ cation for NBu_4^+.[2] Voltammetry was performed using a computer-controlled *BASi* potentiostat. A custom-made cylindrical, vacuum-tight, single-compartment cell equipped with a platinum working electrode was utilized. A spiral-shape platinum wire was employed as the counter electrode and a silver wire as the (pseudo)reference electrode. These electrodes were sealed into glass capillaries and fixated to sidearms of the cell using Quickfit screws. The working electrode was polished with diamond pastes of 1 µm and 0.25 µm grain size prior to measurement. The working electrode was inserted into the top port of the cell through a Quickfit fitting. Referencing was performed using either decamethylferrocene (Cp^*_2Fe, $E_{1/2}$ = - 550 mV vs $FcH^{0/+}$) or cobaltocenium hexafluorophosphate ($[Cp_2Co]^+[PF_6]^-$, $E_{1/2}$ = -1330 mV vs $FcH^{0/+}$) as internal calibrant. Potentials are provided relative to the ferrocene/ferrocenium (FcH/FcH^+) redox couple.

Digital simulations of cyclic voltammograms were performed with DigiSim.[3] The electrode surface was set to 0.0256 cm^{-3}. The diffusion was assumed to be semi-infinite and the simulation was performed with pre-equilibration. All redox reactions were assumed to be homogeneous. The parameters used/obtained in the fitting procedure are given in Table S1.

*Table S1. Fitting parameters for the simulation of cyclic voltammograms of **1** and **2**.*

	**2-Fc_3-TAT (1)**	**2-Fc_3-^{NDode}TAT (2)**
Initial concentration of the neutral complex (c)	2.90 mmol/L	1.30 mmol/L
Diffusion coefficient of c	$1.26 \bullet 10^{-6}$ cm^2/s	$1.0 \bullet 10^{-6}$ cm^2/s
Diffusion coefficient of c^+	$1.26 \bullet 10^{-6}$ cm^2/s	$1.0 \bullet 10^{-6}$ cm^2/s
Diffusion coefficient of c^{2+}	$1.04 \bullet 10^{-6}$ cm^2/s	$1.0 \bullet 10^{-6}$ cm^2/s
Diffusion coefficient of c^{3+}	$1.05 \bullet 10^{-6}$ cm^2/s	$1.0 \bullet 10^{-6}$ cm^2/s
Rate constant k_s of c^+ + e = c	0.021 cm/s	0.020 cm/s
Rate constant k_s of c^{2+} + e = c^+	0.029 cm/s	0.040 cm/s
Rate constant k_s of c^{3+} + e = c^{2+}	0.031 cm/s	0.040 cm/s
Cdl - value	$1.50 \bullet 10^{-7}$	$2.0 \bullet 10^{-7}$
α/λ of c^+ + e = c	0.74 eV	0.30 eV
α/λ of c^{2+} + e = c^+	0.029 eV	0.50 eV
α/λ of c^{3+} + e = c^{2+}	0.021 eV	0.50 eV
E_0 in mV of c^+ + e = c	-133 mV	-88 mV
E_0 in mV of c^{2+} + e = c^+	-66 mV	-13 mV
E_0 in mV of c^{3+} + e = c^{2+}	0 mV	41 mV

UV/Vis/NIR Spectroscopy and Spectroelectrochemistry

FT-IR spectra were recorded on a *Bruker* Tensor III instrument in a range between 1000 cm^{-1} to 11500 cm^{-1}. UV/Vis/NIR spectra were measured with a *TIDAS* fiber optic diode array spectrometer, which combines MCS UV/Vis and PGS NIR instruments from *J&M*. Extinction coefficients were determined in Hellma quartz cuvettes with 0.1 cm and 0.2 cm thickness. Spectroelectrochemical measurements were performed using an OTTLE (optically transparent thin-layer electrochemical) cell according to the design of Hartl et al.[4] The cell is custom-built with CaF_2 windows, Pt-mesh working and counter electrodes and a Ag/AgCl pseudo-reference electrode. The measurements were recorded in a dry and degassed $NBu_4^+[B\{C_6H_3(CF_3)_2\text{-}3,5\}_4]^-$/1,2-$C_2H_4Cl_2$ electrolyte. Potentials were applied using a *Wenking Pos 2* potenstiostat by *Intelligent Controls GmbH*.

DFT-calculations

Quantum chemical calculations on **2-Fc_3-TAT** were performed using the GAUSSIAN 16 program package.[5] Electronic transitions were obtained by the time-dependent DFT approach (TD-DFT). For iron, the MDF-10 basis set was applied.[6] For all other atoms triple-ζ basis sets (6-31G(d))[7] were used for structural optimization and construction of the corresponding molecular orbitals. For all calculations the PBE1PBE functional was used.[8] Solvent effects were modelled using the polarizable conductor continuum model (PCCM).[9] Molecular orbitals are depicted in blue and white for positive and negative signs of the wavefunctions.

STM

The STM samples were prepared in situ. The Ag(111) crystal (Surface Preparation Laboratory B. V.) was cleaned by repeated cycles of Ar^+ sputtering (2kV) and annealing to 600 °C. Solid samples of **2-(Fc-A)$_n$-^{Et}TAT** were dissolved in dichloromethane and methanol and subsequently deposited *via* electrospray deposition (ESD) at room temperature. The used ESD setup is described in ref. [10]. In brief, dissolved molecules are accelerated under ambient pressure by a voltage drop. The solvent with the probe molecules is polarized by applying a high voltage (~kV) to the emitter tip. A jet of charged particles is accelerated into the vacuum and can be manipulated by an atmospheric pressure electrode (APE) and counter flow of N_2 gas. The amount of deposited material is monitored via current measurements. Measurements were performed in a two-chamber ultra high vacuum (UHV) system operating at $5{\cdot}10^{-11}$ mbar) with an *Omicron* Cryogenic-STM at 3 to 6 K in constant current mode. For all measurements, grinded and polished PtIr tips (*Nanoscore GmbH*) were used.[10]

Chemical Synthesis and Characterization

All syntheses were carried out under nitrogen atmosphere using common Schlenk techniques. Solvents were dried over appropriate drying agents, deoxygenated by purging with dinitrogen or by three freeze-pump-thaw cycles, and stored under nitrogen. All starting materials were purchased from commercial suppliers and used without further purification.

6-Bromo-N-ethylisatin[11]

10.00 g of 6-bromoisatin (44.24 mmol, 1.0 eq.) and potassium carbonate (23.44 g, 169.61 mmol, 3.8 eq.) were suspended in 85 mL of dimethylformamide. Subsequently, 20 mL of iodoethane (38.80 g, 248.77 mmol, 5.6 eq.) were added. The reaction mixture was stirred at 70 °C for 6 hours. After cooling to room temperature, 100 mL of H_2O were added and the aqueous phase was extracted with CH_2Cl_2 (3×100 mL). The combined organic layers were dried over $MgSO_4$ and the solvent was removed under reduced pressure. 10.78 g of 6-bromo-*N*-ethylisatin were obtained as a red solid (42.43 mmol, 96%).

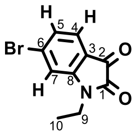

^{1}H-NMR ($CDCl_3$, 500 MHz) δ 7.46 (d, $^3J_{HH}$ = 7.92 Hz, 1H, H-4), 7.27 (dd, $^3J_{HH}$ = 7.92, 1.56 Hz, 1H, H-5), 7.08 (d, $^3J_{HH}$ = 1.56 Hz, 1H, H-7), 3.77 (q, $^3J_{HH}$ = 7.26 Hz, 2H, H-9), 1.32 (t, $^3J_{HH}$ = 7.26 Hz, 3H, H-10).

6-Bromo-N-ethyloxindole[11]

10.78 g of 6-bromo-*N*-ethylisatin (42.43 mmol, 1.0 eq.) were suspended in 55 mL of hydrazine monohydrate. The reaction mixture was stirred under reflux conditions for 5 hours. After cooling to room temperature, 100 mL of H_2O were added and the aqueous phase was extracted with CH_2Cl_2 (3×100 mL). The combined organic layers were dried over $MgSO_4$ and the solvent was removed under reduced pressure. 9.40 g of 6-bromo-*N*-ethyloxindole were obtained as a light orange solid (39.15 mmol, 92%).

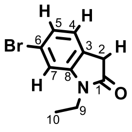

^{1}H-NMR ($CDCl_3$, 400 MHz,) δ (ppm): 7.16 (dd, $^{3}J_{HH}$ = 7.9, 1.7 Hz, 1H, H-5), 7.09 (d, $^{3}J_{HH}$ = 7.9 Hz, 1H, H-4), 6.97 (d, $^{3}J_{HH}$ =1.7 Hz, 1H, H-7), 3.73 (q, $^{3}J_{HH}$ =7.2 Hz, 2H, H-2), 3.45 (s, 2H, H-9), 1.26 (t, $^{3}J_{HH}$ =7.2 Hz, 3H, H-10).

2,2',2"-Tribromo-N,N',N"-triethyltriazatruxene (**2-Br$_3$-^{Et}TAT**)[11]

6-Bromo-N-ethyloxindole (9.40 g, 39.15 mmol, 1.0 eq.) was suspended in 50 mL of phosphorus oxychloride. The reaction mixture was stirred under reflux conditions for 4 hours. After allowing to cool to room temperature, the mixture was poured slowly onto 1000 g of crushed ice. The mixture was extracted with CH_2Cl_2 (4×100 mL). The combined organic phases were dried over $MgSO_4$ and the solvent was removed under reduced pressure. The black residue was purified *via* column chromatography with a mixture of CH_2Cl_2 : petroleum ether (4:1) as the eluent. The fractions containing the desired product were combined, the solvents removed and the residue was washed with hexane (5×30 mL). 2,2',2"-Tribromo-*N,N',N"*-triethyltriazatruxene was obtained as a beige solid (2.26 g, 3.39 mmol, 26%).

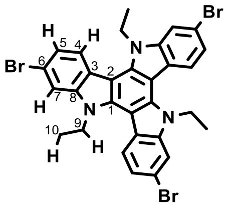

^{1}H-NMR ($CDCl_3$, 400 MHz,) δ (ppm): 8.13 (dd, $^{3}J_{HH}$ = 8.6, $^{4}J_{HH}$ = 2.3 Hz, 3H, H-5), 7.77 (d, $^{4}J_{HH}$ = 2.3 Hz, 3H, H-7), 7.45 (d, $^{3}J_{HH}$ = 8.6 Hz, 3H, H-4), 4.92 (q, $^{3}J_{HH}$ =7.2 Hz, 6H, H-9), 1.58 (t, $^{3}J_{HH}$ =7.2 Hz, 9H, H-10).

2,2',2"-Triferrocenyl-N,N',N"-triethyltriazatruxene (**2-Fc$_3$-^{Et}TAT**)

Ferrocenyl boronic acid (0.29 g, 1.26 mmol, 6.0 eq.) and **2Br$_3$-^{Et}TAT** (0.14 g, 0.21 mmol, 1 eq.) were placed in a Schlenk tube and dissolved in 12 mL of dimethoxyethane, which had been degassed by flushing with nitrogen for 20 minutes. A solution of 0.1 g NaOH in 2.5 mL of degassed H_2O and 15 mg of Pd(dppf)Cl_2 was added and the mixture was left stirring for 1 week at 80 °C. After cooling to room temperature, 100 mL of CH_2Cl_2 and 50 mL of H_2O were added. The phases were separated and the aqueous phase was extracted with 3×50 mL of CH_2Cl_2. The combined organic phases were dried over Na_2SO_4 and filtered through a plug of Celite using CH_2Cl_2 as the eluent. The solvents were removed under reduced pressure and the residue was extracted into diethyl ether and filtered. The solvent was removed under reduced pressure and the orange solid was purified *via* column chromatography with CH_2Cl_2 : petroleum ether (1:1) as the eluent. The product was obtained as an orange solid (40 mg, 0.04 mmol, 20%). Other fractions contained small amounts of pure 2,2'-diferrocenyl-*N,N',N"*-triethyltriazatruxene and 2-ferrocenyl-*N,N',N"*-triethyltriazatruxene.

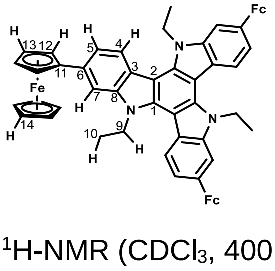

^{1}H-NMR ($CDCl_3$, 400 MHz) δ 8.26 (d, $^{3}J_{HH}$ = 8.4 Hz, 3H, H-4), 7.72 (d, $^{4}J_{HH}$ = 1.5 Hz, 3H, H-7), 7.54 (dd, $^{3}J_{HH}$ = 8.4, $^{4}J_{HH}$ = 1.5 Hz, 3H, H-5), 5.06 (q, $^{3}J_{HH}$ = 7.2 Hz, 6H, H-9), 4.83 (vt, J_{HH} 1.8

Hz, 6 H, H-12/13), 4.40 (vt, J_{HH} = 1.8 Hz, 6H, H-12/13), 4.14 (s, 15H, H-14), (t, $^{3}J_{HH}$ = 7.2 Hz, 9H, H-10).

$^{13}C\{^{1}H\}$ NMR ($CDCl_3$, 101 MHz) δ 141.4 (s, C-8), 138.7 (s, C-1), 134.2 (s, C-3), 122.0 (s, C-6), 121.4 (s, C-4), 119.1 (s, C-5), 107.6 (s, C-7), 103.7 (s, C-2), 87.1 (s, C-11), 69.8 (s, C-14),68.9 and 66.8 (each s, C-12, C-13), 41.8 (s, C-9), 15.8 (s, C-10).

5-Bromo-N-ethylisatin[11]

5-Bromoisatin (10.07 g, 44.55 mmol, 1.0 eq.) and K_2CO_3 (23.40 g, 169.30 mmol, 3.8 eq.) were suspended in 85 mL of dimethylformamide. 20 mL of iodoethane (38.80 g, 248.77 mmol, 5.6 eq.) were added. The reaction mixture was stirred for 6 hours at 70 °C. After allowing the reaction mixture to cool to room temperature, 400 mL of H_2O were added. The mixture was extracted with CH_2Cl_2 (4×100 mL). The combined organic phases were dried over $MgSO_4$ and the solvents were removed under reduced pressure. The product was obtained as a dark red solid in a yield of 96% (10.89 g, 42.86 mmol).

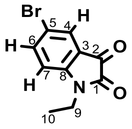

^{1}H-NMR ($CDCl_3$, 400 MHz) δ 7.72-7.67 (m, 2H, H-4, H-6), 6.82 (d, $^{3}J_{HH}$ = 8.20 Hz, 1H, H-7), 3.77 (q, $^{3}J_{HH}$ = 7.26 Hz, 1H, H-9), 1.30 (t, $^{3}J_{HH}$ = 7.26 Hz, 1H, H-10).

5-Bromo-N-ethyloxindole[11]

10.89 g of 5-bromo-*N*-ethylisatin (42.86 mmol, 1.0 eq.) were suspended in 55 mL of hydrazine monohydrate. The reaction mixture was stirred under reflux for 5 hours. After cooling to room temperature, 100 mL of H_2O were added and the aqueous phase was extracted with CH_2Cl_2 (3×100 mL). The combined organic layers were dried over $MgSO_4$ and the solvent was removed under reduced pressure. 9.40 g of 5-bromo-*N*-ethyloxindole were obtained as a pale orange solid (39.15 mmol, 91%).

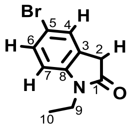

^{1}H-NMR ($CDCl_3$, 400 MHz) δ 7.43-7.34 (m, 2H, H-4, H-6), 6.71 (d, $^{3}J_{HH}$ = 8.20 Hz, 1H, H-7), 3.74 (q, $^{3}J_{HH}$ = 7.26 Hz, 1H, H-9), 3.5 (s, 2H, H-2), 1.25 (t, $^{3}J_{HH}$ = 7.26 Hz, 3H, H-10).

3,3',3''-Tribromo-N,N',N''-triethyltriazatruxene (**3-Br$_3$-^{Et}TAT**)[11]

6-Bromo-*N*-ethyloxindole (9.40 g, 39.15 mmol, 1.0 eq.) was suspended in 50 mL of phosphorus oxychloride. The reaction mixture was stirred under reflux conditions for 4 hours. After allowing to cool to room temperature the mixture was poured slowly onto 1000 mL of crushed ice. The mixture was extracted with CH_2Cl_2 (4×100 mL). The combined organic phases were dried over $MgSO_4$ and the solvent was removed under reduced pressure. The black residue was purified *via* column chromatography with CH_2Cl_2 : petroleum ether (4:1) as the eluent. Fractions that contained the desired product were combined and stripped of the solvents in vacuo. The solid

residue was washed with ⁿhexane (5×30 mL). 2,2',2''-Tribromo-*N,N',N''*-triethyltriazatruxene was obtained as a light yellow solid (4.60 g, 6.90 mmol, 53%).

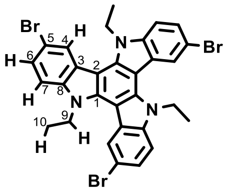

^{1}H-NMR ($CDCl_3$, 400 MHz) δ 8.36 (d, $^4J_{HH}$ = 1.8 Hz, 3H, J H-4), 7.50 (dd, $^3J_{HH}$ = 8.6 Hz, $^4J_{HH}$ = 1.8 Hz, 6H, H-6), 7.50 (d, 3H, $^3J_{HH}$ = 8.6 Hz, H-7), 4.92 (q, $^3J_{HH}$ = 7.20 Hz, 6H, H-9), 1.62 (t, $^3J_{HH}$ = 7.20 Hz, 9H, H-10).

3,3',3''-Triferrocenyl-N,N',N''-triethyltriazatruxene (**3-Fc$_3$-^{Et}TAT**)

In situ formation of the Negishi reagent: 0.34 g of ferrocene (1.80 mmol, 6.0 eq.) and 0.005 g of ^{t}BuOK (0.18 mmol, 0.15 eq.) were dissolved in 6 mL of dry THF. The reaction mixture was cooled to -78 °C. 2.40 mL of a 1.9 M solution of ^{t}BuLi in ^{n}pentane (4.50 mmol, 15.0 eq.) were added dropwise with stirring. After complete addition, stirring was continued for 1 h while the temperature was maintained at -78 °C. A solution of 0.46 g of $ZnCl_2$(TMEDA) (1.83 mmol, 6.0 eq.) in 7 mL of dry THF was then added and the solution was stirred for an additional hour while allowing the reaction mixture to warm to room temperature.

Negishi coupling: A solution of 3,3',3''-tribromo-*N,N',N''*-triethyltriazatruxene (0.20 g, 0.30 mmol, 1.0 eq.) and Pd(dppf)Cl_2 (20 mg) in 8 mL of dry THF was added. The reaction mixture was stirred under reflux for 1 week. The solvent was removed under reduced pressure and the residue was dissolved in diethyl ether. The organic phase was washed twice with H_2O. The organic phase was dried over Na_2SO_4 and the solvent was removed under reduced pressure. The residue was dissolved in dry CH_2Cl_2. For purification, ferrocenium hexafluorophosphate (>3-fold excess) was added to the solution. Crude 3,3',3''-triferrocenyl-*N,N',N''*-triethyltriazatruxene thereby reacts to the ether-insoluble triferrocenium salt. The mixture was sonicated, the solvent was removed and the residue was washed with diethyl ether (4×40 mL), which removes the formed ferrocene and organic by-products. The solid was redissolved in CH_2Cl_2 and excess zinc dust was added in order to reduce the 3,3',3''-triferroceniumyl-*N*,*N*',*N*''-triethyltriazatruxene back t the neutral form. After sonication and filtration from remaining zinc dust and $Zn(PF_6)_2$ the solvent was removed under reduced pressure. The remaining solid was washed with ^{n}pentane until the washings remained colorless. The crude product was further purified *via* column chromatography with CH_2Cl_2:^{n}pentane (40:60 to 60:40) as the eluent. The product was isolated as an orange solid (0.041 g, 0.042 mmol, 14%).

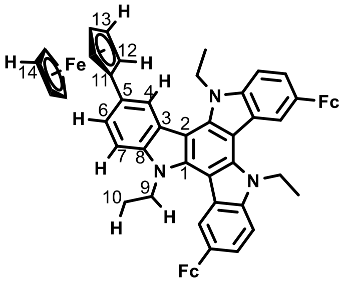

^{1}H-NMR (CD_2Cl_2, 400 MHz) δ 8.53 (d, $^4J_{HH}$ = 1.5 Hz, 3H, H-4), 7.72 (dd, $^3J_{HH}$ = 8.4, $^4J_{HH}$ = 1.5 Hz, 3H, H-6), 7.70 (d, $^3J_{HH}$ = 8.4 Hz, 3H, H-7), 5.13 (q, $^3J_{HH}$ = 7.2 Hz, 6H, H-9), 4.86 Hz (vt, *J*

= 1.8 Hz, 6 H, H-12/13), 4.43 (vt, J = 1.8 Hz, 6H, H-12/13), 4.14 (s, 15H, H-14), 1.84 (t, $^3J_{HH}$ = 7.2 Hz, 9H, H-10).

$^{13}C\{^1H\}$ NMR (CD_2Cl_2, 101 MHz) δ 140.1 (s, C-8), 139.3 (s, C-1), 130.9 (s, C-3) , 124.0 (s, C-7), 122.3 (s, C-6), 110.6 (s, C-5), 103.5 (s, C-2) , 88.0 (s, C-11), 69.9 (s, C-14), 69.0 (s, C-12/13), 66.7 (s, C-12/13), 42.4 (s, C-9) , 16.3 (s, C-10).

N-Ethyloxindole[12],[11]

Isatine (10.0 g, 67.97 mmol, 1.0 eq.) and K_2CO_3 (31.0 g, 224.29 mmol, 3.3 eq.) were suspended in 85 mL of DMF and 27 mL of iodoethane (53.00 g, 339.83 mmol, 5.0 eq.) were added. The reaction mixture was stirred at 70 °C for 5 hours. After cooling to room temperature, 400 mL of H_2O were added and the reaction mixture was extracted with CH_2Cl_2 (4×100 mL). The combined organic phases were dried over $MgSO_4$ and the solvent was removed under reduced pressure. *N*-Ethylisatin was obtained as a red solid. The product was used in the next step without further purification.

The crude product was suspended in 55 mL of hydrazine monohydrate. Stirring was maintained under reflux conditions for 5 hours. After allowing the mixture to cool to room temperature 100 mL of H_2O were added. The aqueous reaction mixture was extracted with CH_2Cl_2 (3×100 mL). The combined organic phases were dried over $MgSO_4$ and the solvent was removed under reduced pressure. 10.12 g of *N*-ethyloxindole were obtained as a yellow solid, which turns slowly red (62.80 mmol, 92% over two steps).

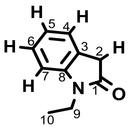

^{1}H-NMR ($CDCl_3$, 400 MHz) δ 7.25 – 7.20 (m, 2H, H-4, H-6), 7.00 (vt, J = 7.5 Hz, 1H, H-5), 6.81 (d, $^3J_{HH}$ = 7.8 Hz, 1H, H-7), 3.74 (q, $^3J_{HH}$ = 7.3 Hz, 2H, H-9), 3.48 (s, 2H, H-2), 1.30 (t, $^3J_{HH}$ = 7.3 Hz, 3H, H-10).

N,*N*',*N*''-Triethyltriazatruxene[11]

10.0 g of *N*-ethyloxindole (62.03 mmol, 1.0 eq.) were suspended in 70 mL of phosphorus oxychloride. The mixture was stirred under reflux conditions for 4 hours. After cooling to room temperature, the reaction mixture was poured onto 800 mL of crushed ice. The aqueous phase was extracted with CH_2Cl_2 (4×200 mL). The combined organic phases were dried over $MgSO_4$ and the solvent was removed under reduced pressure. The black crude product was purified by column chromatography using a 1:1 mixture of CH_2Cl_2 and petroleum ether as the eluent. 4.90 g of *N,N',N''*-triethyltriazatruxene were obtained as a slighty yellow-green powder (11.41 mmol, 56%).

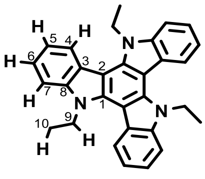

^{1}H-NMR ($CDCl_3$, 400 MHz) δ 8.36 (d, $^3J_{HH}$ = 8.1 Hz 3H, H-4), 7.67 (d, $^3J_{HH}$ = 8.1 Hz, 3H, H-7), 7.47 (vt, J = 7.6 Hz, 3H, H-6), 7.0 (vt, J = 7.6 Hz, 3H, H-5), 5.04 (q, $^3J_{HH}$ = 7.1 Hz, 2H, H-9), 1.62 (t, $^3J_{HH}$ = 7.1 Hz, 3H, H-10).

6-Bromo-N-dodecylisatin[13]

6-Bromoisatin (3.0 g, 13.27 mmol, 1.0 eq.) and K_2CO_3 (3.67 g, 26.54 mmol, 2.0 eq.) were suspended in 39 mL of *N*,*N*-dimethylformamide and 3.82 mL of dodecyl bromide (3.97 g, 15.92 mmol, 1.2 eq.) were added. The reaction mixture was heated to 80 °C overnight. After the reaction mixture was cooled to room temperature, 150 mL of H_2O were added. The mixture was extracted with CH_2Cl_2 (3×50 mL). The combined organic phases were washed with water (2×50 mL), dried over $MgSO_4$ and the solvents were removed under reduced pressure. The crude product was purified by column chromatography using ^{n}hexane : CH_2Cl_2 (1:1). 3.16 g of 6-bromo-*N*-dodecyl-isatin were obtained (8.02 mmol, 60% yield) as a red solid.

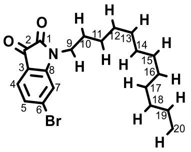

^{1}H-NMR ($CDCl_3$, 400 MHz) δ 8.01 (d, 1H, $^3J_{HH}$ = 8.6 Hz, H-5), 7.70 (d, 1H, $^4J_{HH}$ = 1.8 Hz, H-7), 7.41 (dd, 1H, $^3J_{HH}$ = 8.6 Hz, $^4J_{HH}$ = 1.8 Hz, H-5), 4.72 (t, 2H, $^3J_{HH}$ = 7.4 Hz, H-9), 1.9-1.81 (m, 2H, H-10), 1.34 – 1.09 (m, 18H, H-10 to H-19), 0.87 (t, 3H, $^3J_{HH}$ = 6.9 Hz, H-20).

6-Bromo-N-dodecyloxindole[14]

3.16 g of 6-bromo-*N*-dodecylisatin (8.0 mmol, 1.0 eq.) were dissolved in 20 mL of hydrazine hydrate and the reaction mixture was heated to reflux for 5 hours. After cooling to room temperature, 100 mL of H_2O were added. The aqueous phase was extracted with CH_2Cl_2 (3×50 mL). The combined organic phases were washed with 50 mL of H_2O and dried over $MgSO_4$. The solvents were removed under reduced pressure and the title compound was yielded as a yellow solid (2.56 g, 6.80 mmol, 85%).

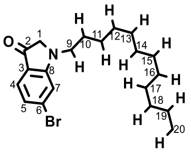

^{1}H-NMR ($CDCl_3$, 400 MHz) δ 7.16 (dd, 1H, $^3J_{HH}$ = 7.9 Hz, $^4J_{HH}$ = 1.7 Hz, H-5), 7.09 (d, 1H, $^3J_{HH}$ = 7.9 Hz, H-4), 6.95 (d, 1H, $^4J_{HH}$ = 1.7 Hz, H-7), 3.65 (t, $^3J_{HH}$ = 7.5 Hz, 2H), 3.45 (s, 2H, H-2), 1.72 - 1.60 (m, 2H, H-9), 1.40 – 1.21 (m, 18H, H-10 to H-19), 0.88 (t, $^3J_{HH}$ = 6.7 Hz, 3H, H-20).

2,2',2"-Tribromo-N,N',N"-tridodecyltriazatruxene (**2-Br$_3$-^{Dode}TAT**)

2.56 g of 6-bromo-*N*-dodecyloxindole (6.80 mmol, 1.0 eq.) were dissolved in 15 mL of $POCl_3$ and heated to reflux for 5 hours. After allowing to cool to room temperature, the reaction mixture was poured onto 100 g of crushed ice, stirred for 1 h and extracted with CH_2Cl_2 (3×60 mL). The combined organic phases were dried over $MgSO_4$ and the solvent was removed under reduced pressure. The crude product was purified by column chromatography using petrol ether : CH_2Cl_2 (4:1) as the eluent. 0.75 g of the product were isolated as a yellow powder (2.08 mmol, 30.6%).

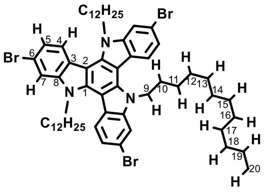

^{1}H-NMR ($CDCl_3$, 400 MHz) δ 7.46 (d, 3H, $^{3}J_{HH}$ = 7.9 Hz, H-4), 7.27 (dd, 3H, $^{3}J_{HH}$ = 7.9 Hz, $^{4}J_{HH}$ = 1.5 Hz, H-5), 7.06 (d, 3H, $^{4}J_{HH}$ = 1.5 Hz, H-7), 3.69 (t, 6H, $^{3}J_{HH}$ = 7.4 Hz, H-9), 1.68 (vpent, 6H, J = 7.5 Hz, H-10), 1.40 – 1.20 (m, 54H, H-11 to H-19), 0.88 (t, 9H, $^{3}J_{HH}$ = 6.8 Hz, H-20).

2,2',2"-Triferrocenyl-N,N',N"-tridodecyltriazatruxene (**2-Fc$_3$-^{NDode}TAT**)

100 mg of **2-Br$_3$-^{Dode}TAT** (0.01 mmol, 1.0 eq.), 148 mg of ferrocene boronic acid (0.64 mmol, 7.0 eq.), 78 mg of K_3PO_4 (0.37 mmol, 4.0 eq.) and 15 mg of Pd(dppf)Cl_2 were placed in a Schlenk tube. 10 mL of dry toluene and 0.8 mL of degassed H_2O were added. The reaction mixture was stirred under reflux conditions for 1 week. After cooling to room temperature, the solvents were removed under reduced pressure. The crude product was taken up in ^{n}pentane and filtered through a plug of Celite. The solvents were removed *in vacuo* and the residue was cooled to - 78 °C and washed with cold ^{n}pentane to afford 2,2',2"-triferrocenyl-*N,N',N"*-tridodecyltriazatruxene as a dark red, oily solid (45 mg, 0.032 mmol, 35%).

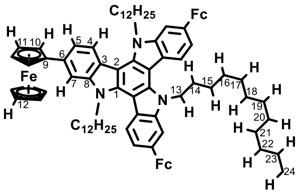

^{1}H-NMR (THF-d8, 400 MHz) δ 8.24 (d, 3H, $^{3}J_{HH}$ = 8.4 Hz, H-4), 7.84 (d, $^{4}J_{HH}$ = 1.5 Hz, 3H, H-7), 7.53 (dd, $^{3}J_{HH}$ = 8.4 Hz, $^{4}J_{HH}$ = 1.5 Hz, 3H, H-5), 5.05 (t, $^{3}J_{HH}$ = 7.7 Hz, 6H, H-13), 4.88, 4.36 (each vt, $^{3}J_{HH}$ = 1.9 Hz, 6H, H-10/11), 4.07 (s, 15H, H-12), 1.97 (vpent, $^{3}J_{HH}$ = 7.3 Hz, 6H, H-14), 1.17 – 0.48 (m, 48H, H-15 to H-22), 0.81-0.37 (m, 15H, H-23 and H-24).

^{13}C{^{1}H} NMR (THF-d8, 101 MHz) δ 142.8 (s, C-8), 139.7 (s, C-1), 135.0 (s, C-3), 123.0 (s, C-9), 122.2 (s, C-4), 119.5 (s, C-5), 108.9 (s, C-7) 104.8 (s, C-2), 87.8 (s, C6), 70.4 (s, 15C, C-12), 69.5 and 68.6 (s, 12C, C-10, C-11), 47.6 (s, C-13), 35.1, 32.9, 30.8 to 30.1, 23.6 (each s, C-15 to C-23), 30.3 (s, C-14), 14.5 (s, C-24).

2-(3-Hydroxy-3,3-dimethylprop-1-yn-1-yl)-N,N',N"-triethyltriazatruxene (**2-MebA$_1$-^{Et}TAT**) and *2,2'-bis(3-hydroxy-3,3-dimethylprop-1-yn-1-yl)-N,N',N"-triethyltriazatruxene* (**2-MebA$_2$-^{Et}TAT**)

1.12 g of *N,N',N"*-triethyltriazatruxene (2.61 mmol, 1.0 eq.) were dissolved in 300 mL of dry CH_2Cl_2 and cooled to 0 °C. A solution of 0.55 g of *N*-bromosuccinimide (3.65 mmol, 1.5 eq.) in 20 mL of dry DMF was added over 10 minutes. The reaction was stirred at 0 °C for 2 hours. Then, 200 mL of H_2O were added. The phases were separated and the aqueous phase was extracted with CH_2Cl_2 (3×100 mL). The combined organic phases were dried over $MgSO_4$ and the solvents were removed to yield a mixture of mono- and dibrominated **^{Et}TAT** as a greenish solid.

The greenish solid was used in the following Sonogashira coupling without purification. The crude solid and CuI (0.10 g, 0.53 mmol, 0.18 eq.) were dissolved in 120 mL of dry THF and 50 mL of Et_3N, 0.53 mL of 2-methylbut-3-yn-1-ol (0.46 g, 5.22 mmol, 2.1 eq.) and 0.12 g of Pd(dppf)Cl_2 were added. The reaction mixture was stirred for 96 h at 60 °C. After cooling to room temperature, 150 mL of H_2O were added. The phases were separated and the aqueous

phase was extracted with CH_2Cl_2 (3×70 mL). The combined organic phases were dried over $MgSO_4$ and the solvents were removed under reduced pressure. The residue was purified *via* column chromatography using a mixture of ethyl acetate : petroleum ether (2:8 to 4:6) as the eluent. 0.73 g of yellowish 2-(3-hydroxy-3,3-dimethylprop-1-yn-1-yl)-*N,N',N"*-triethyltriazatruxene (1.43 mmol, 55%) and 0.46 g of pale brown 2,2'-bis(3-hydroxy-3,3-dimethylprop-1-yn-1-yl)-*N,N',N"*-triethyltriazatruxene (0.78 mmol, 30%) were obtained as a pale brown solid.

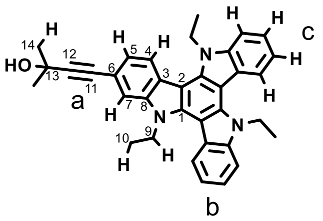

2-MebA$_1$-^{Et}TAT: ^{1}H-NMR ($CDCl_3$, 800 MHz) δ 8.33 (dd, $^3J_{HH}$ = 8.1 Hz, $^4J_{HH}$ = 3.7 Hz, 2H, H-4$_{b+c}$), 8.24 (d, $^3J_{HH}$ = 8.1 Hz, 1H, H-4$_a$), 7.72 (br, 1H, H-7$_a$), 7.67 (d, $^3J_{HH}$ = 8.1 Hz, 2H, H-7$_{b+c}$), 7.47 (vt, $^3J_{HH}$ = 7.6 Hz, 2H, H-6$_{b+c}$), 7.41 (d, $^3J_{HH}$ = 8.3 Hz, 1H, H-5$_a$), 7.36 (vt, $^3J_{HH}$ = 7.6 Hz, 2H, H-5$_{b+c}$), 5.01 (m, 6H, H-9), 2.10 (s, 1H, -OH), 1.71 (s, 6H, H-14$_a$), 1.66 – 1.56 (m, 9H, H-10).

^{13}C{^{1}H} NMR ($CDCl_3$, 202 MHz) δ 140.9 (s, C-8$_{b,c}$), 140.4 (s, C-8$_a$), 139.5 (s, C-1$_b$), 138.9 (s, C-1$_a$), 138.4 (s, C-1$_c$), 123.7 (s, C-6$_a$), 123.6 (s, C-5$_a$), 123.6 (s, C-3$_{b+c}$), 123.2 (s, C-6$_{b+c}$), 121.7 (vd, C-4$_{b,c}$), 121.3 (s, C-4$_a$), 120.1 (vd, C-5$_{b,c}$), 116.6 (s, C-3$_a$), 113.6 (s, C-7$_a$), 110.6 (vd, C-7$_{b,c}$), 103.6 (s, C-2$_a$), 103.3 (vd, C-2$_{b,c}$), 93.2 (s, C-12$_a$), 83.6 (s, C-11$_a$), 66.0 (s, C-13$_a$), 41.9 (m, C-9), 31.6 (s, C-14$_a$), 15.8 (s, 10$_a$), 15.6 (vd, 10$_{b,c}$).

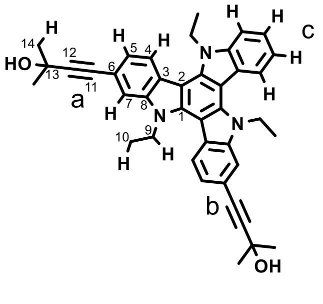

2-MebA$_2$-^{Et}TAT: ^{1}H-NMR ($CDCl_3$, 600 MHz) δ 8.32 (d, $^3J_{HH}$ = 8.1 Hz, 1H, H-4$_c$), 8.23-8.20 (m, 2H, H-4$_a$,4$_b$), 7.72 (s, 1H, H-7$_{a+b}$), 7.66 (vd, J = 8.0 Hz, 4.7 Hz, 1H, H-7$_c$), 7.49 - 7.45 (m, 1H, H-6$_c$), 7.42-7.39 (m, 2H, H-5$_{a+b}$), 7.38-7.34 (m, 1H, H-5$_c$), 5.14-4.85 (m, 6H, H-9), 2.11 (s, 2H, -OH), 1.72 (s, 12H, H-14), 1.65-1.56 (m, 9H, H-10).

^{13}C{^{1}H} NMR ($CDCl_3$, 152 MHz) δ 140.8 (s, C-8$_c$), 140.5, 140.4 (s, C-8$_{a,b}$), 139.7, 139.2, 138.8 (each s, C-1$_{a,b,c}$), 123.7 (m, C-5$_{a,b}$), 123.5 (s, C-6$_{a+b}$), 123.3 (s, C-6$_c$), 121.7 (s, C-6$_c$), 121.3 (s, C-4$_{a,b}$), 120.2 (s, C-5$_c$), 116.8 (s, C-3$_{a+b}$), 113.6 (vd, C-7$_{a,b}$), 110.6 (s, C-7$_c$), 103.5 (s, C-2$_{a,b}$), 103.2 (s, C-2$_c$), 93.2 (s, C-12$_{a+b}$), 83.4 (s, C-11$_{a+b}$), 66.0 (s, C-13$_{a+b}$), 41.9 (s, C-9$_{a,b,c}$), 31.8 (s, C-14$_{a,b}$), 15.6 (s, C-10$_{a,b}$), 15.5 (s, C-10$_c$).

2-Ethynyl-N,N',N"-triethyltriazatruxene (**2-A$_1$-^{Et}TAT**)

0.22 g of **2-MebA$_1$-^{Et}TAT** (0.43 mmol, 1.0 eq.) were placed in a three-necked round bottom flask. 160 mL of toluene, 6 mL of methanol, 1.6 mL of H_2O and 0.5 g of KOH were added and the reaction vessel was equipped with an air cooler. The solution was stirred overnight at 80 °C while purging with nitrogen gas. After cooling to room temperature, 200 mL of H_2O were

added and the phases were separated. The aqueous phase was extracted with CH_2Cl_2 (3×80 mL). The combined organic phases were dried over $MgSO_4$ and the solvents were removed to give 0.14 g of **2-A$_1$-^{Et}TAT** as a golden-yellowish solid (0.31 mmol, 72%).

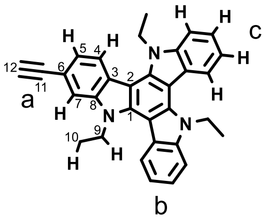

^{1}H-NMR ($CDCl_3$, 400 MHz) δ 8.34 (vdd, J = 8.4, 9.5 Hz, 2H, H-4$_{b,c}$), 8.25 (d, $^3J_{HH}$ = 8.4 Hz, 1H, H-4$_a$), 7.80 (s, 1H, H-7$_a$), 7.67 (d, $^3J_{HH}$ = 8.1 Hz, 2H, H-7$_{b,c}$), 7.51 – 7.44 (m, 3H, H-6$_{b,c}$, H-5$_a$) , 7.36 (vt, $^3J_{HH}$ = 7.6 Hz, 2H, H-5$_{b,c}$), 5.08 – 4.94 (m, 6H, H-9$_{a,b,c}$), 3.18 (s, 1H, H-12$_a$), 1.66-1.56 (m, 9H, H-10$_{a,b,c}$).

^{13}C{^{1}H} NMR ($CDCl_3$, 101 MHz) δ 140.9 (vd, C-8$_{b+c}$), 140.3 (s, C-8$_a$), 139.6, 139.2, 138.5 (each s, C-1$_{a,b,c}$), 124.1 (s, C-5$_a$), 124.0 (s, C-6$_a$), 123.6 (vd, C-3$_{b,c}$), 123.2 (s, C-5$_{b+c}$), 121.7 (vd, C-4$_{b,c}$), 121.3 (s, C-4$_a$), 120.2 (s, C-6$_{b,c}$), 115.8 (s, C-3$_a$), 114.2 (s, C-7$_a$), 110.6 (s, C-7$_{b,c}$), 103.6 (s, C-2$_a$), 103.3 (s, 2C, C-2$_{b,c}$), 85.3 (s, C-11$_a$), 76.5 (s, C-12$_a$), 41.9 (s, C-9$_{a+b+c}$), 15.6 (vd, C-10$_{b,c}$), 15.6 (s, C-10$_a$).

Iodoferrocene[15]

3.00 g of ferrocene (16.13 mmol, 1.0 eq.) and 0.18 g of ^{t}BuOK (1.61 mmol, 0.10 eq.) were dissolved in 150 mL of dry THF. The reaction mixture was cooled to -78 °C. 21.22 mL of a 1.9 M solution of ^{t}BuLi in ^{n}pentane (40.31 mmol, 2.5 eq.) were added dropwise and the mixture was left stirring at -78 °C for 2 h. 10.23 g of iodine (40.32 mmol, 2.5 eq.) were added. The reaction mixture was stirred for 30 minutes while allowing to warm to room temperature. The organic phase was washed with a saturated solution of $Na_2S_2O_3$ (50 mL). The solvent was removed under reduced pressure, yielding a black oil. The crude product was dissolved in ^{n}hexane and filtered through a plug of silica with ^{n}hexane as the eluent. The organic phase was washed with a saturated, aqueous solution of iron tricloride until the aqueous phase did not show a blue-green colour anymore. The organic phase was dried with Na_2SO_4 and the solvent was removed under reduced pressure. 2.45 g of iodoferrocene were obtained as red-brown crystals (7.85 mmol, 49%).

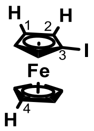

^{1}H-NMR ($CDCl_3$, 500 MHz) δ 7.42 (s, 2H, H-4), 4.43 (vt, J_{HH} = 1.78 Hz, 2H, H-1), 4.28 (vt, J_{HH} = 1.78 Hz, 2H, H-2), 4.06 (s, 5H, H-4).

Ferrocene boronic acid[16]

1.50 g of ferrocene (8.06 mmol, 1.0 eq.) and 0.14 g of ^{t}BuOK (1.21 mmol, 0.15 eq.) were dissolved in 50 mL of dry THF. The reaction mixture was cooled to -78 °C and 10.61 mL of a 1.9 M solution of ^{t}BuLi in pentane (20.16 mmol, 2.5 eq.) were added dropwise. Stirring at -78 °C was continued for 2 h. 4.65 mL of triisopropyl borate (20.16 mmol, 2.5 eq.) were added dropwise at -78 °C. The reaction mixture was allowed to warm to room temperature and was

stirred overnight. The solvent was removed under reduced pressure and the residue was dissolved in 50 mL of ethyl acetate. The organic phase was extracted with a solution of Na_2CO_3 and sorbitol (1M, 6×80 mL). The aqueous phase was washed with 80 mL of a mixture of diethyl ether and pentane (1:1). The aqueous phase was cooled to 0 °C and 250 mL of diethyl ether were added. The mixture was vigorously stirred and acidified with concentrated hydrochloric acid to pH 1. The phases were separated and the aqueous phase was washed with water and brine (80 mL each). The combined organic phases were dried over $MgSO_4$ and the solvent was removed under reduced pressure. 1.02 g of ferrocene boronic acid were obtained as an orange solid (4.45 mmol, 55%).

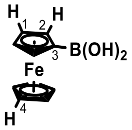

^{1}H-NMR (DMSO-D6, 400 MHz) δ 7.41 (s, 2H, OH), 4.433 (vt, J_{HH} = 2.0 Hz, 2H, H-1), 4.431 (s, 5H, H-3), 4.17 (vt, J_{HH} = 2.0 Hz, 2H, H-2).

2,2'-Diethynyl-N,N',N''-triethyltriazatruxene (**2-A$_2$-^{Et}TAT**)

0.39 g of **2-MebA$_2$-^{Et}TAT** (0.66 mmol, 1.0 eq.) were placed in a three-necked round bottom flask. 200 mL of toluene, 10 mL of methanol, 2 mL of H_2O and 0.7 g of KOH were added. The reaction vessel was equipped with an air cooler. The solution was stirred for 30 h at 80 °C while purging with nitrogen gas. After cooling to room temperature, 200 mL of H_2O were added and the phases were separated. The aqueous phase was extracted with CH_2Cl_2 (3×80 mL). The combined organic phases were dried over $MgSO_4$ and the solvents were removed to give 0.30 g of **2-A$_2$-^{Et}TAT** as golden-yellow solid (0.63 mmol, 96%).

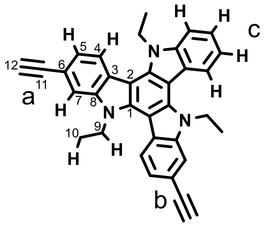

^{1}H-NMR (CDCl$_3$, 400 MHz) δ 8.32 (d, $^3J_{HH}$ = 8.1 Hz, 1H, H-4$_c$), 8.24 (vdd, J_{HH} = 8.3 Hz, 5.3 Hz, 2H, H-4$_{a,b}$), 7.80 (s, 2H, H-7$_{a,b}$), 7.67 (d, $^3J_{HH}$ = 8.1 Hz, 1H, H-7$_c$), 7.51 - 7.45 (m, 3H, H-5$_{a,b}$,H-6$_c$), 7.37 (vt, J_{HH} = 8.0 Hz, 1H, H-5$_c$), 5.05-4.92 (m, 6H, H-9$_{a,b,c}$), 3.19 (s, 2H, H-12$_{a,b}$), 1.67-1.56 (m, 9H, 10$_{a,b,c}$).

^{13}C{^{1}H} NMR (CDCl$_3$, 101 MHz) δ 140.9 (s, C-8$_c$), 140.3 (2s, C-8$_{a,b}$), 140.0, 139.4, 138.9 (each s, C-1$_{a,b,c}$), 124.1 (2s, C-5$_{a,b}$), 124.0 (2s, C-6$_{a,b}$), 123.4 (2s, C-5$_c$,C-3$_c$), 121.7 (s, C-4$_c$), 121.4 (2s, C-4$_{a,b}$), 120.3 (s, 1C, C-6$_c$), 116.1 (s, C-3$_{a,b}$), 114.2 (2s, C-7$_{a,b}$), 110.6 (s, C-7$_c$), 103.5 (s, C-2$_{a,b}$), 103.2 (s, C-2$_c$), 85.1 (s, C-11$_{a,b}$), 76.6 (s, C-12$_{a,b}$), 41.91 (s, C-9$_{a,b,c}$), 15.7 (2s, C-10$_{a,b}$), 15.6 (s, C-10$_c$).

2,2',2''-Tri(ferrocenylethynyl)-N,N',N''-triethyltriazatruxene (**2-(Fc-A)$_3$-^{Et}TAT**)

3,3',3''-Tribromo-*N,N',N''*-triethyltriazatruxene (80 mg, 0.12 mmol, 1.0 eq.) and ethynylferrocene (0.11 g, 0.53 mmol, 4.4 eq.) were dissolved in 5 mL of dry toluene and 1 mL of dry Et_3N. 11 mg of CuI (0.06 mmol, 0.5 eq.) and 5.9 mg of Pd(dppf)Cl_2 were added. The reaction mixture was stirred for 4 days at 100 °C. After cooling to room temperature, the

reaction mixture was filtered through a plug of Celite using CH_2Cl_2 as the eluent. The solvents were removed under reduced pressure. The residue was washed with diethyl ether (7×10 mL) and with 5 mL of methanol. 2,2',2''-Tris-tri(ferrocenyl ethynyl)-*N,N',N''*-triethyltriazatruxene was obtained as a dark brown powder (61 mg, 0.06 mmol, 48%).

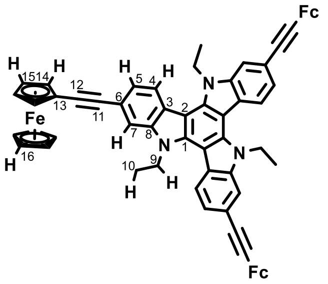

^{1}H-NMR ($CDCl_3$, 400 MHz) δ 8.25 (d, $^3J_{HH}$ = 8.39 Hz, 3H, H-4), 7.78 (d, $^4J_{HH}$ = 1.44 Hz, 3H, H-7), 7.50 (dd, $^3J_{HH}$ = 8.39 Hz, $^4J_{HH}$ =1.44 Hz, 3H, H-5), 5.06 (q, $^3J_{HH}$ = 7.1 Hz, 6H, H-9), 4.83 Hz (vt, J_{HH} = 1.9 Hz, 6H, H-14), 4.31 (s, 15H, H-16), 4.28 (vt, J_{HH} = 1.9 Hz, 6H, H-15), 1.64 (t, $^3J_{HH}$ = 7.1 Hz, 9H, H-10).

^{13}C{^{1}H} NMR ($CDCl_3$, 101 MHz) δ 140.9 (s, C-8), 139.7 (s, C-1), 123.9 (s, C-5), 123.2 (s, C-6), 121.8 (s, C-4), 118.6 (s, C-3), 113.5 (s, C-7), 103.8 (s, C-2), 88.1 (s, C-12) 87.1 (s, C-11), 71.3 (s, C-14/15), 70.4 (s, C-16), 69.3 (s, C-14/15), 42.3 (s, C-9), 15.7 (s, C-10).

3,3',3''-Tri(ferrocenylethynyl)-N,N',N''-triethyltriazatruxene (**3-(Fc-A)$_3$-^{Et}TAT**)

2,2',2''-Tribromo-*N,N',N''*-triethyltriazatruxene (0.100 g, 0.15 mmol, 1.0 eq.), ethynylferrocene (0.13 g, 0.66 mmol, 4.4 eq.) were dissolved in 5 mL of dry toluene and 1 mL of dry Et_3N. 11 mg of CuI (0.06 mmol, 0.4 eq.) and 10 mg of Pd(dppf)Cl_2 were added. The reaction mixture was stirred for 7 days at 100 °C. After cooling to room temperature, the reaction mixture was filtered thought a plug of Celite using CH_2Cl_2 as eluent. The solvents were removed under reduced pressure. The residue was washed with diethyl ether (7×10 mL) and once with methanol (5 mL). 3,3',3''-Tri(ferrocenylethynyl)-*N,N',N''*-triethyltriazatruxene was obtained as dark orange powder (0.12 g, 0.14 mmol, 76%).

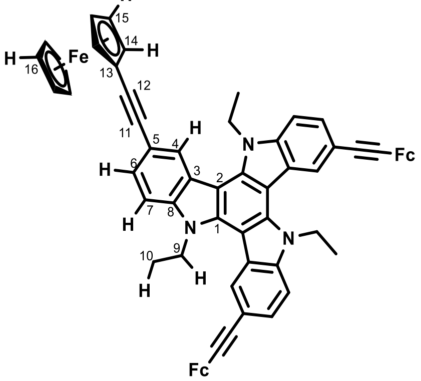

^{1}H-NMR ($CDCl_3$, 400 MHz) δ 8.25 (d, $^3J_{HH}$ = 8.39 Hz, 3H, H-4), 7.62 (m, 6H, H-6, H-7), 5.04 (q, $^3J_{HH}$ = 7.1 Hz, 6H, H-9), 4.59 (vt, J_{HH} = 1.9 Hz, 6H, H-14/15), 4.31 (s, 15H, H-16), 4.28 (vt, J_{HH} = 1.9 Hz, 6H, H-14/15), 1.67 (t, $^3J_{HH}$ = 7.1 Hz, 9H, H-10).

$^{13}C\{^1H\}$ NMR ($CDCl_3$, 101 MHz) δ 140.4 (s, C-8), 139.2 (s, C-1), 126.9 (s, C-6), 125.2 (s, C-4), 123.4 (s, C-5), 115.3 (s, C-3), 110.5 (s, C-7), 103.3 (s, C-2), 87.3 (s, C-11), 86.1 (s, C-12), 71.5 (s, C-14/15), 70.2 (s, C-16), 68.8 (s, C-14/15), 66.2 (s, C-13), 42.1 (s, C-9), 15.6 (s, C-10).

2-Ferrocenylethynyl-N,N',N"-triethyltriazatruxene (**2-(Fc-A)$_1$-^{Et}TAT**)

100 mg of **2-A$_1$-^{Et}TAT** (0.22 mmol, 1.0 eq.) were dissolved in 15 mL of THF. The solution was cooled to -78 °C and 0.1 mL of a 2.5 M solution of ^{n}BuLi in hexane (0.26 mmol, 1.2 eq.) were added dropwise. The solution was stirred for 1 h. 72.4 mg of $ZnCl_2$(tmeda) (0.29 mmol, 1.3 eq.) were dissolved in 10 mL of THF and added dropwise. The reaction mixture was allowed to warm to room temperature and stirring was continued for 90 min. Afterwards, 103 mg of iodoferrocene (0.33 mmol, 1.5 eq) and 25 mg of $Pd(PPh_3)_4$ (7 mol%) were added. The reaction mixture was heated to reflux for one week. The reaction mixture was allowed to cool to room temperature and filtered over Celite using CH_2Cl_2 as the eluent. The solvent was removed under reduced pressure. The remaining solid was extracted with diethyl ether (2×30 mL). After removing the solvent, the orange solid was further purified via column chromatography using CH_2Cl_2 : petroleum benzene (1:1) as the eluent. 41 mg of 2-ferrocenylethynyl-*N,N',N"*-triethyltriazatruxene were obtained as an orange solid (0.064 mmol, 29%).

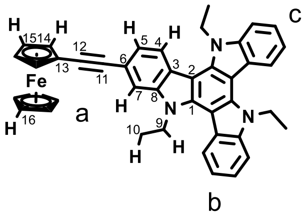

^{1}H-NMR ($CDCl_3$, 400 MHz) δ 8.36 (d, $^3J_{HH}$ = 8.4 Hz, 2H, H-4$_{b,c}$), 8.28 (d, $^3J_{HH}$ = 8.4 Hz, 1H, H-4$_a$), 7.80 (s, 1H, H-7$_a$), 7.70 (d, $^3J_{HH}$ = 7.3 Hz, 2H, H-7$_{b+c}$), 7.51 - 7.45 (m, 3H, H-5$_{a,b,c}$), 7.37 (vt, $^3J_{HH}$ = 7.3 Hz, 2H, H-6$_{b,c}$), 5.09-4.98 (m, 6H, H-9$_{a,b,c}$), 4.58 (vt, J_{HH} = 1.8 Hz, 2H, H-14), 4.33-4.27 (m, 7H, H-15,H-16), 1.66-1.56 (m, 9H, H-10$_{a,b,c}$).

$^{13}C\{^1H\}$ NMR ($CDCl_3$, 101 MHz) δ 141.9 (s, C-8$_{b,c}$), 140.9 (s, C-8$_a$), 139.7, 139.2, 138.7 (each s, C-1$_{a,b,c}$), 123.8 (2s, C-3$_{b,c}$), 123.5 (s, 3C, C-5$_{a,b,c}$), 122.0 (2s, C-4$_{b,c}$), 121.7 (s, C-4$_a$), 120.4 (s, 2C, C-6$_{b,c}$), 118.3 (s, C-3$_a$), 113.5 (s, C-7$_a$), 110.9 (vd, C-7$_{b,c}$), 103.8-103.4 (m, C-2$_{a,b,c}$), 94.1 (s, C-12$_a$), 87.3 (s, C-11$_a$) 71.8 (s, H-14$_a$), 70.4 (s, C-16), 69.3 (s, C-15), 66.2 (s, C-13), 42.2 (s, C-9$_{a+b+c}$), 15.8 (s, C-10$_a$), 15.6 (s, C-10$_{b,c}$).

2,2'-Di(ferrocenylethynyl)-N,N',N"-triethyltriazatruxene (**2-(Fc-A)$_2$-^{Et}TAT**)

100 mg of **2-A$_2$-^{Et}TAT** (0.21 mmol, 1.0 eq.) were dissolved in 15 mL of THF. The solution was cooled to -78 °C and 0.2 mL of a 2.5 M solution of ^{n}BuLi in ^{n}hexane (0.50 mmol, 2.4 eq.) were added dropwise. The resulting solution was stirred for 1 h. 0.14 mg of $ZnCl_2$(tmeda) (0.54 mmol, 2.6 eq.) were dissolved in 10 mL of THF and added dropwise to the solution. The reaction mixture was allowed to reach room temperature and the solution was stirred for further 90 min. 0.20 g of iodoferrocene (0.63 mmol, 3.0 eq.) and 25 mg of $Pd(PPh_3)_4$ (9.5 mol%) were added. The reaction mixture was heated to reflux for one week. After allowing to cool to room temperature, the reaction mixture was filtered over Celite using CH_2Cl_2 as the eluent. The solvent was removed under reduced pressure. The remaining solid was dissolved in 5 mL of CH_2Cl_2. 30 mL of ^{n}pentane were added. An orange-red solid precipitated and the supernatant was cannulated off. The solid was washed with 40 mL of pentane. The crude product was

purified via column chromatography using CH_2Cl_2 : petroleum benzene (1:1). 60 mg of the product were obtained as red solid (0.07 mmol, 34%).

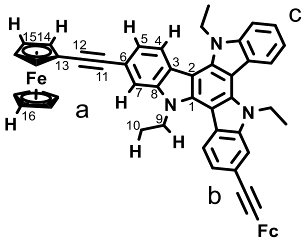

^{1}H-NMR ($CDCl_3$, 400 MHz) δ 8.36 (d, $^3J_{HH}$ = 8.1 Hz, 1H, H-4$_c$), 8.28 (d, $^3J_{HH}$ = 8.4 Hz, 2H, H-4$_{a,b}$), 7.81 (s, 2H, H-7$_{a,b}$), 7.70 (d, $^3J_{HH}$ = 8.1 Hz, 1H, H-7$_c$), 7.51-7.45 (m, 3H, H-5$_{a,b,c}$), 7.39 (vt, $^3J_{HH}$ = 7.5 Hz, 1H, H-6$_c$), 5.09-4.98 (m, 6H, H-9$_{a,b,c}$), 4.58 (vt, J_{HH} = 1.8 Hz, 4H, H-14), 4.31 (s, 14H, H-15, H-16), 1.66-1.61 (m, 6H, H-10$_{a,b}$), 1.61-157 (m, 3H, H-10).

^{13}C{^{1}H} NMR ($CDCl_3$, 101 MHz) δ 141.2 (s, C-8$_c$), 141.0 (s, C-8$_{a,b}$), 139.9, 139.4, 139.0 (each s, C-1$_{a,b,c}$), 123.8 (m, C-3$_c$, C-5$_c$), 123.6 (vd, C-5$_{a,b}$), 122.0 (s, C-4$_c$), 121.8 (vd, C-3$_{a,b}$), 120.5 (s, C-6$_c$), 118.4 (d, C-3$_{a,b}$), 113.5 (vd, C-7$_{a,b}$), 110.9 (s, C-7$_c$), ´103.8, 103.5, 103.6 (each s, C-2$_{a,b,c}$), 88.0 (s, C-12$_{a,b}$), 87.2 (s, C-11$_{a,b}$), 71.8 (s, C-14$_{a,b}$), 70.4 (s, C-16$_{a,b}$), 69.3 (s, C-15$_{a,b}$), 66.2 (s, C-13$_{a,b}$), 42.2 (s, C-9$_{a,b,c}$), 15.8 (s, C-10$_{a,b}$), 15.6 (s, C-10$_c$).

NMR and Mass Spectra

2-MebA$_1$-^{Et}TAT

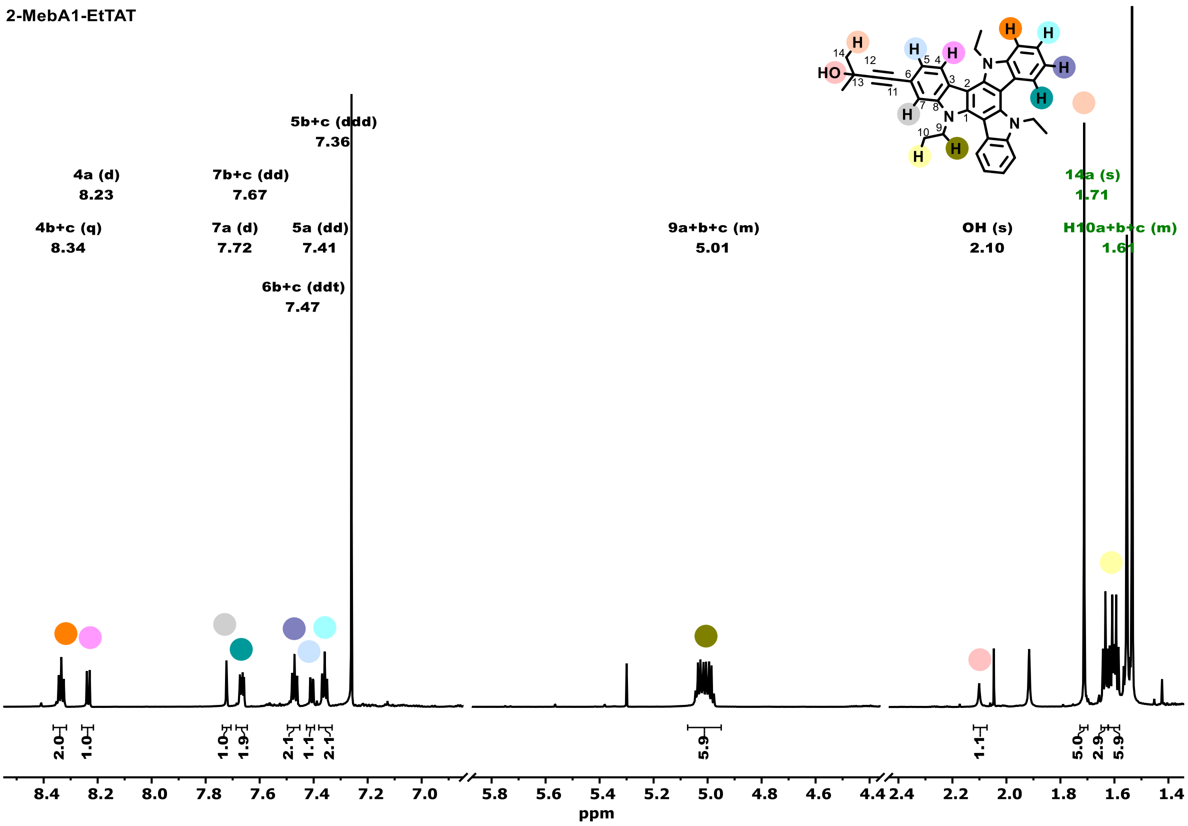

Figure S1. ^{1}H-NMR spectrum ($CDCl_3$, 800 MHz) of **2-MebA$_1$-^{Et}TAT**.

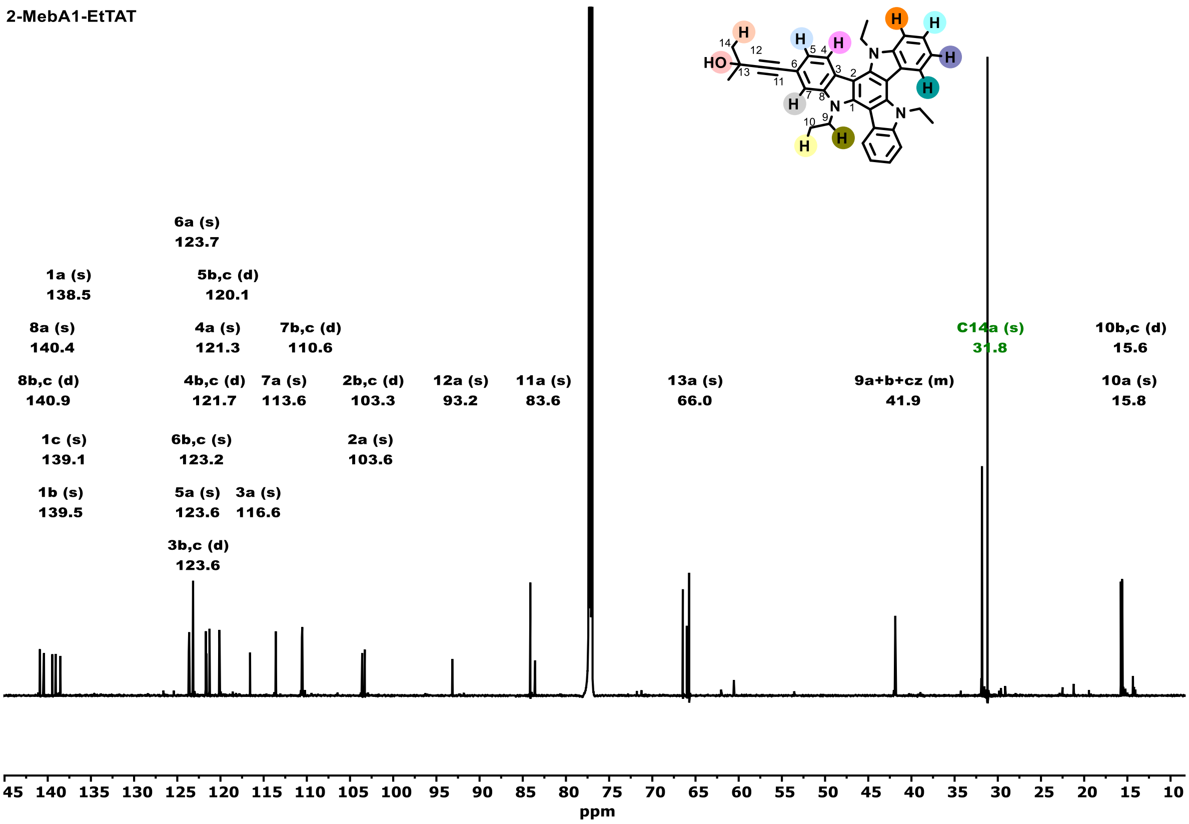

Figure S2. ^{13}C{1H}-NMR spectrum ($CDCl_3$, 202 MHz) of **2-MebA$_1$-^{Et}TAT**.

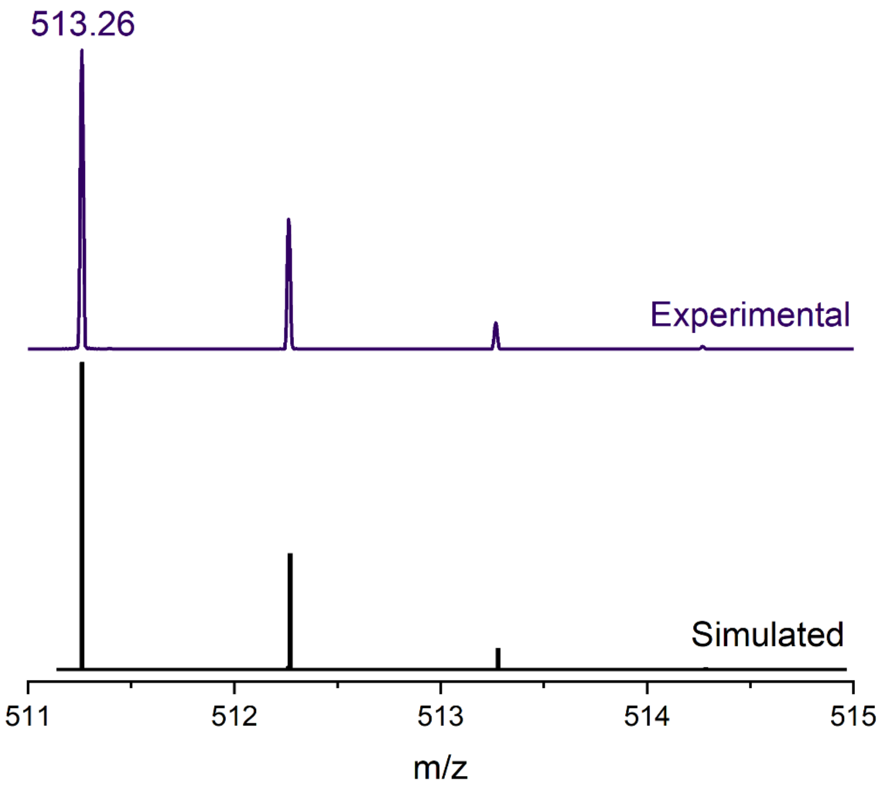

Figure S3. Molecular ion peak in the ESI mass spectrum (CH_2Cl_2) of **2-MebA$_1$-^{Et}TAT**.

2-MebA$_2$-^{Et}TAT

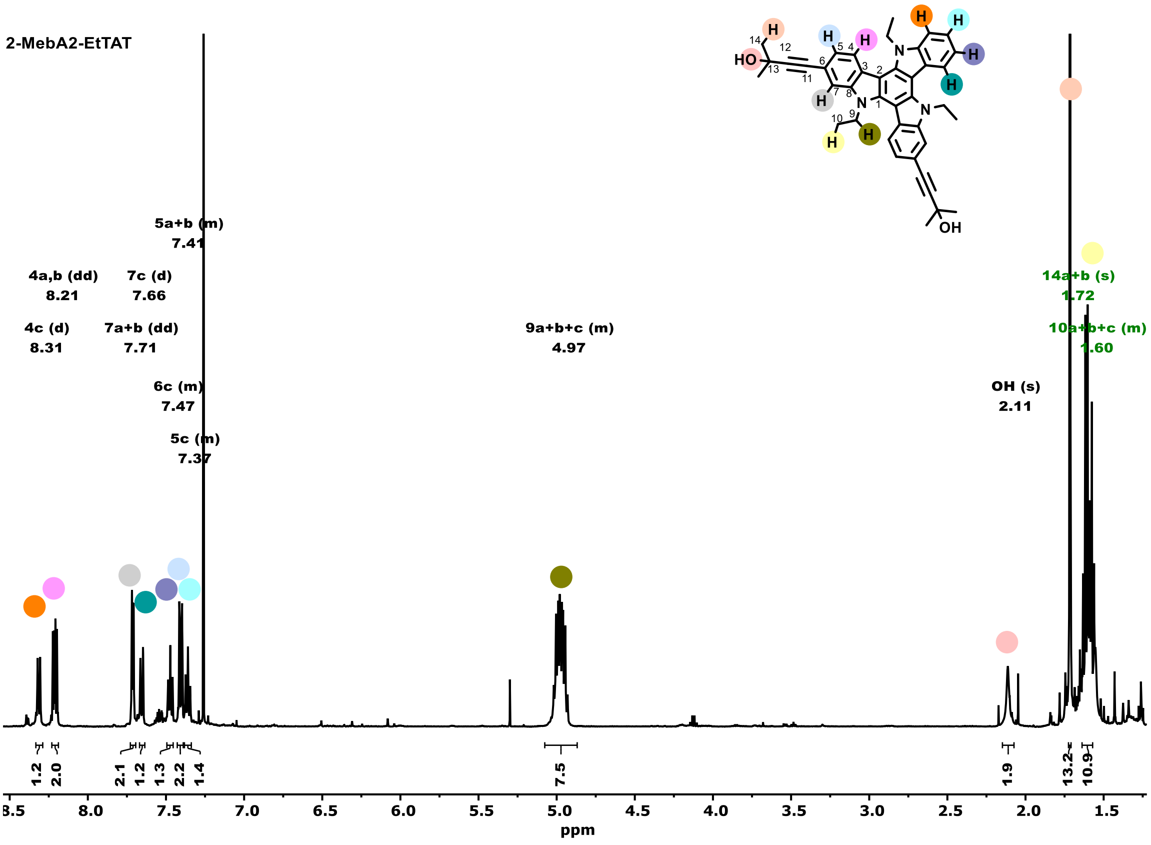

Figure S4. ^{1}H-NMR spectrum ($CDCl_3$, 800 MHz) of **2-MebA$_2$-^{Et}TAT**.

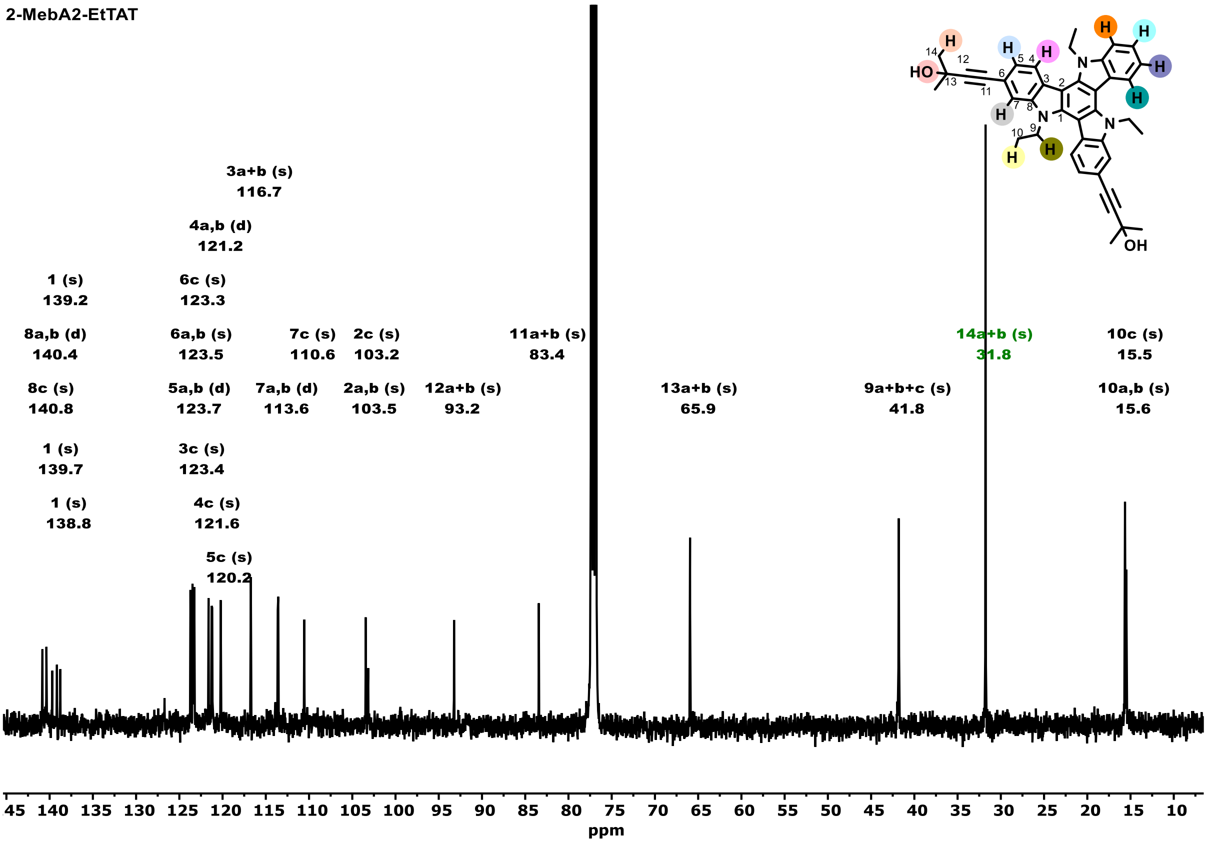

Figure S5. $^{13}C\{^1H\}$-NMR spectrum ($CDCl_3$, 152 MHz) of **2-MebA$_2$-^{Et}TAT**.

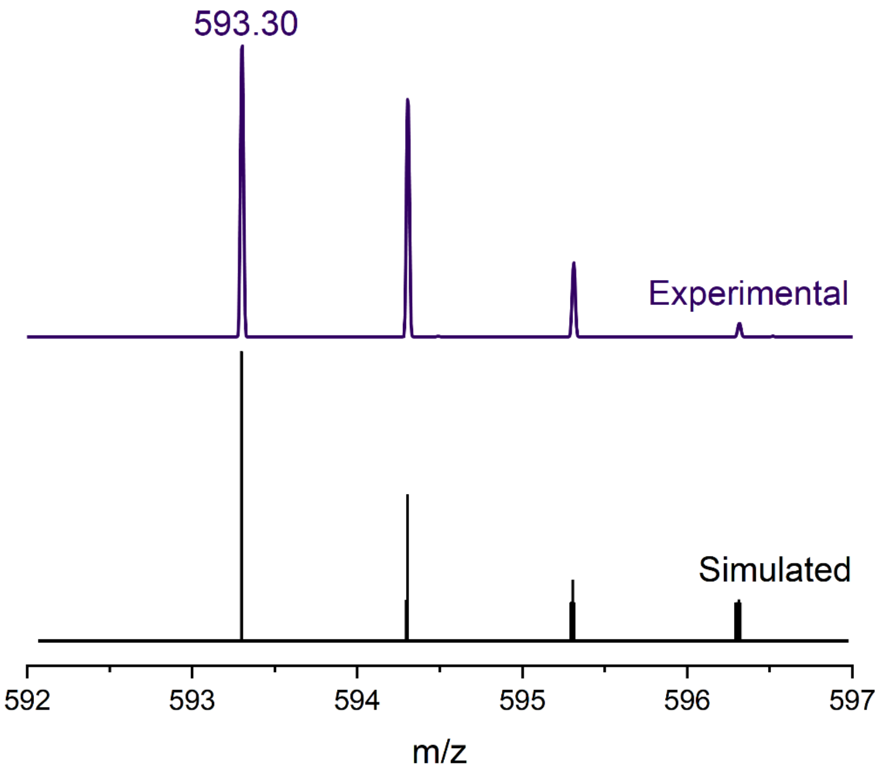

Figure S6. Molecular ion peak in the ESI mass spectrum (CH_2Cl_2) of **2-MebA$_2$-^{Et}TAT**.

2-A$_1$-^{Et}TAT

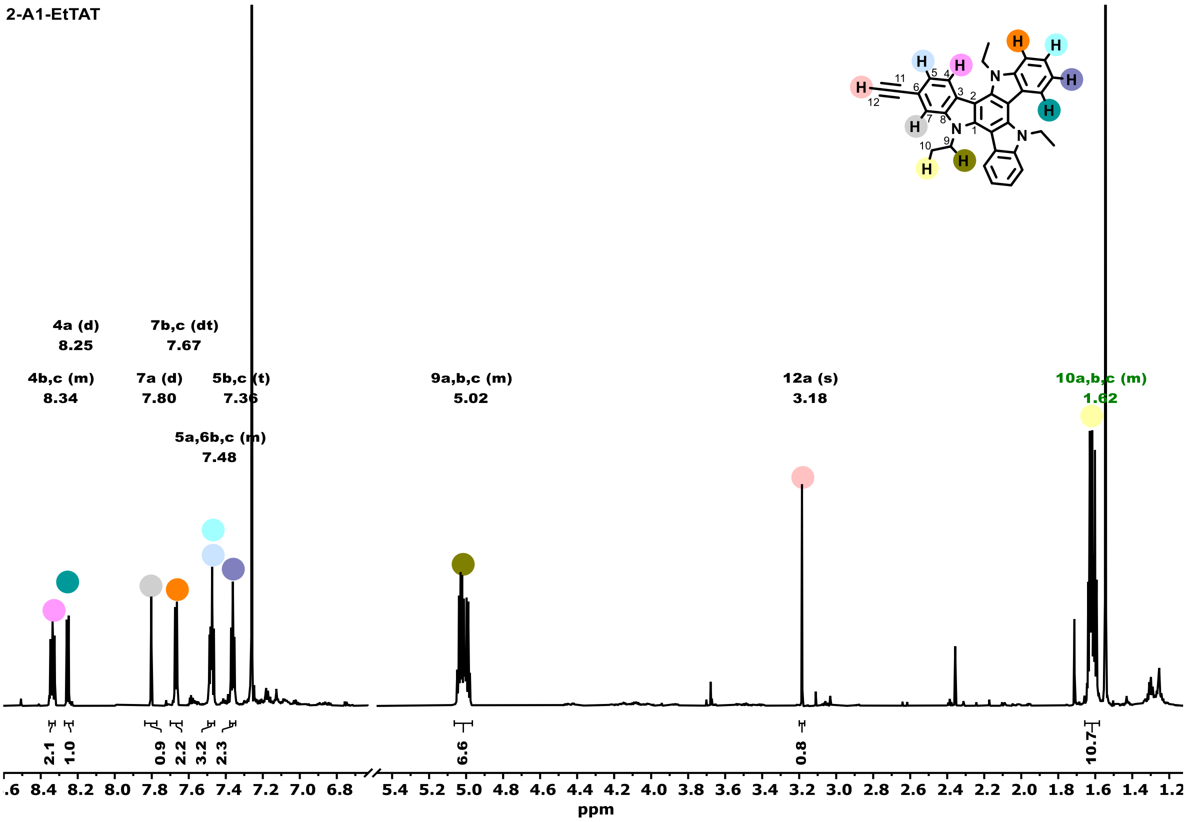

Figure S7. ^{1}H-NMR spectrum ($CDCl_3$, 400 MHz) of **2-A$_1$-^{Et}TAT.**

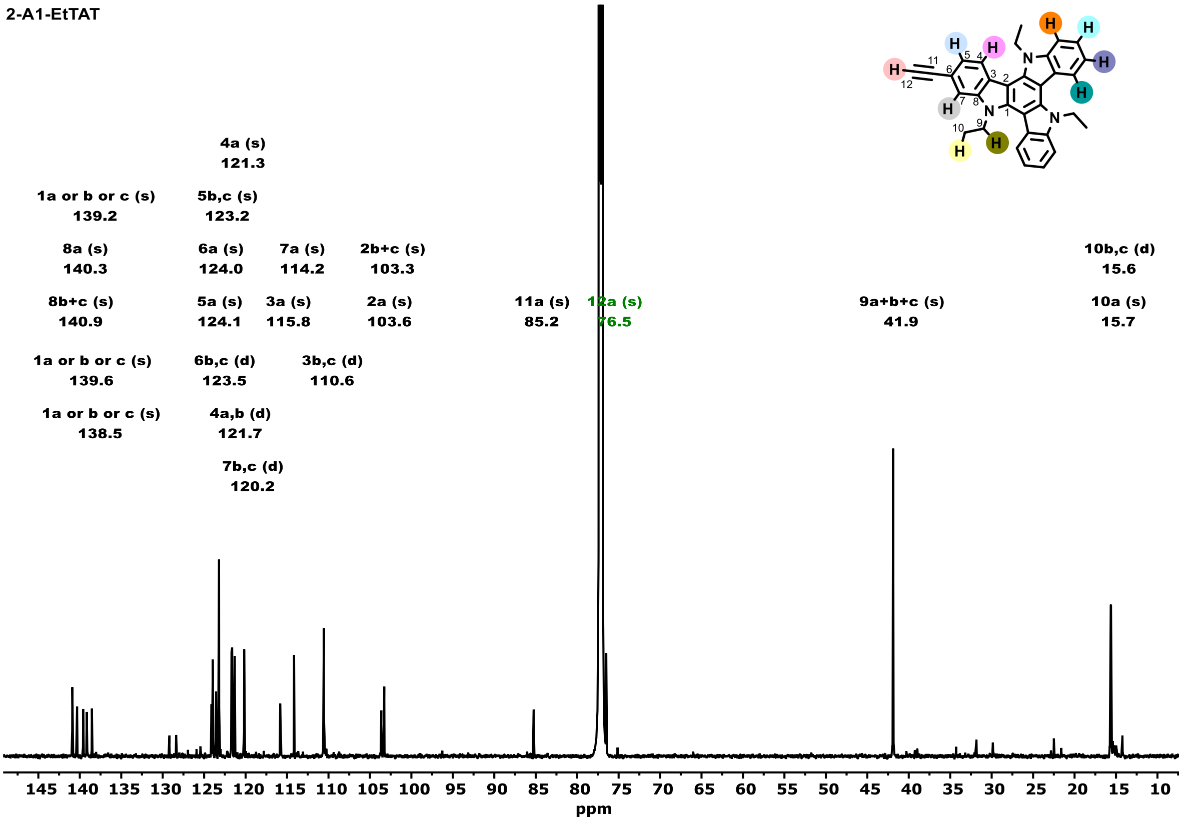

Figure S8. ^{13}C{^{1}H}-NMR spectrum ($CDCl_3$, 102 MHz) of **2-A$_1$-^{Et}TAT**.

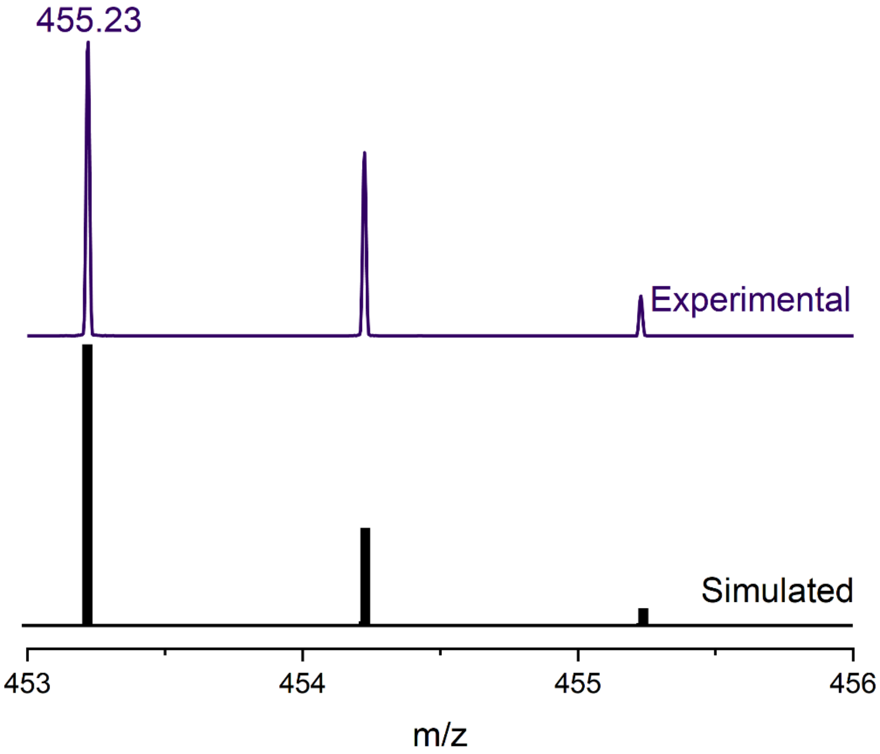

Figure S9. Molecular ion peak in the ESI mass spectrum (CH_2Cl_2) of **2-A$_1$-^{Et}TAT**.

2-A$_2$-^{Et}TAT

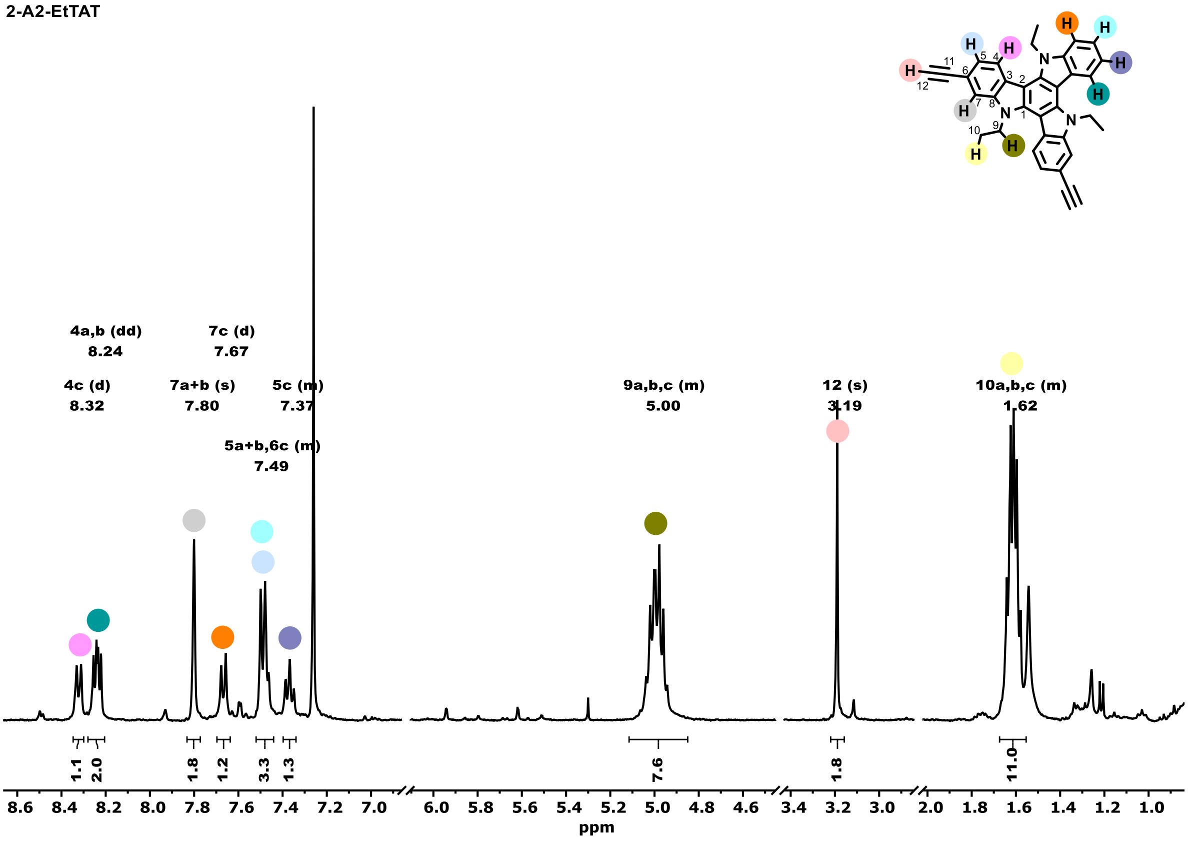

Figure S10. ^{1}H-NMR spectrum ($CDCl_3$, 400 MHz) of **2-A$_2$-^{Et}TAT**.

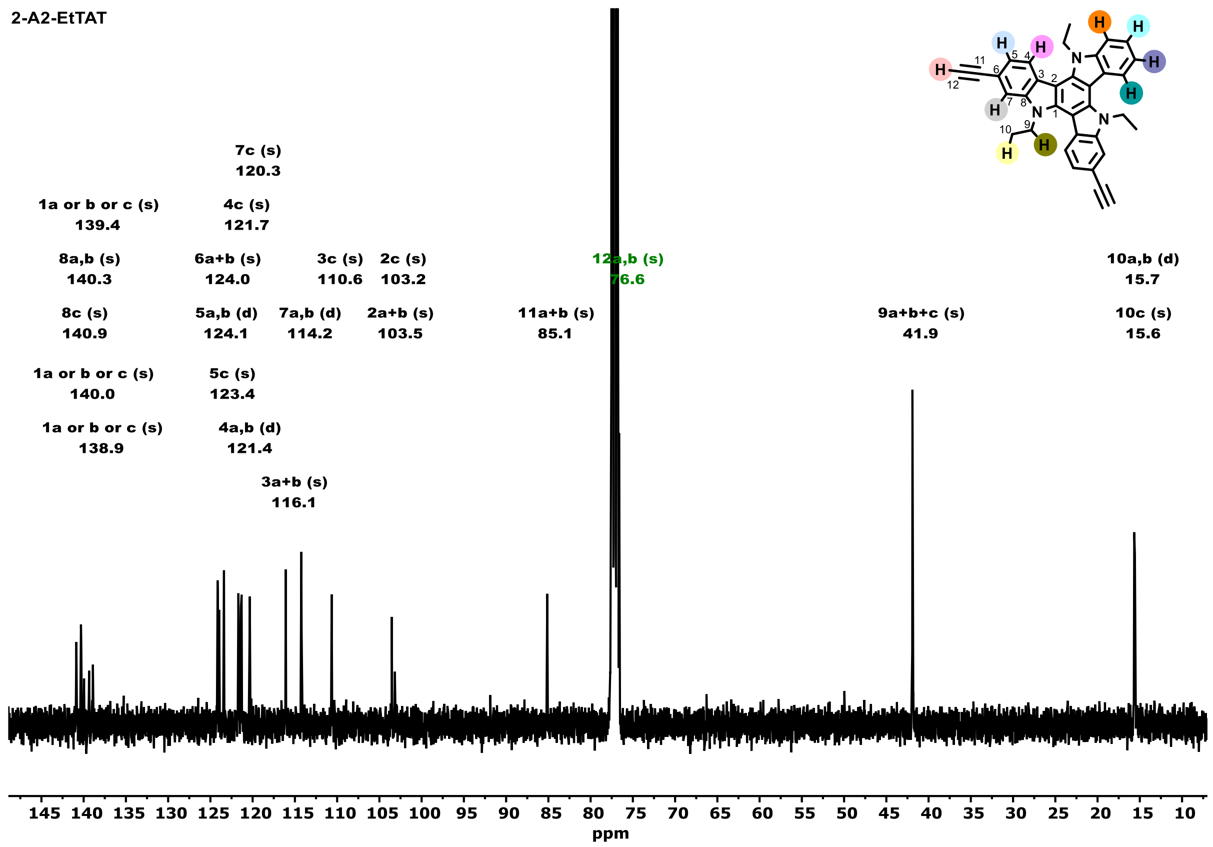

Figure S11. $^{13}C\{^1H\}$-NMR spectrum ($CDCl_3$, 102 MHz) of **2-A$_2$-^{Et}TAT.**

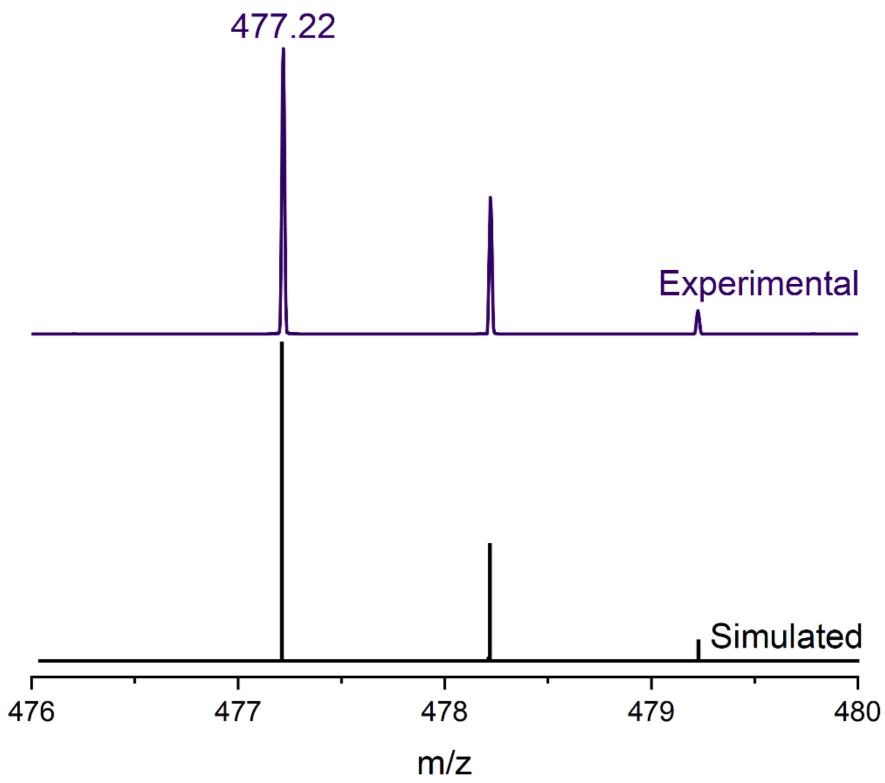

Figure S12. Molecular ion peak in the ESI mass spectrum (CH_2Cl_2) of **2-A$_2$-^{Et}TAT**.

2-Fc$_3$-^{Et}TAT

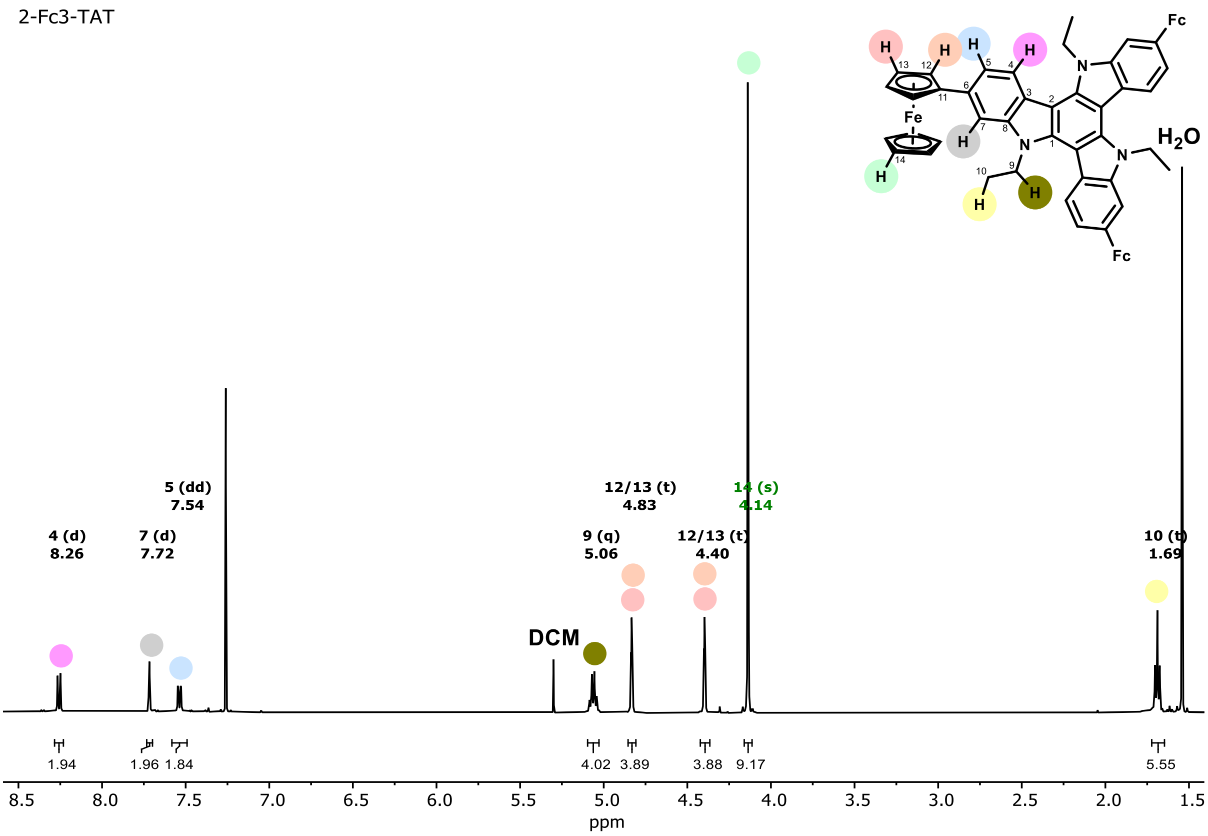

Figure S13. ^{1}H-NMR spectrum (CDCl$_3$, 400 MHz) of **2-Fc$_3$-^{Et}TAT.**

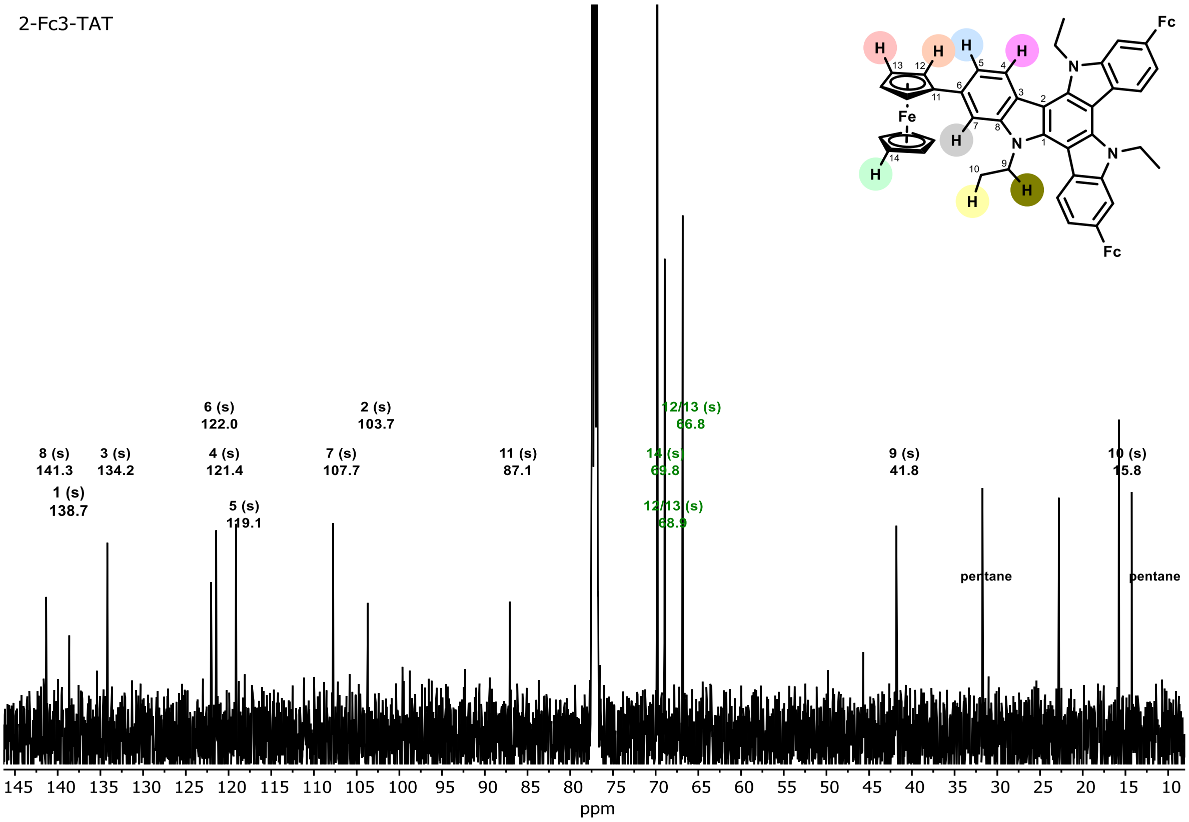

Figure S14. ^{13}C{^{1}H}-NMR spectrum (CDCl$_3$, 102 MHz) of **2-Fc$_3$-^{Et}TAT**.

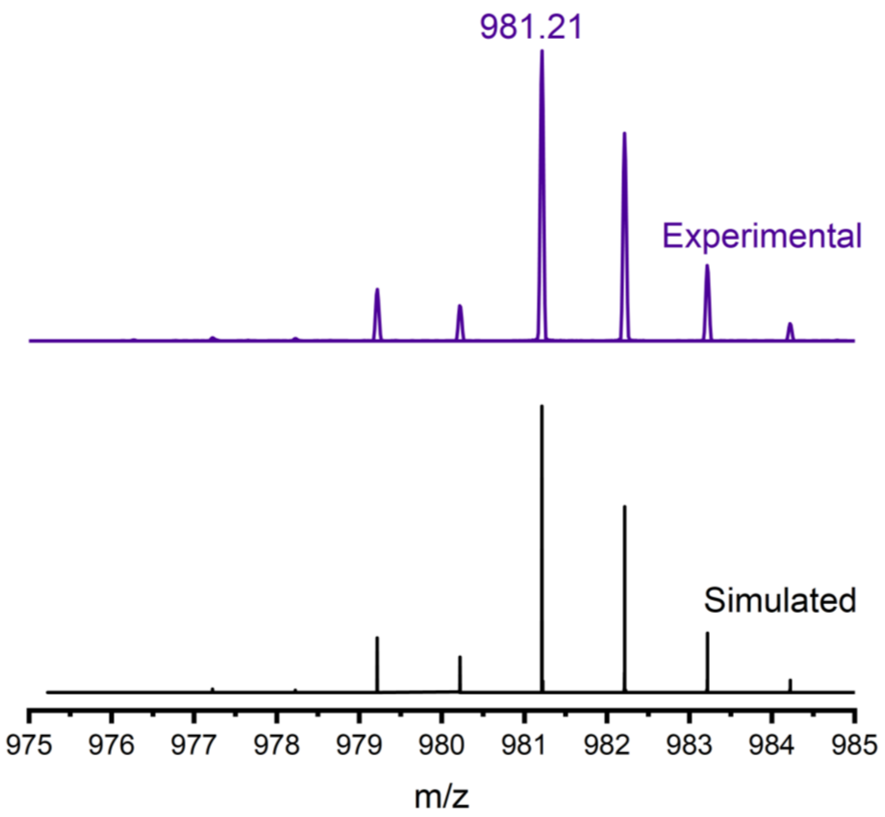

Figure S15. Molecular ion peak in the ESI mass spectrum (CH_2Cl_2) of **2-Fc_3-^{Et}TAT**.

3-Fc_3-^{Et}TAT

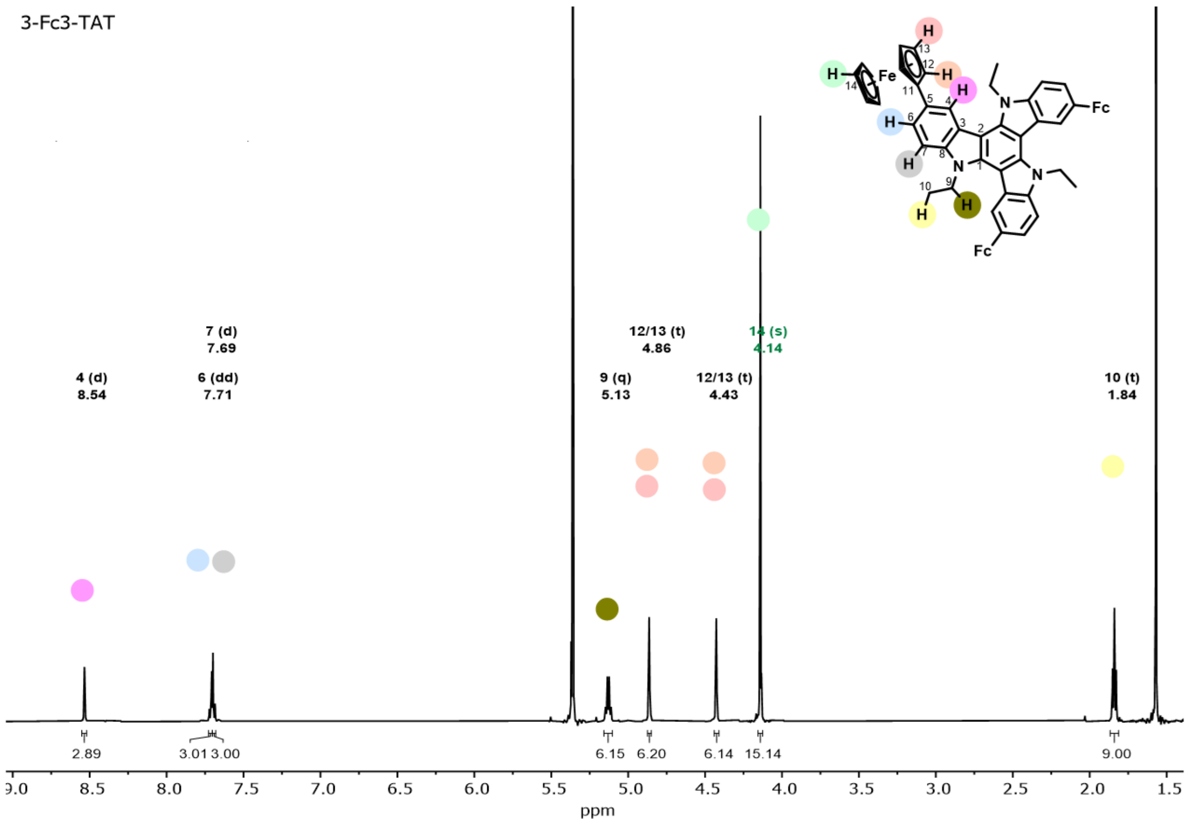

Figure S16. ^{1}H-NMR spectrum (CD_2Cl_2, 400 MHz) of **3-Fc_3-^{Et}TAT**.

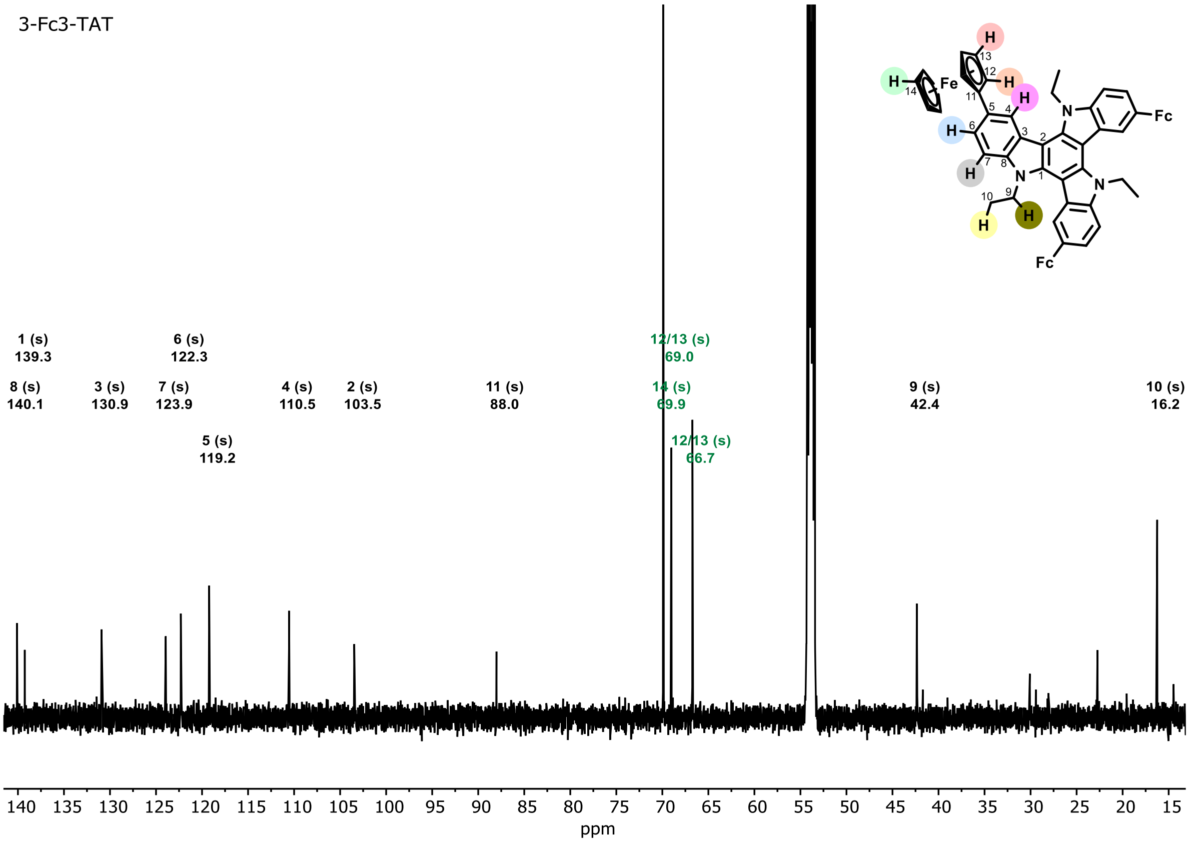

Figure S17. $^{13}C\{^1H\}$-NMR spectrum ($CDCl_3$, 101 MHz) of **3-Fc$_3$-^{Et}TAT**.

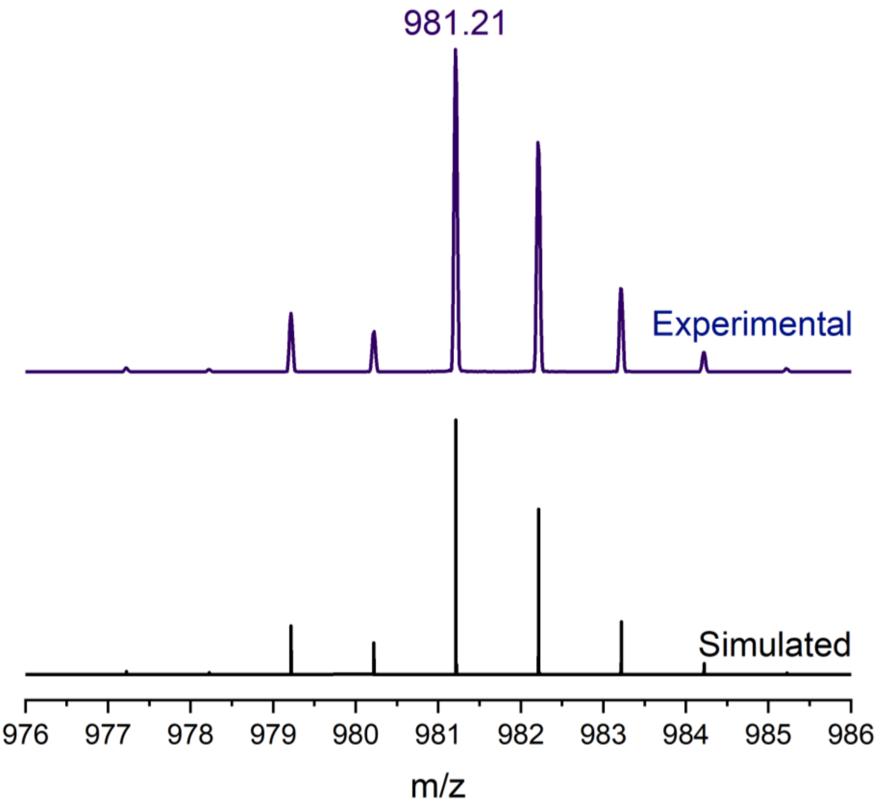

Figure S18. Molecular ion peak in the ESI mass spectrum (CH_2Cl_2) of **3-Fc$_3$-^{Et}TAT**.

2-Fc$_3$-^{Dode}TAT

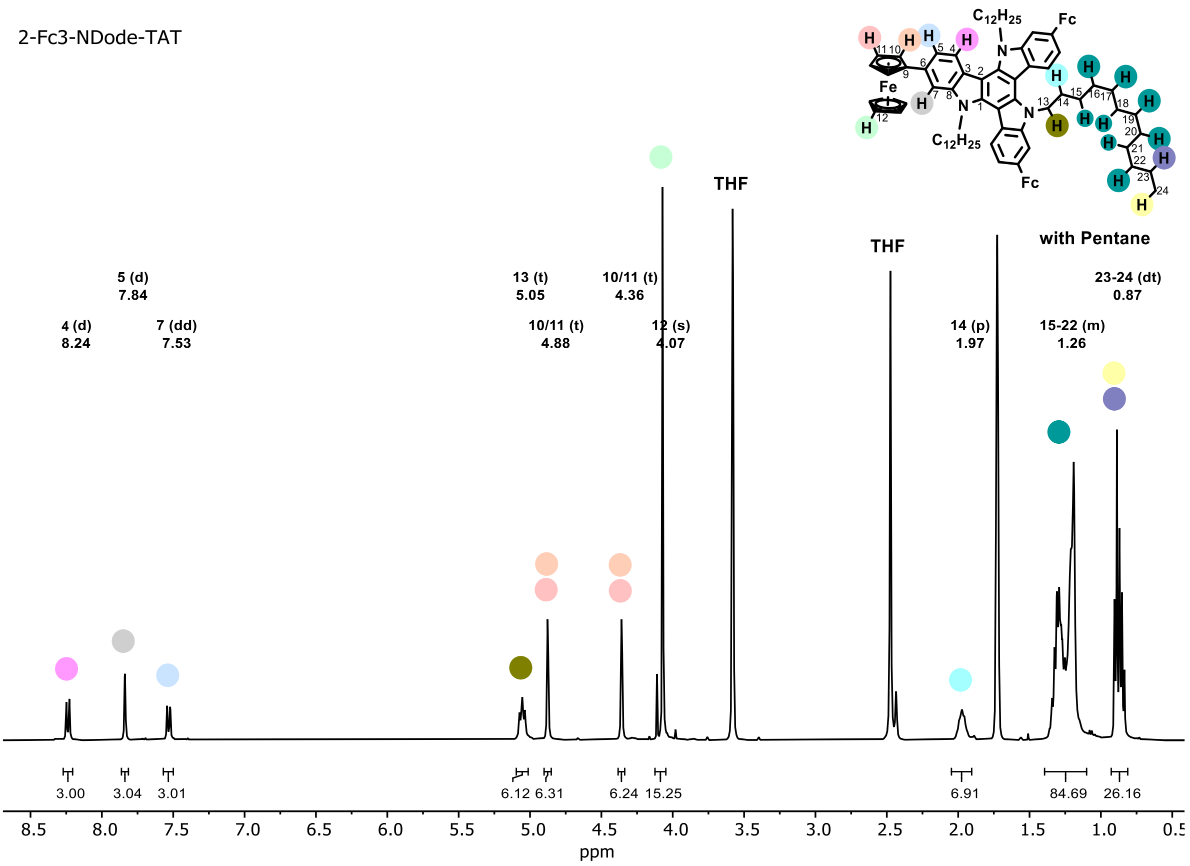

Figure S19. ^{1}H-NMR spectrum (THF-d8, 400 MHz) of **2-Fc$_3$-^{Dode}TAT**.

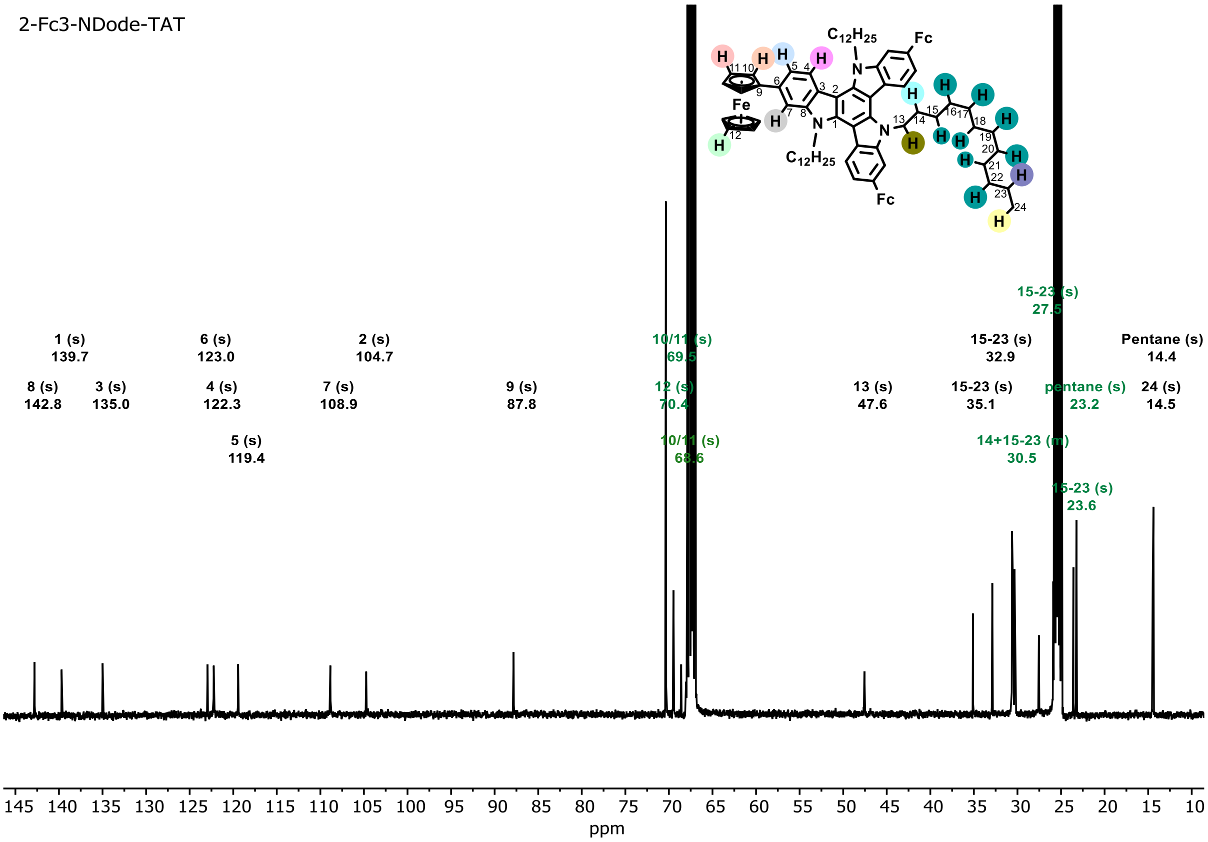

Figure S20. ^{13}C{^{1}H}-NMR spectrum (THF-d8, 101 MHz) of **2-Fc$_3$-^{Dode}TAT**.

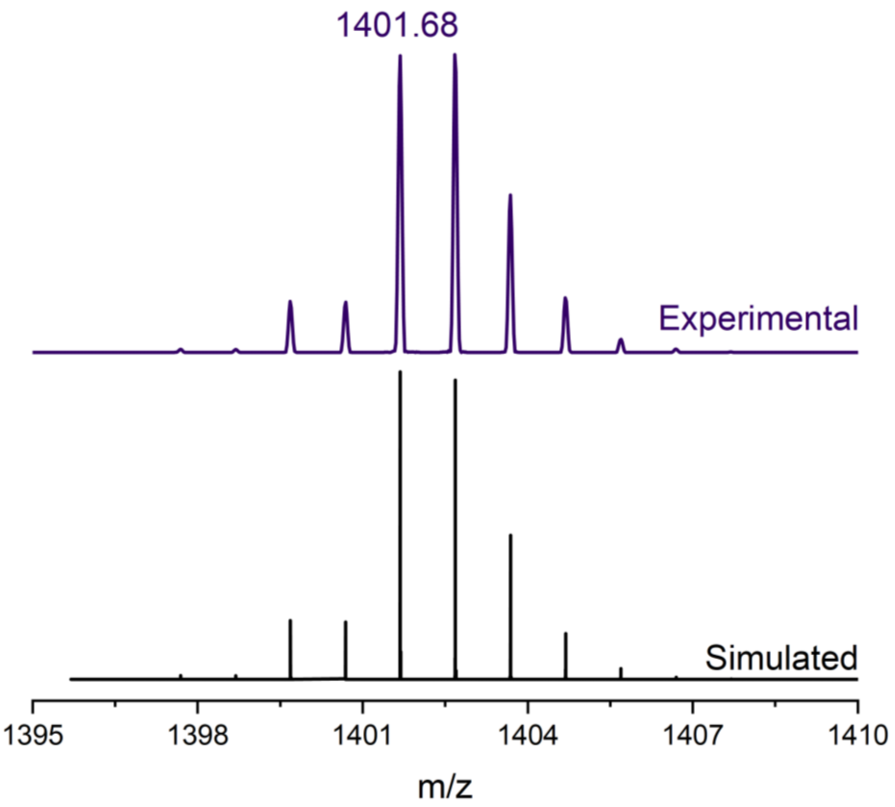

Figure S21. Molecular ion peak in the ESI mass spectrum (CH_2Cl_2) of **2-Fc$_3$-^{Dode}TAT**.

2-(Fc-A)$_1$-^{Et}TAT

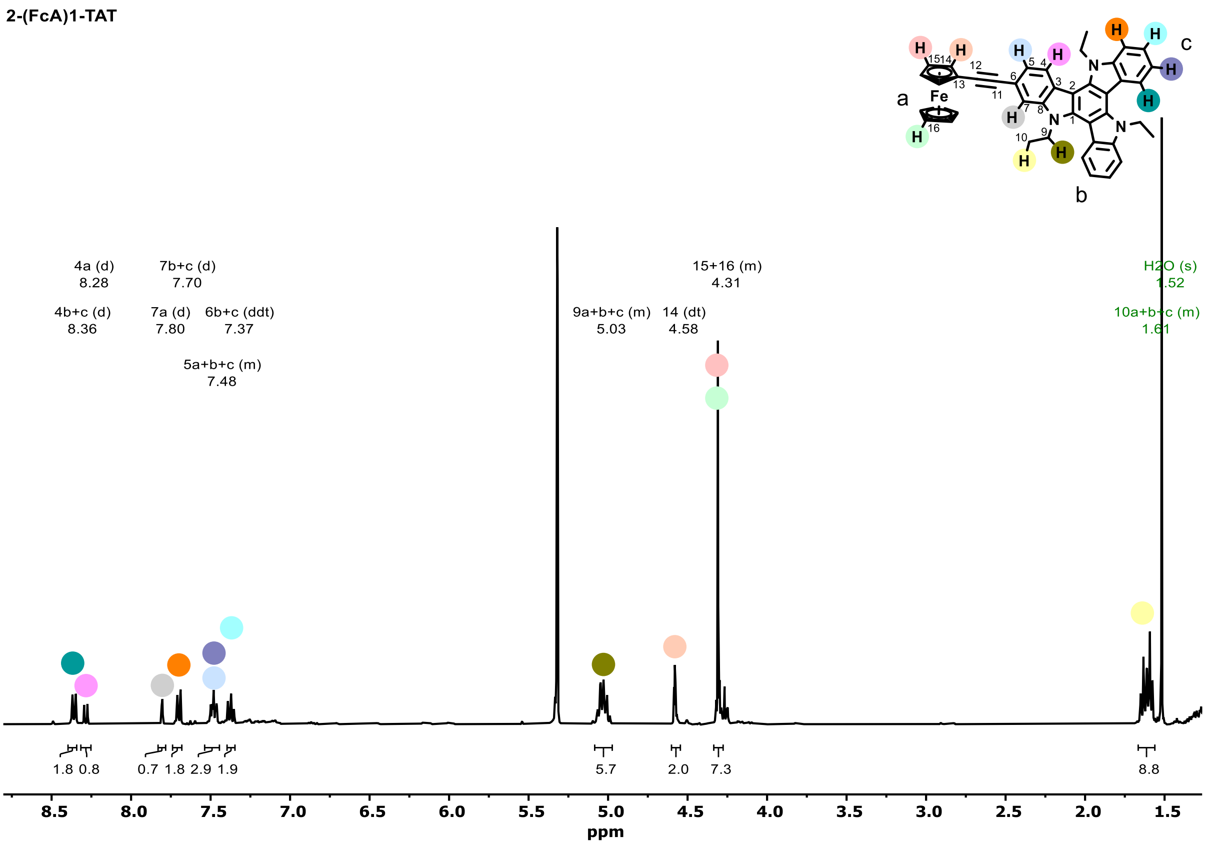

Figure S22. ^1H-NMR spectrum (CD_2Cl_2, 400 MHz) of **2-(Fc-A)$_1$-^{Et}TAT**.

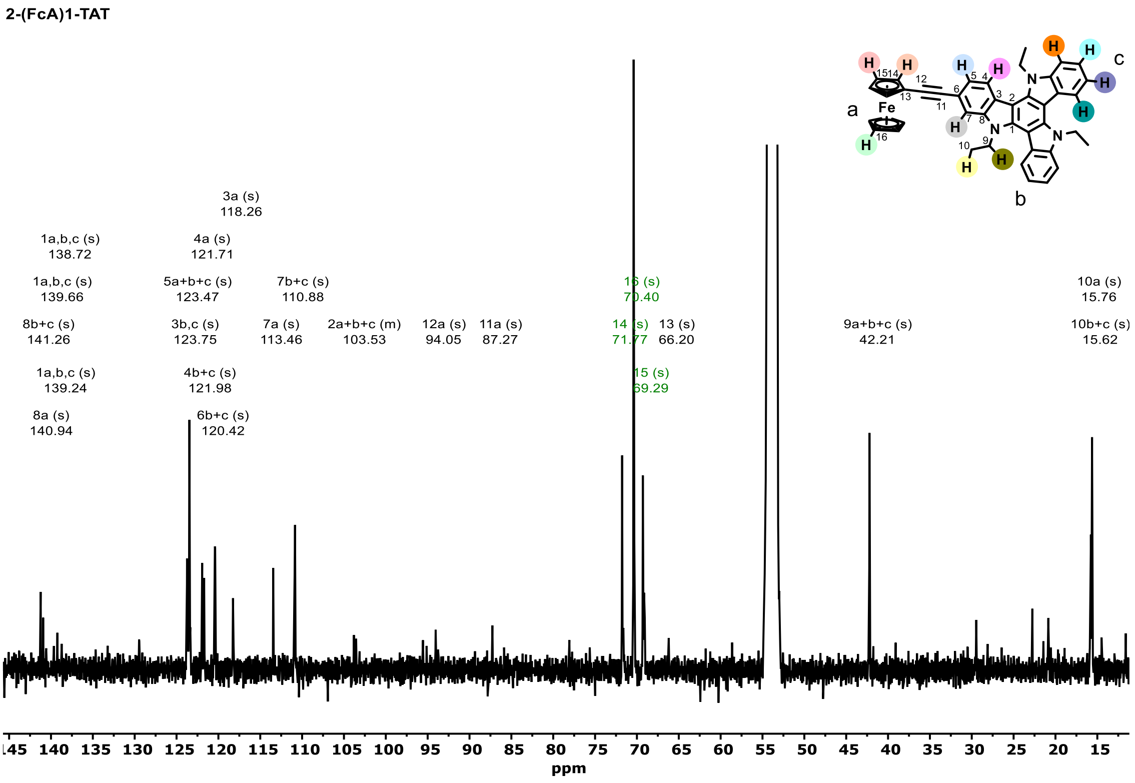

Figure S23. $^{13}C\{^1H\}$-NMR spectrum (CD_2Cl_2, 101 MHz) of **2-(Fc-A)$_1$-^{Et}TAT**.

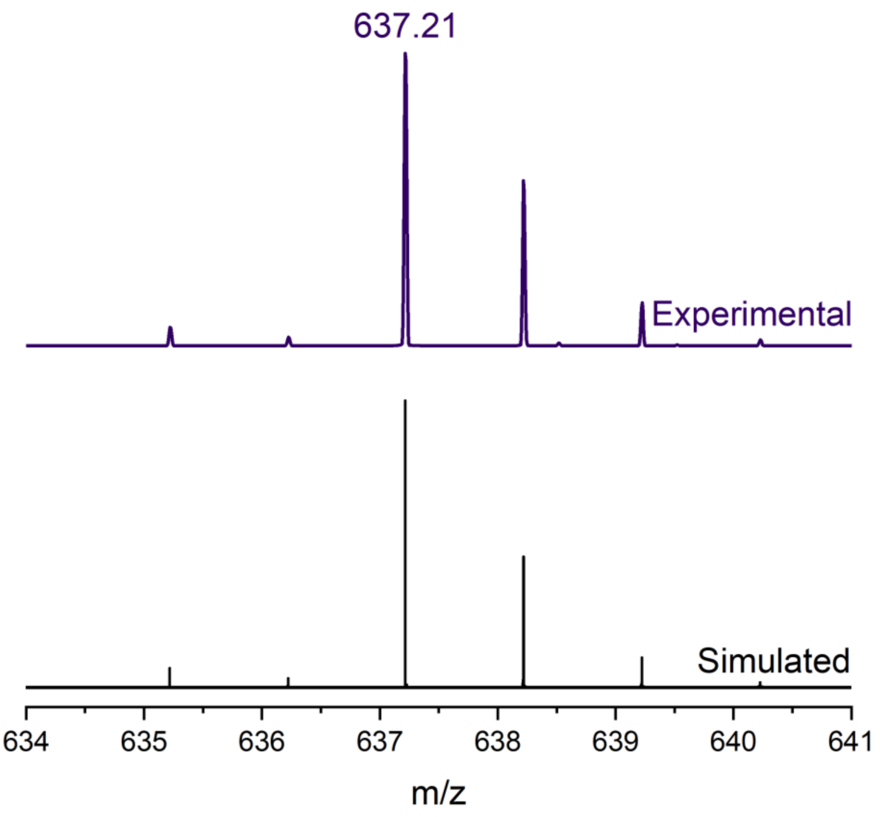

Figure S24. Molecular ion peak in the ESI mass spectrum (CH_2Cl_2) of **2-(Fc-A)$_1$-^{Et}TAT**.

2-(Fc-A)$_2$-^{Et}TAT

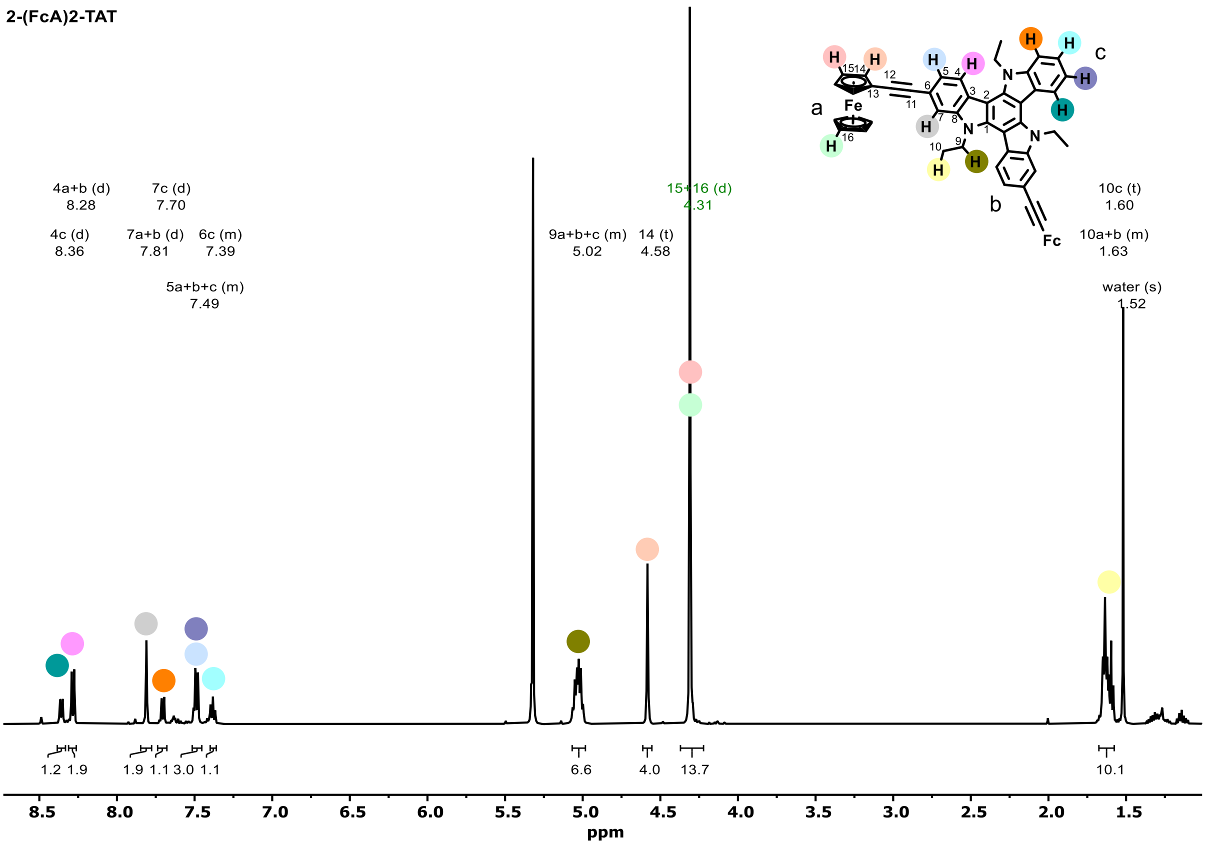

Figure S25. ^{1}H-NMR spectrum (CD_2Cl_2, 400 MHz) **of 2-(Fc-A)$_2$-^{Et}TAT**.

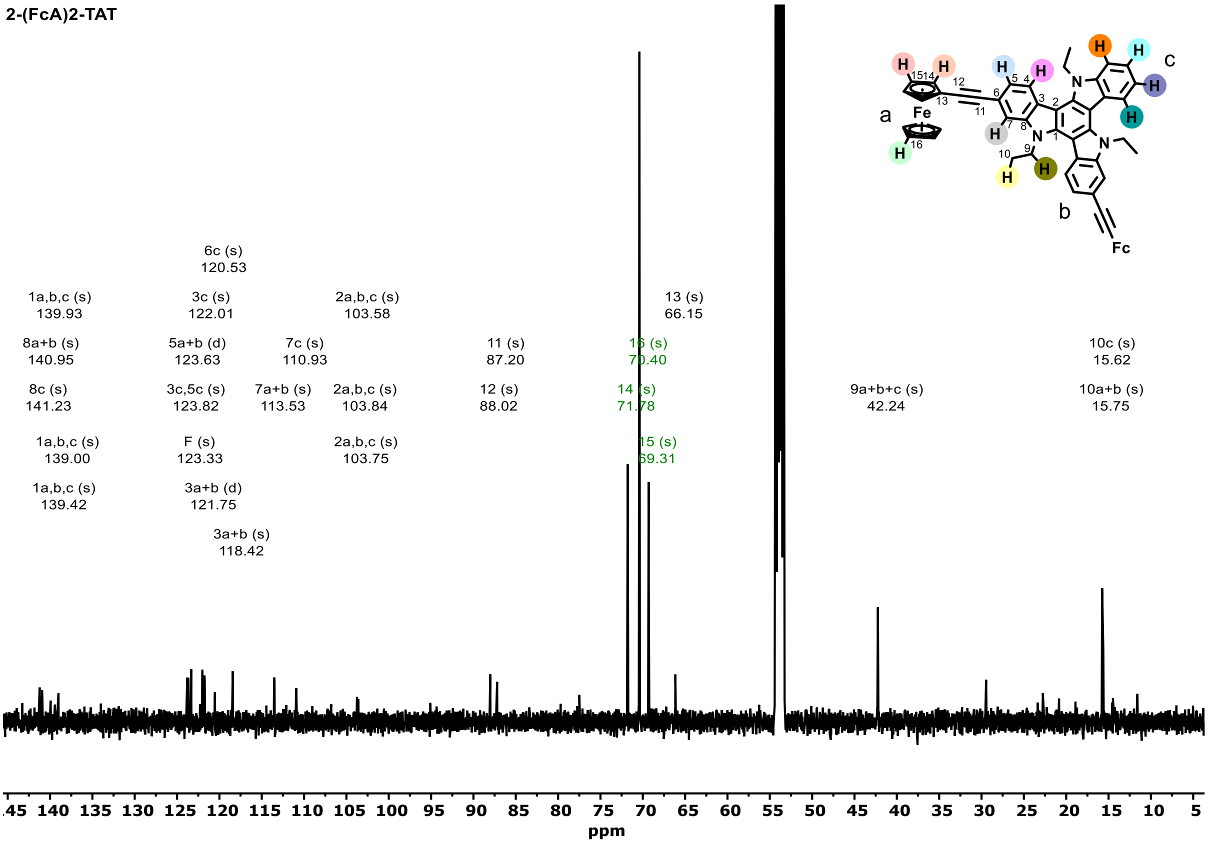

Figure S26. ^{13}C{^{1}H}-NMR spectrum (CD_2Cl_2, 101 MHz) of **2-(Fc-A)$_2$-^{Et}TAT**.

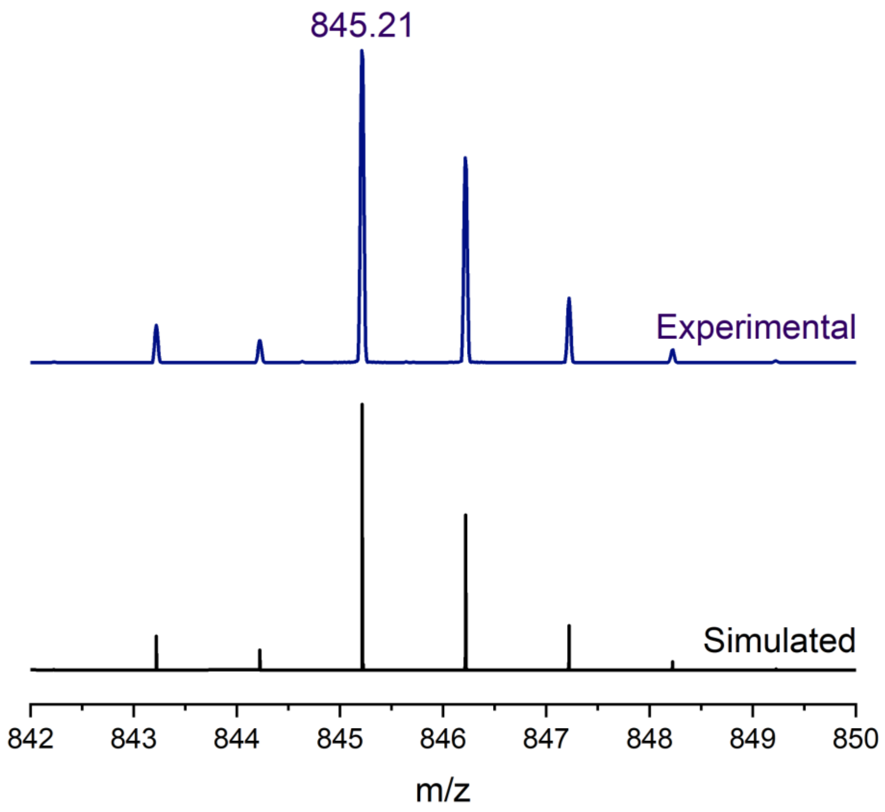

Figure S27. Molecular ion peak in the ESI mass spectrum (CH_2Cl_2) of **2-(Fc-A)$_2$-^{Et}TAT**.

2-(Fc-A)$_3$-^{Et}TAT

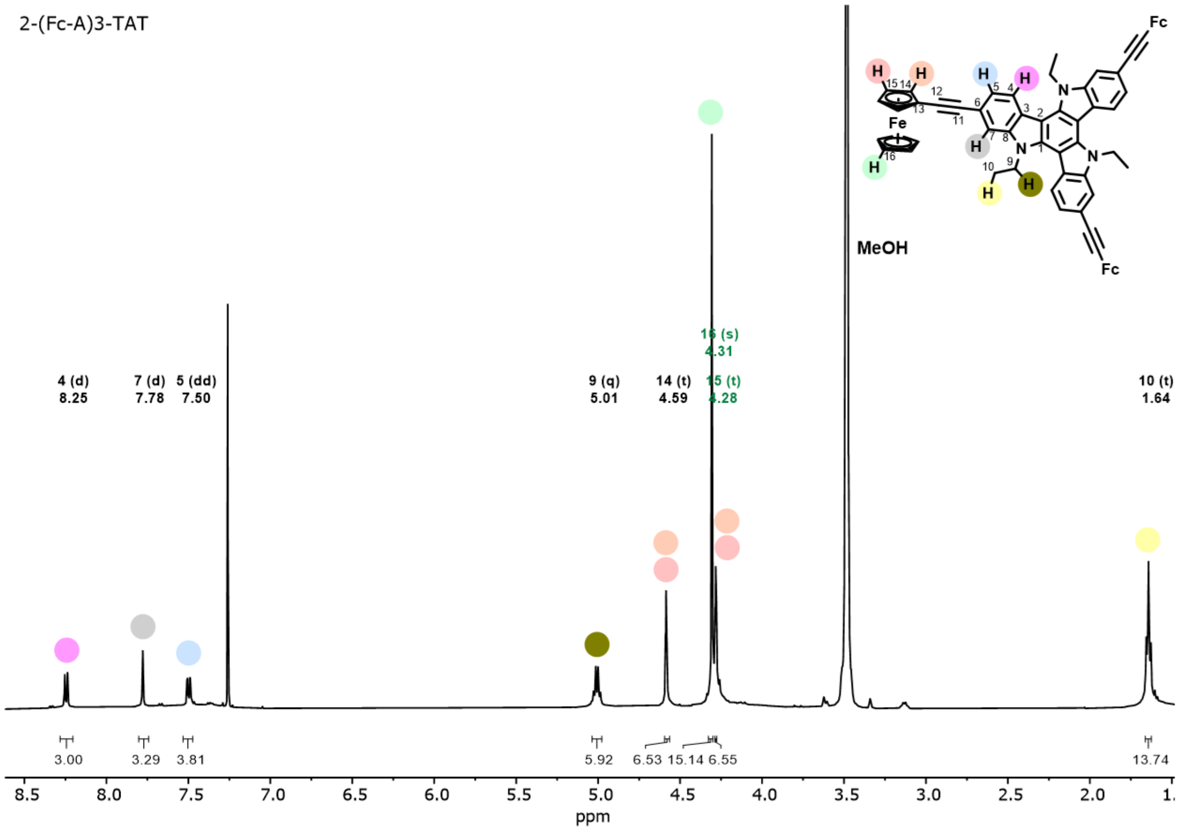

Figure S28. ^{1}H-NMR spectrum ($CDCl_3$, 400 MHz) of **2-(Fc-A)$_3$-^{Et}TAT**.

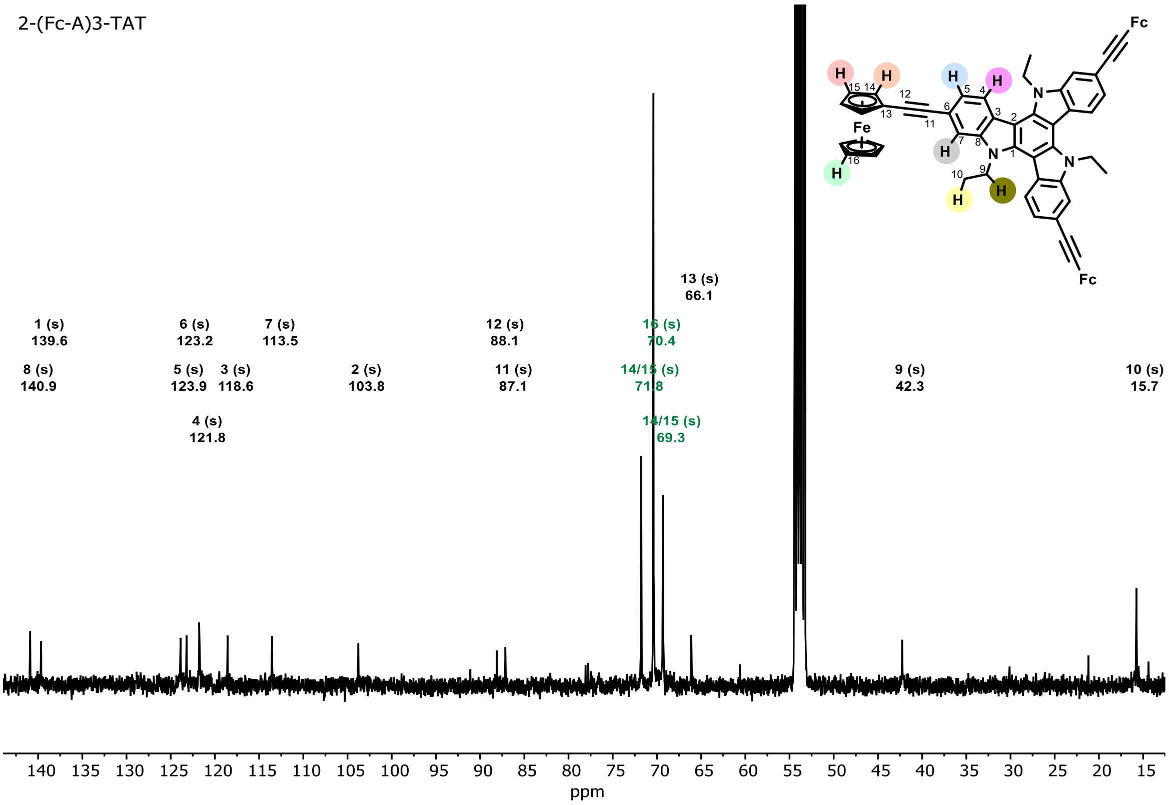

Figure S29. $^{13}C\{^1H\}$-NMR spectrum ($CDCl_3$, 101 MHz) of **2-(Fc-A)$_3$-^{Et}TAT**.

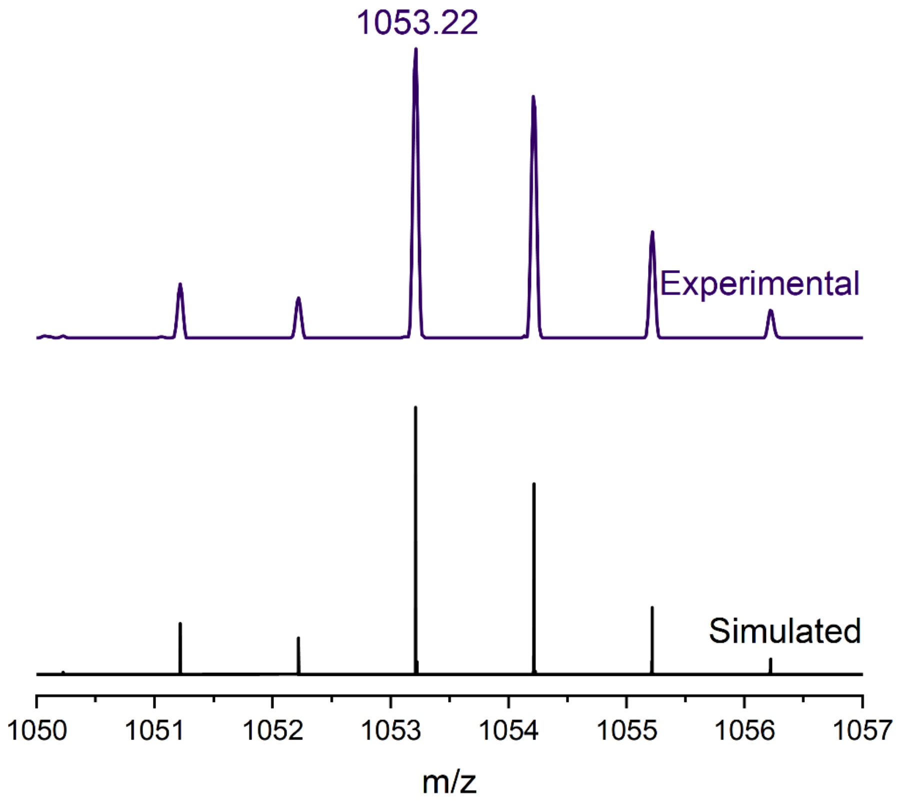

Figure S30. Molecular ion peak in the ESI mass spectrum (CH_2Cl_2) of **2-(Fc-A)$_3$-^{Et}TAT**.

3-(Fc-A)$_3$-^{Et}TAT

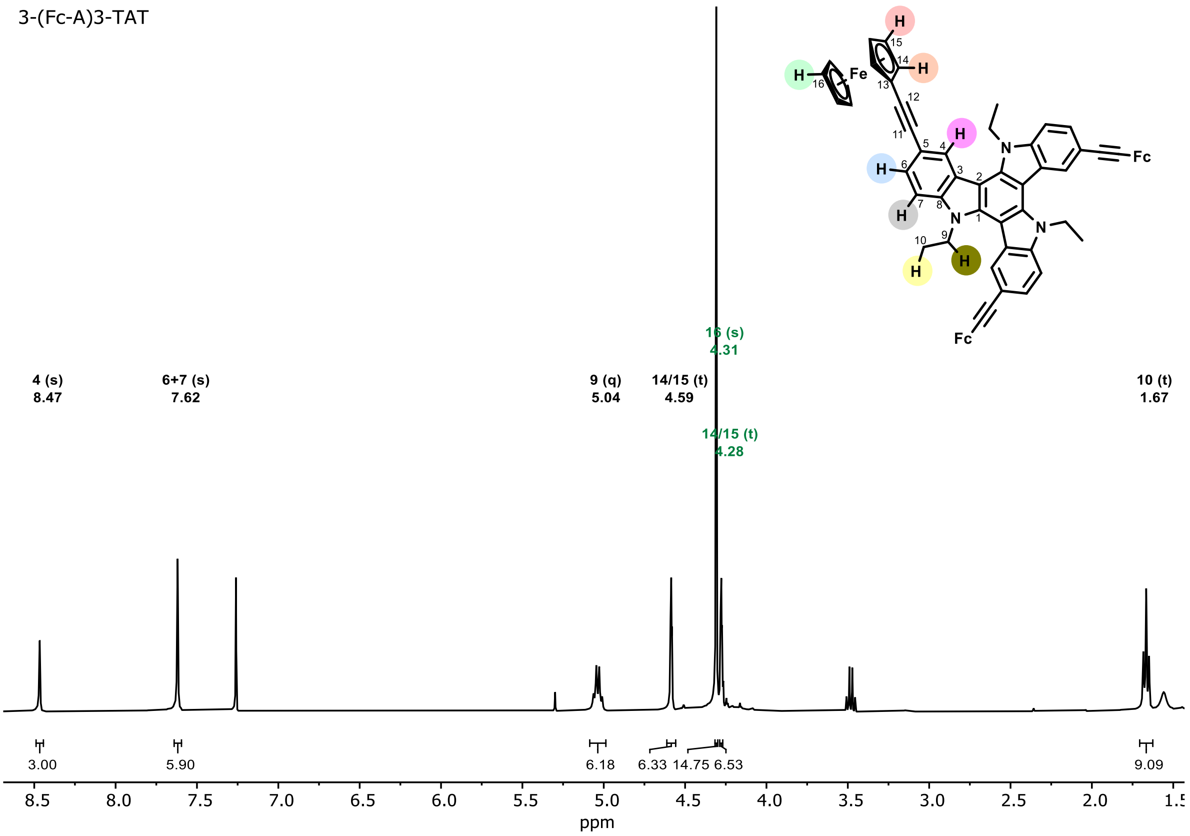

Figure S31. ^{1}H-NMR spectrum ($CDCl_3$, 400 MHz) of **3-(Fc-A)$_3$-^{Et}TAT**.

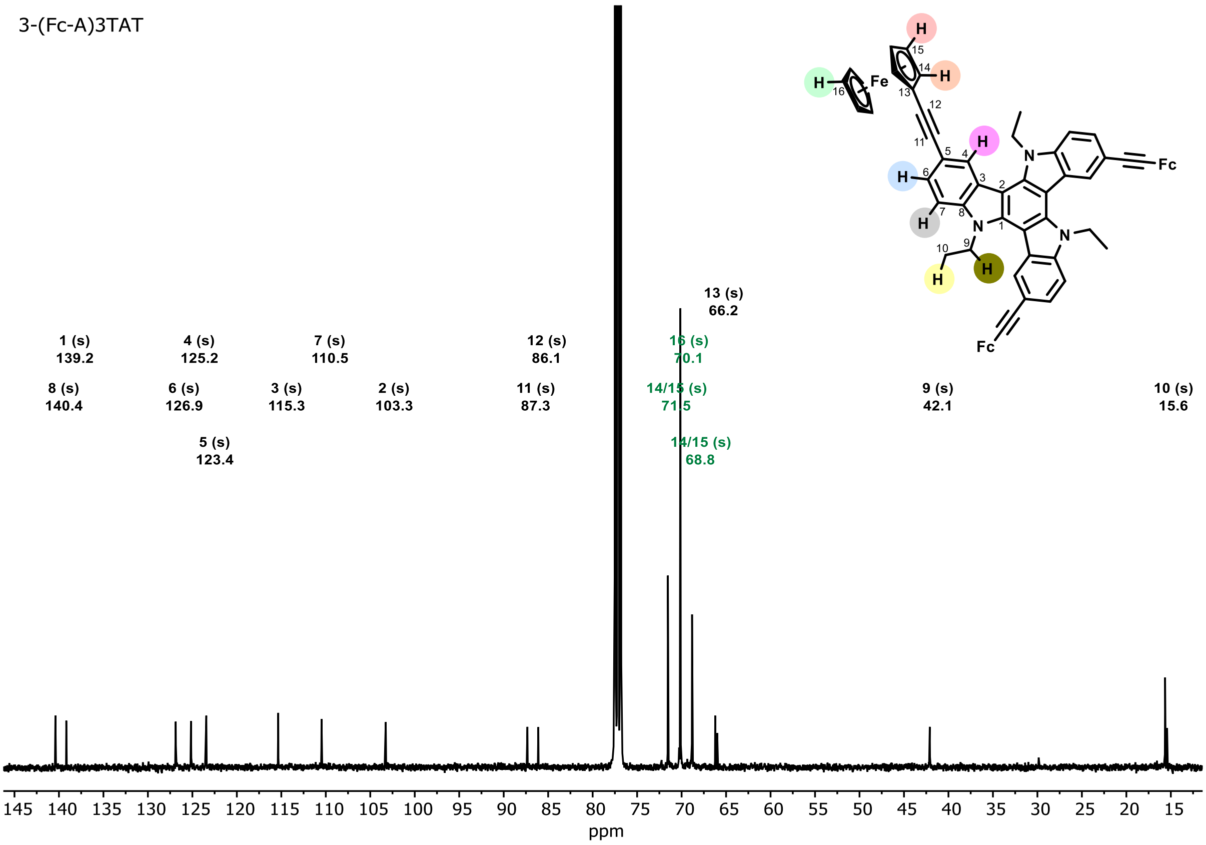

Figure S32. ^{13}C{^{1}H}-NMR spectrum ($CDCl_3$, 101 MHz) of **3-(Fc-A)$_3$-^{Et}TAT**.

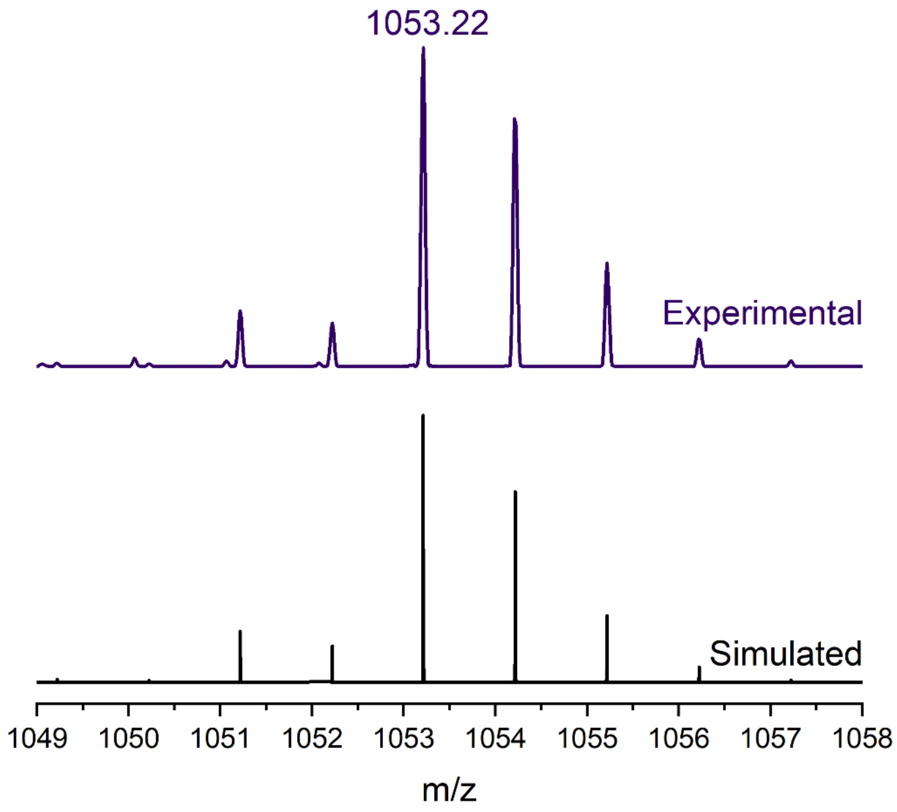

Figure S33. Molecular ion peak in the ESI mass spectrum (CH_2Cl_2) of **3-(Fc-A)$_3$-^{Et}TAT**.

Voltammetric measurements

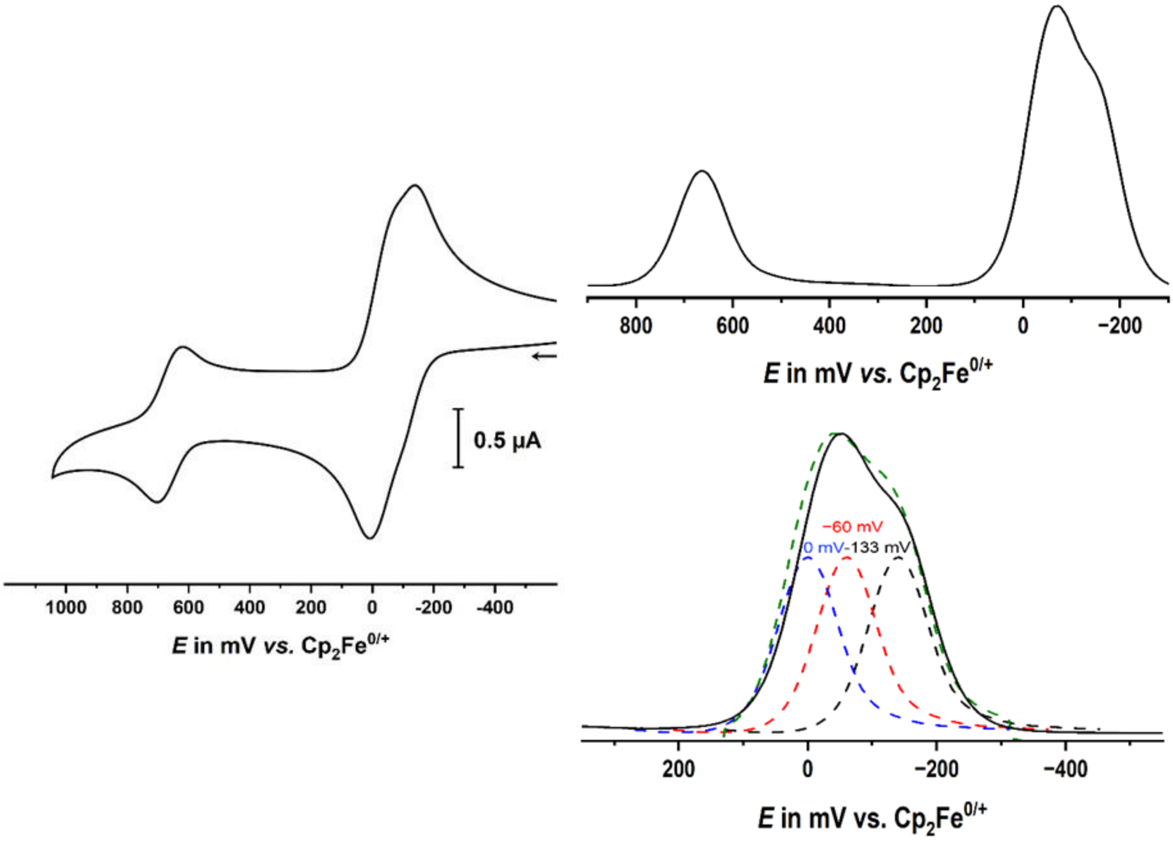

Figure S34. Left: Cyclic voltammogram of **2-Fc3-^{Et}TAT**, **1** (*v* = 100 mV/s, CH_2Cl_2, 0.04 M NBu_4^+ $[B\{C_6H_3(CF_3)_2\text{-}3,5\}_4]^-$, r. t.); top right: Square wave voltammogram of **2-Fc3-^{Et}TAT**, **1** (frequency = 15 Hz, potential step = 4 mV, square wave amplitude = 25 mV); bottom right: Deconvolution of the experimental square wave voltammogram (black line) by three square-wave peaks at -133, -60 and 0 mV; the resulting envelope is shown as the green dashed line.

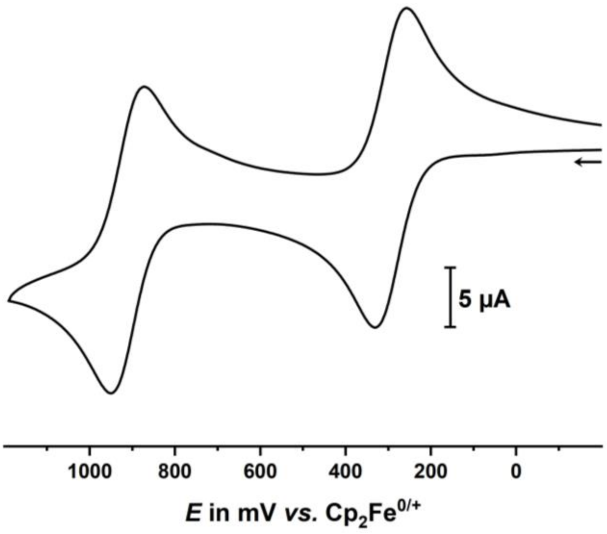

Figure S35. Cyclic voltammogram of **^{Et}TAT** (*v* = 100 mV/s, CH_2Cl_2, 0.06 M nBu_4PF_6, r. t.).

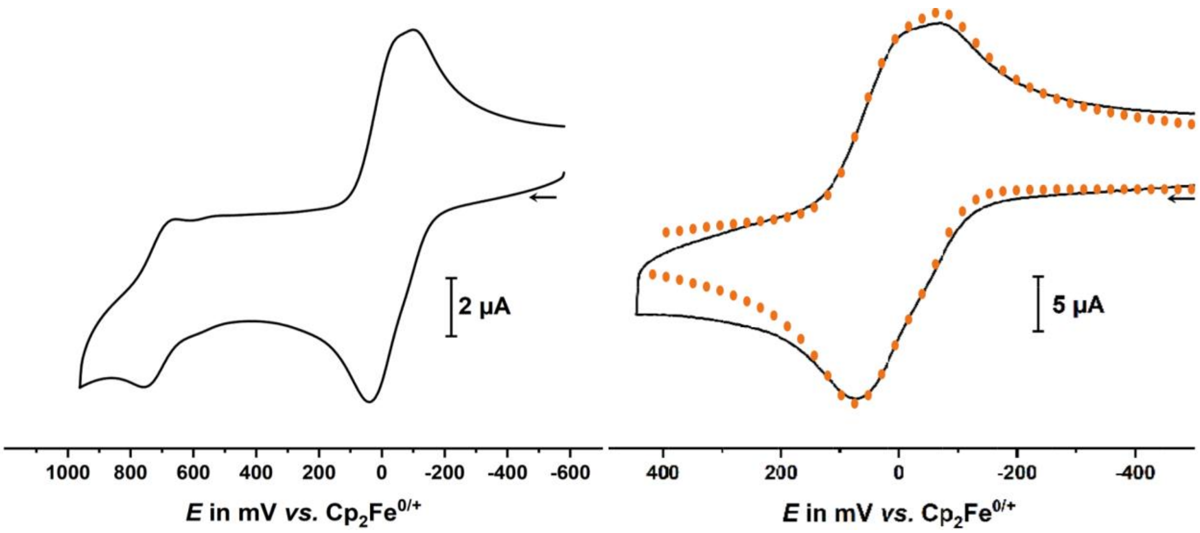

Figure S36. Left: Cyclic voltammogram of **2-Fc$_3$-^{Dode}TAT** (*v* = 100 mV/s, CH_2Cl_2, 0.05 M NBu_4^+ $[B\{C_6H_3(CF_3)_2$-$3,5\}_4]^-$, r. t.). Right. Overlay of the experimental (black line) and simulated (red dots) voltammograms of the ferrocenyl-based anodic waves of **2-Fc$_3$-^{Dode}TAT** (*v* = 800 mV/s).

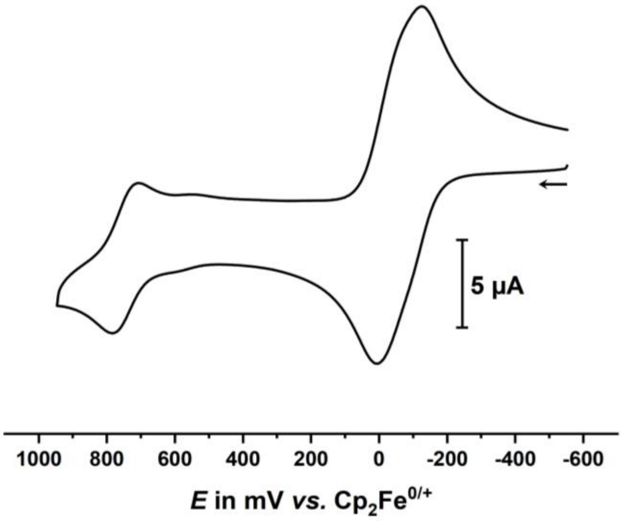

Figure S37. Cyclic voltammogram of **3-Fc$_3$-^{Et}TAT** (*v* = 100 mV/s, CH_2Cl_2, 0.05 M NBu_4^+ $[B\{C_6H_3(CF_3)_2$-$3,5\}_4]^-$, r. t.).

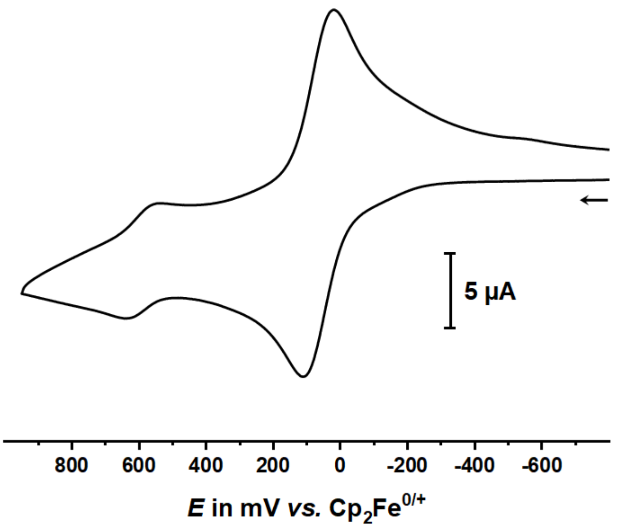

Figure S38. Cyclic voltammogram of **3-(FcA)$_3$-^{Et}TAT** (*v* = 100 mV/s, CH_2Cl_2, 0.05 M NBu_4^+ $[B\{C_6H_3(CF_3)_2\text{-}3,5\}_4]^-$, r. t.).

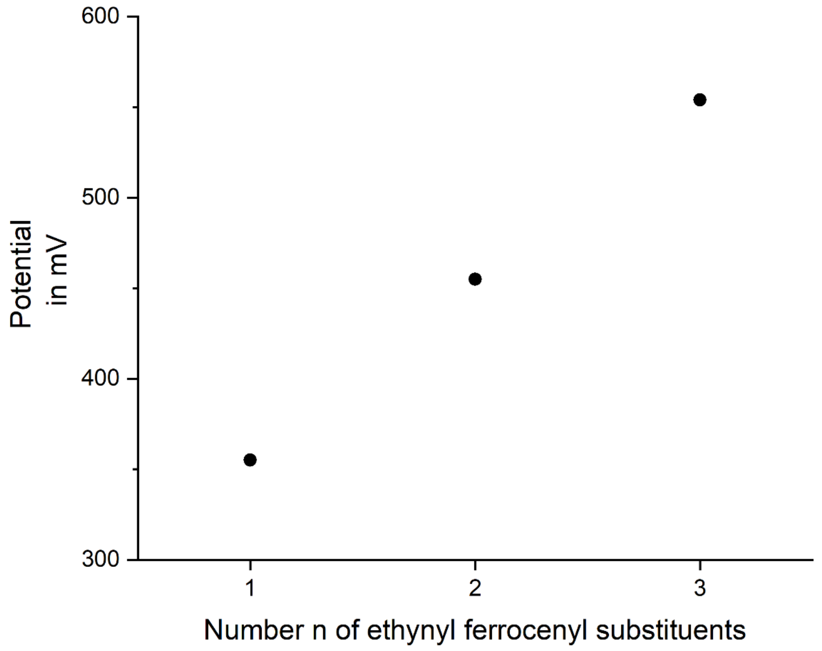

Figure S39. Half-wave potential for the TAT/TAT$^+$ wave in the ferrocenylethynyl-substituted complexes **2-(Fc-A)$_n$-^{Et}TAT** (*v* = 100 mV/s, CH_2Cl_2, 0.05 M NBu_4^+ $[B\{C_6H_3(CF_3)_2\text{-}3,5\}_4]^-$). Potentials are given on the $Cp_2Fe^{0/+}$ scale.

UV/Vis/NIR and IR Spectroelectrochemistry

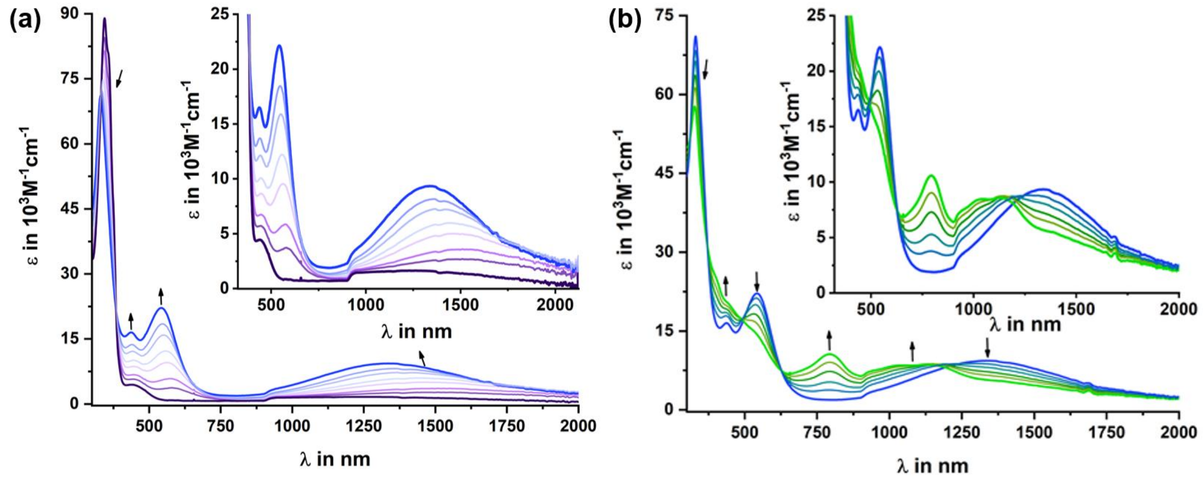

Figure S40. (a) Changes in the UV/Vis/NIR-spectrum of **2-Fc$_3$^{Et}TAT** (0.14 M NBu_4^+ $[B\{C_6H_3(CF_3)_2\text{-}3,5\}_4]^-$ in 1,2-$C_2H_4Cl_2$) during (a) sequential oxidation of the ferrocenyl moieties to ferrocenium units and (b) oxidation of the TAT core. Insets show a magnification of the bands with lower oscillator strengths.

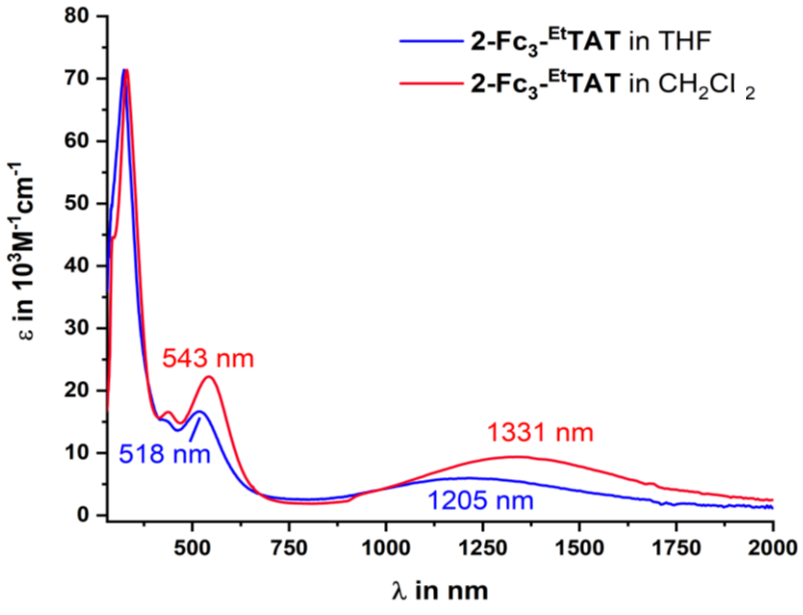

Figure S41. UV/Vis/NIR-spectra of **2-Fc$_3$^{Et}TAT^{3+}** in CH_2Cl_2 and in THF.

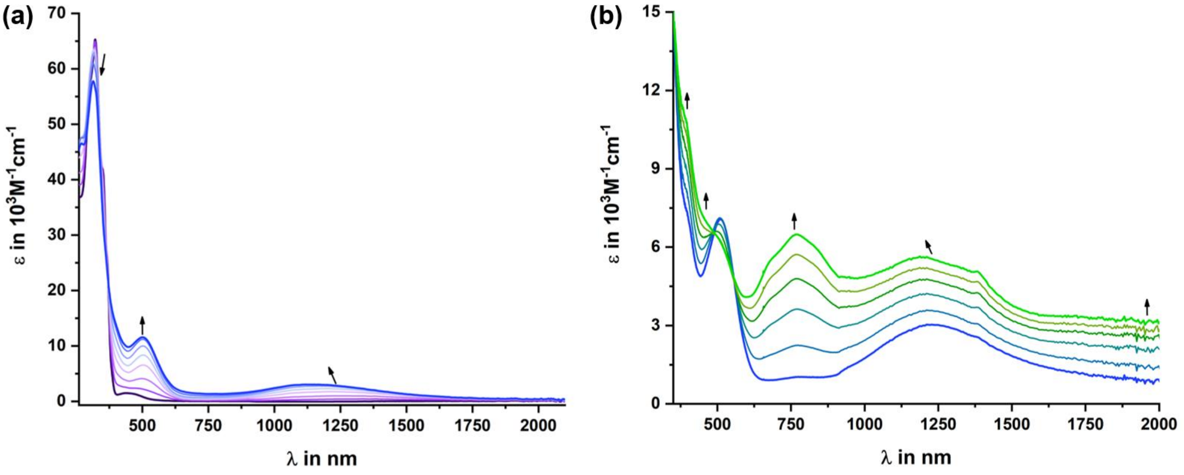

Figure S42. a) Changes in the UV/Vis/NIR-spectrum of **3-Fc$_3$^{Et}TAT** (0.14 M NBu_4^+ $[B\{C_6H_3(CF_3)_2\text{-}3,5\}_4]^-$ in 1,2-$C_2H_4Cl_2$) during (a) sequential oxidation of the ferrocenyl moieties to ferrocenium units and (b) oxidation of the TAT core.

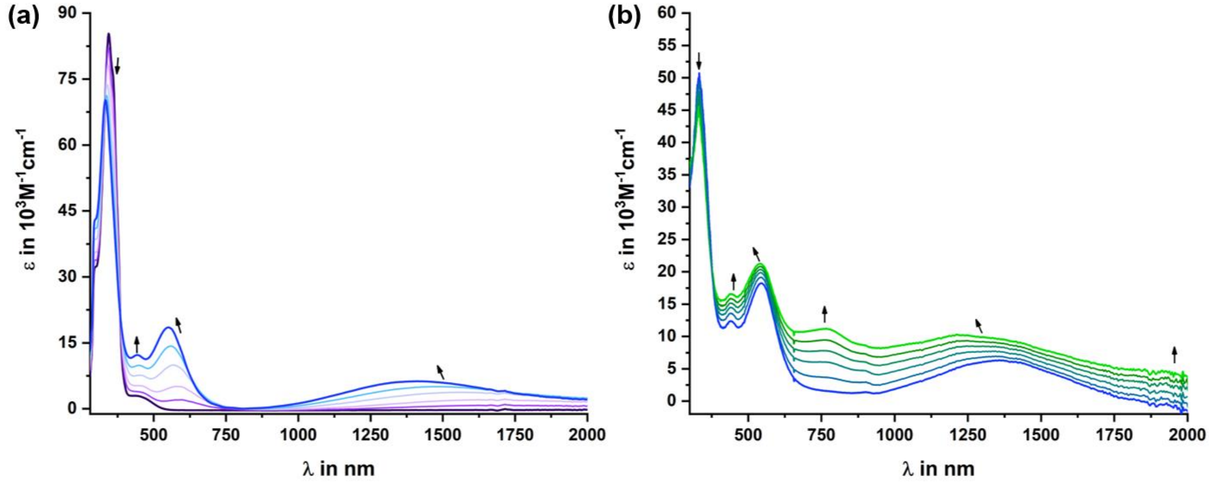

Figure S43. a) Changes in the UV/Vis/NIR-spectrum of **2-Fc$_3$^{Dode}TAT** (0.14 M NBu_4^+ $[B\{C_6H_3(CF_3)_2\text{-}3,5\}_4]^-$ in 1,2-$C_2H_4Cl_2$) during (a) sequential oxidation of the ferrocenyl moieties to ferrocenium units and (b) oxidation of the TAT core.

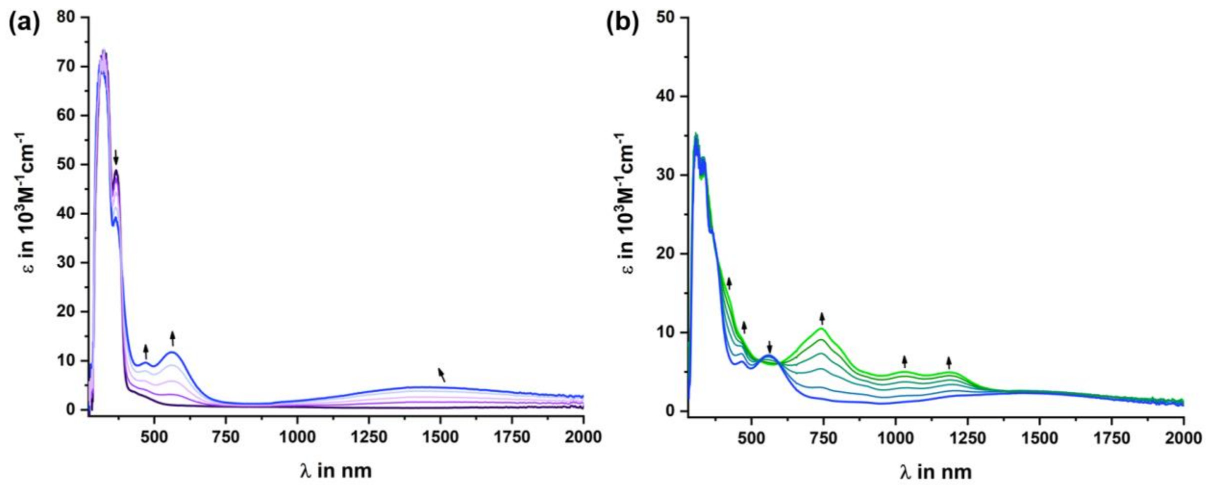

Figure S44. a) Changes in the UV/Vis/NIR-spectrum of **2-(FcA)$_1$-^{Et}TAT** (0.14 M NBu_4^+ $[B\{C_6H_3(CF_3)_2\text{-}3,5\}_4]^-$ in 1,2-$C_2H_4Cl_2$) during (a) sequential oxidation of the ferrocenyl moieties to ferrocenium units and (b) oxidation of the TAT core.

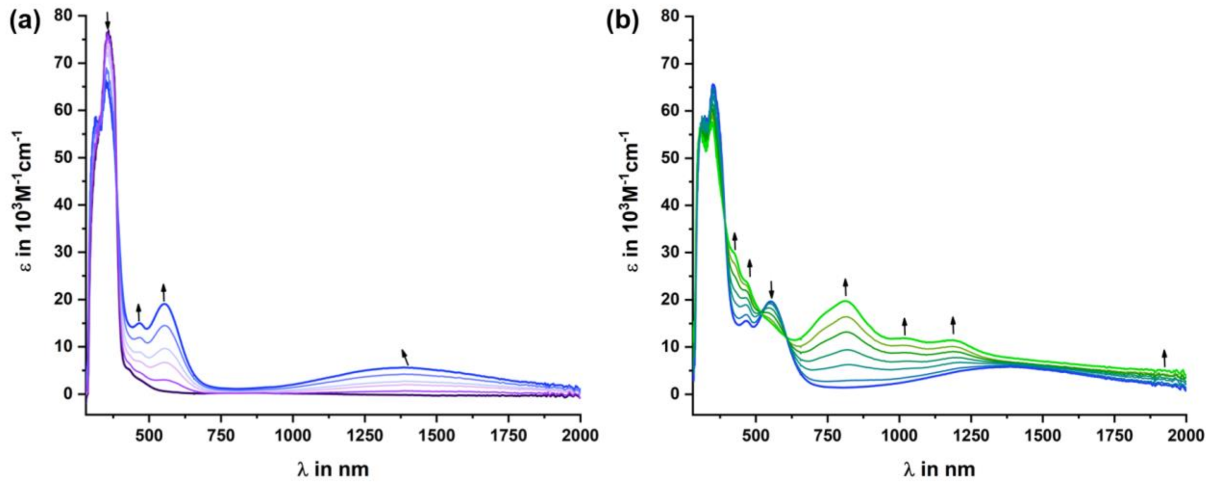

Figure S45. a) Changes in the UV/Vis/NIR-spectrum of **2-(FcA)$_2$-^{Et}TAT** (0.14 M NBu_4^+ $[B\{C_6H_3(CF_3)_2\text{-}3,5\}_4]^-$ in 1,2-$C_2H_4Cl_2$) during (a) sequential oxidation of the ferrocenyl moieties to ferrocenium units and (b) oxidation of the TAT core.

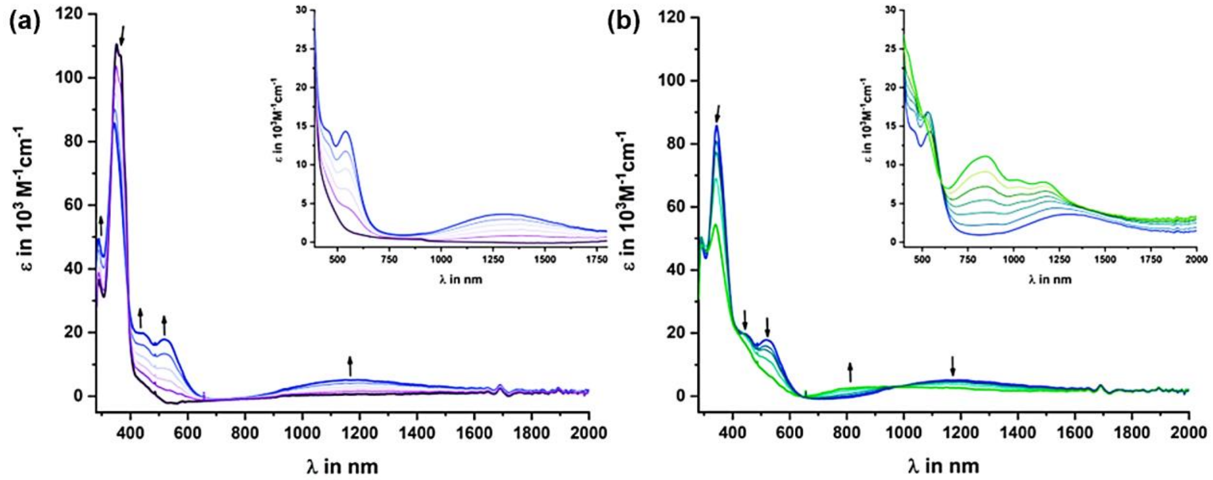

Figure S46. a) Changes in the UV/Vis/NIR-spectrum of **2-(FcA)$_3$-^{Et}TAT** (0.14 M NBu_4^+ $[B\{C_6H_3(CF_3)_2\text{-}3,5\}_4]^-$ in 1,2-$C_2H_4Cl_2$) during (a) sequential oxidation of the ferrocenyl moieties to ferrocenium units and (b) oxidation of the TAT core. Insets show a magnification of the bands with lower oscillator strengths.

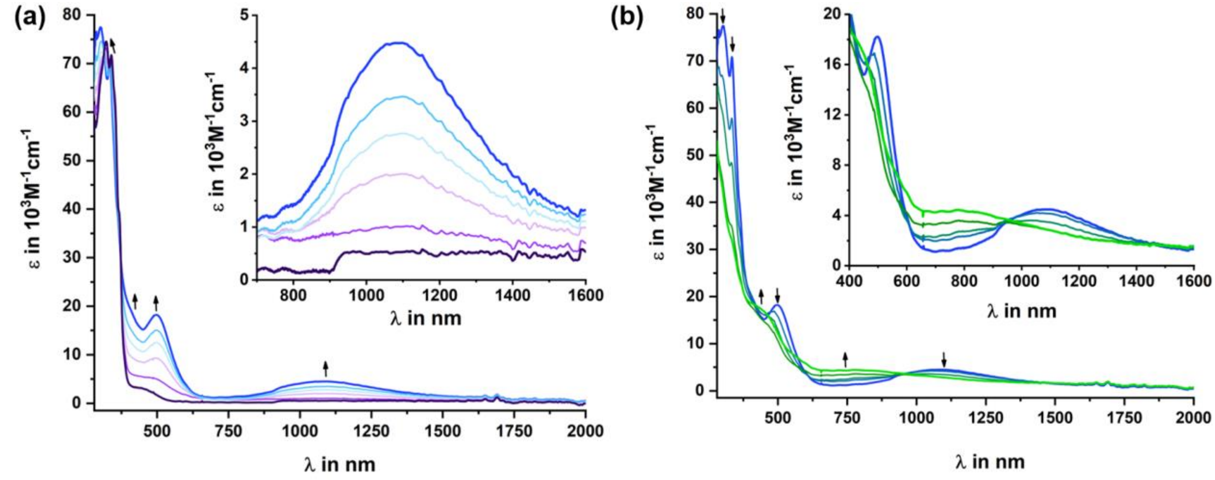

Figure S47. a) Changes in the UV/Vis/NIR-spectrum of **3-(FcA)$_3$-^{Et}TAT** (0.14 M NBu_4^+ $[B\{C_6H_3(CF_3)_2\text{-}3,5\}_4]^-$ in 1,2-$C_2H_4Cl_2$) during (a) sequential oxidation of the ferrocenyl moieties to ferrocenium units and (b) oxidation of the TAT core. Insets show a magnification of the bands with lower oscillator strengths.

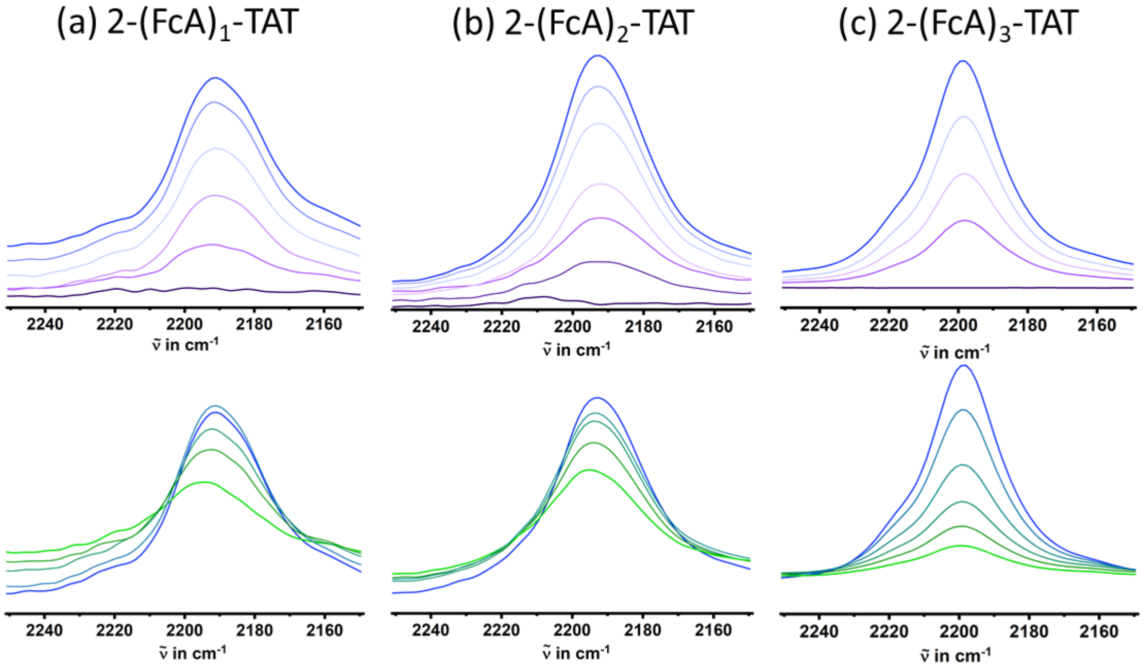

Figure S48. Changes in the IR spectra (range of the ethynyl C≡C stretching vibrations) for ferrocene- (top panels) and TAT-based oxidations (bottom panels) of (a) **2-(FcA)$_1$-^{Et}TAT**, (b) **2-(FcA)$_2$-^{Et}TAT**, and (c) **2-(FcA)$_3$-^{Et}TAT** recorded during electrolysis in an OTTLE cell (0.14 M 1,2-$C_2H_4Cl_2$/NBu_4^+ $[B\{C_6H_3(CF_3)_2\text{-}3,5\}_4]^-$, r. t).

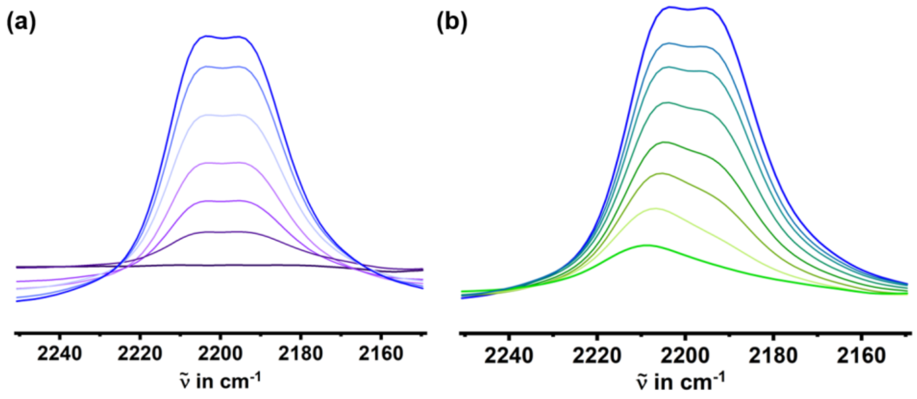

Figure S49. (a) Changes in the IR spectra (range of the ethynyl C≡C stretching vibrations) for (a) the ferrocene- (top panels) and (b) the TAT-based oxidations **3-(FcA)$_3$-^{Et}TAT** recorded during electrolysis in an OTTLE cell (0.14 M 1,2-$C_2H_4Cl_2$/NBu_4^+ $[B\{C_6H_3(CF_3)_2\text{-}3,5\}_4]^-$, r. t).

Table S2. IR-frequencies of the alkynyl stretching vibrations of the ethynylferrocenyl-substituted TATs.

	$\tilde{\nu}_{1/2}^{Fc+}$	$\tilde{\nu}_{1/2}^{TAT\cdot+}$
2-(Fc-A)$_1$-TAT	2191	2195
2-(Fc-A)$_2$-TAT	2192	2195
2-(Fc-A)$_3$-TAT	2198	2199
3-(Fc-A)$_3$-TAT	2193,2203	2209

DFT Calculations

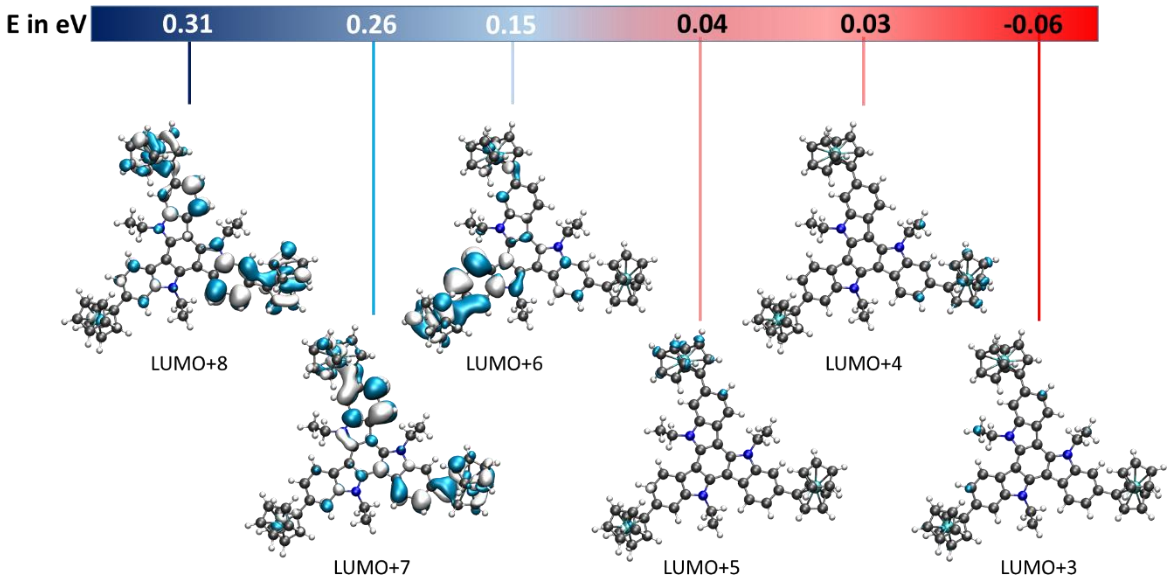

Figure S50. Contour diagrams of orbitals LUMO+3 to LUMO+8 of **2-Fc$_3$-^{Et}TAT** with their respective energies.

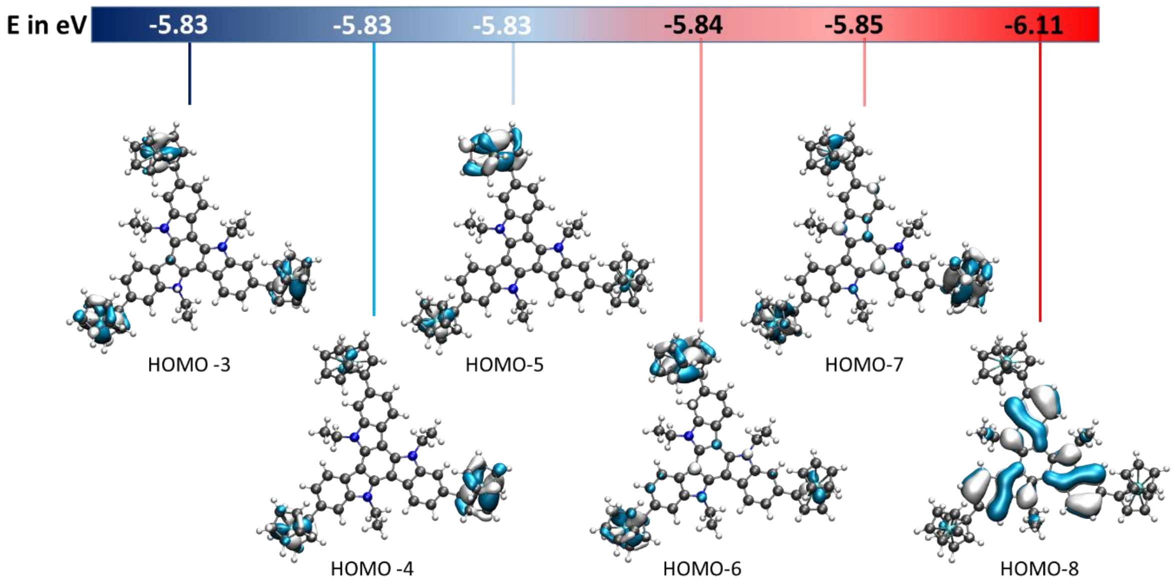

Figure S51. Contour diagrams of orbitals HOMO-8 to HOMO-3 of **2-Fc$_3$-^{Et}TAT** with their respective energies.

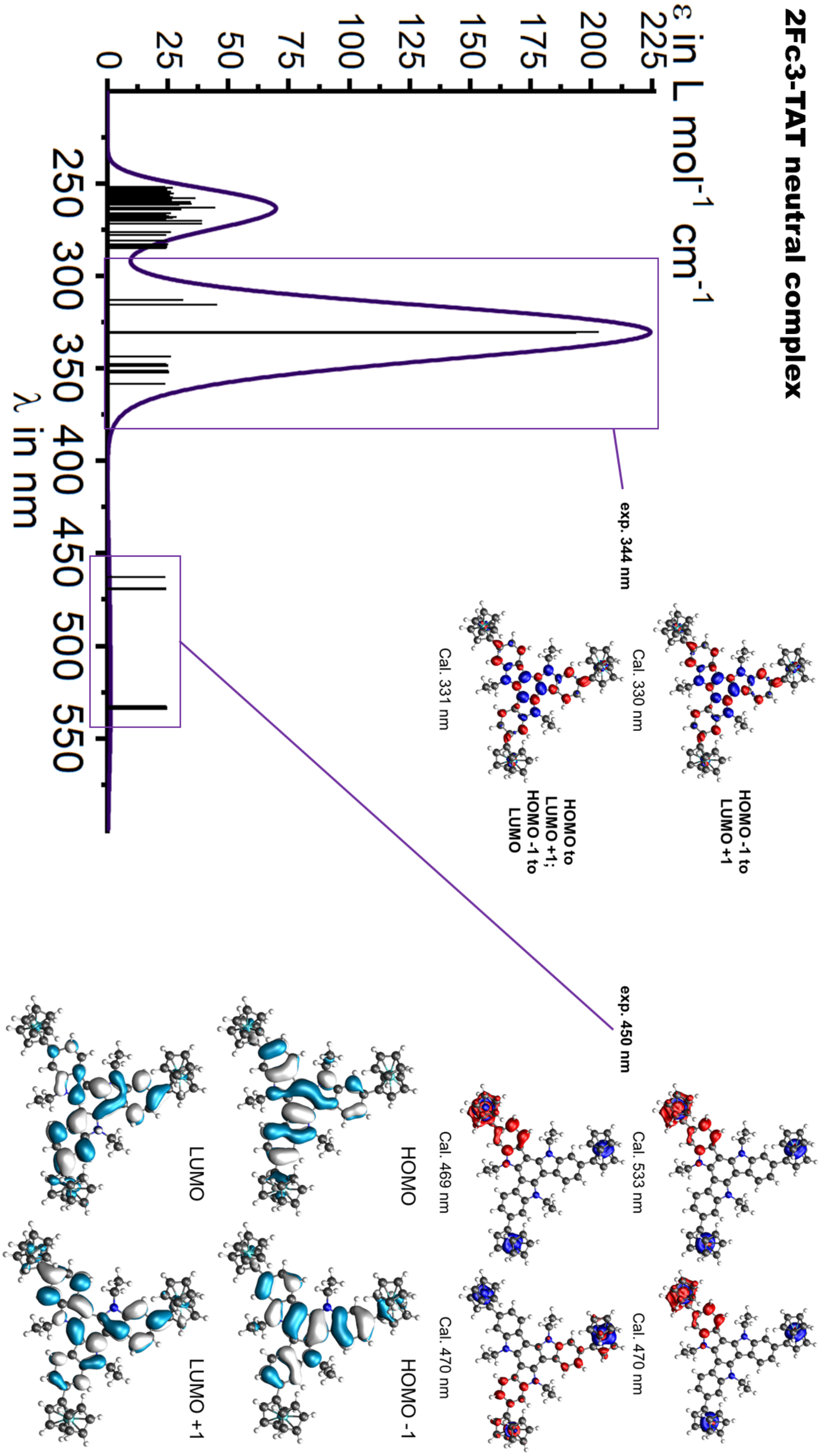

Figure S52. TD-DFT calculated UV/Vis/NIR-spectrum of neutral **2-Fc$_3$-^{Et}TAT** with EDDMs for the individual transitions. Contributing MOs and band assignments are also provided.

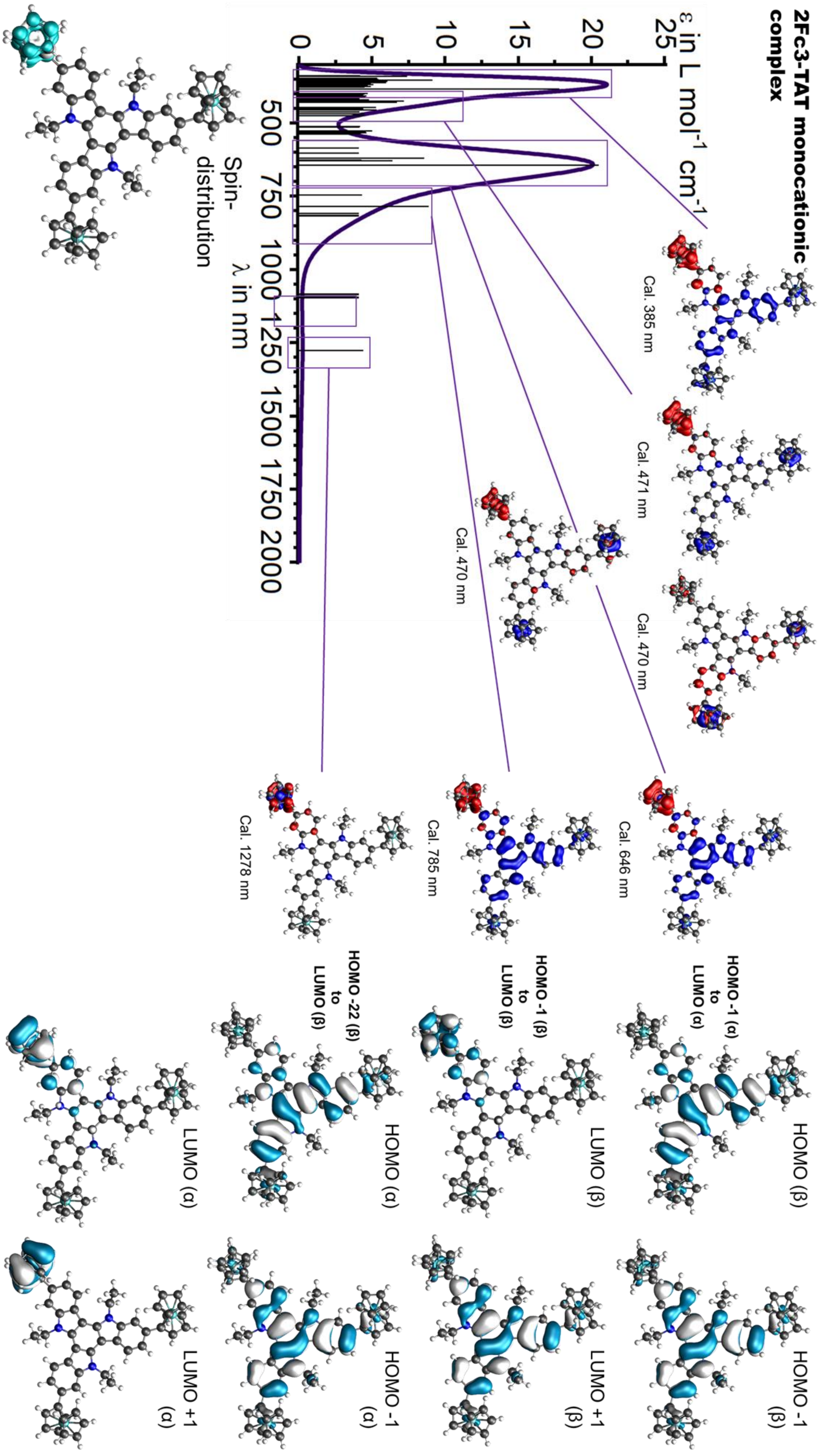

Figure S53. TD-DFT calculated UV/Vis/NIR-spectrum of one electron-oxidized **2-Fc$_3$-^{Et}TAT$^+$, 1$^+$**, with EDDMs for the individual transitions. Contributing MOs and band assignments are also provided.

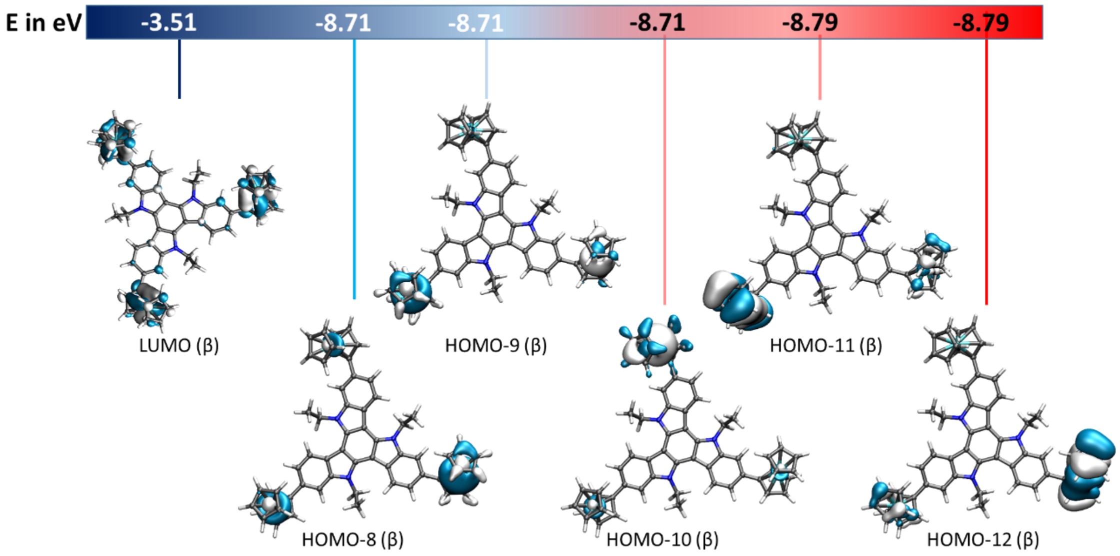

Figure S54. Contour diagrams of relevant MOs of **2-Fc$_3$-^{Et}TAT^{3+}** with their energies.

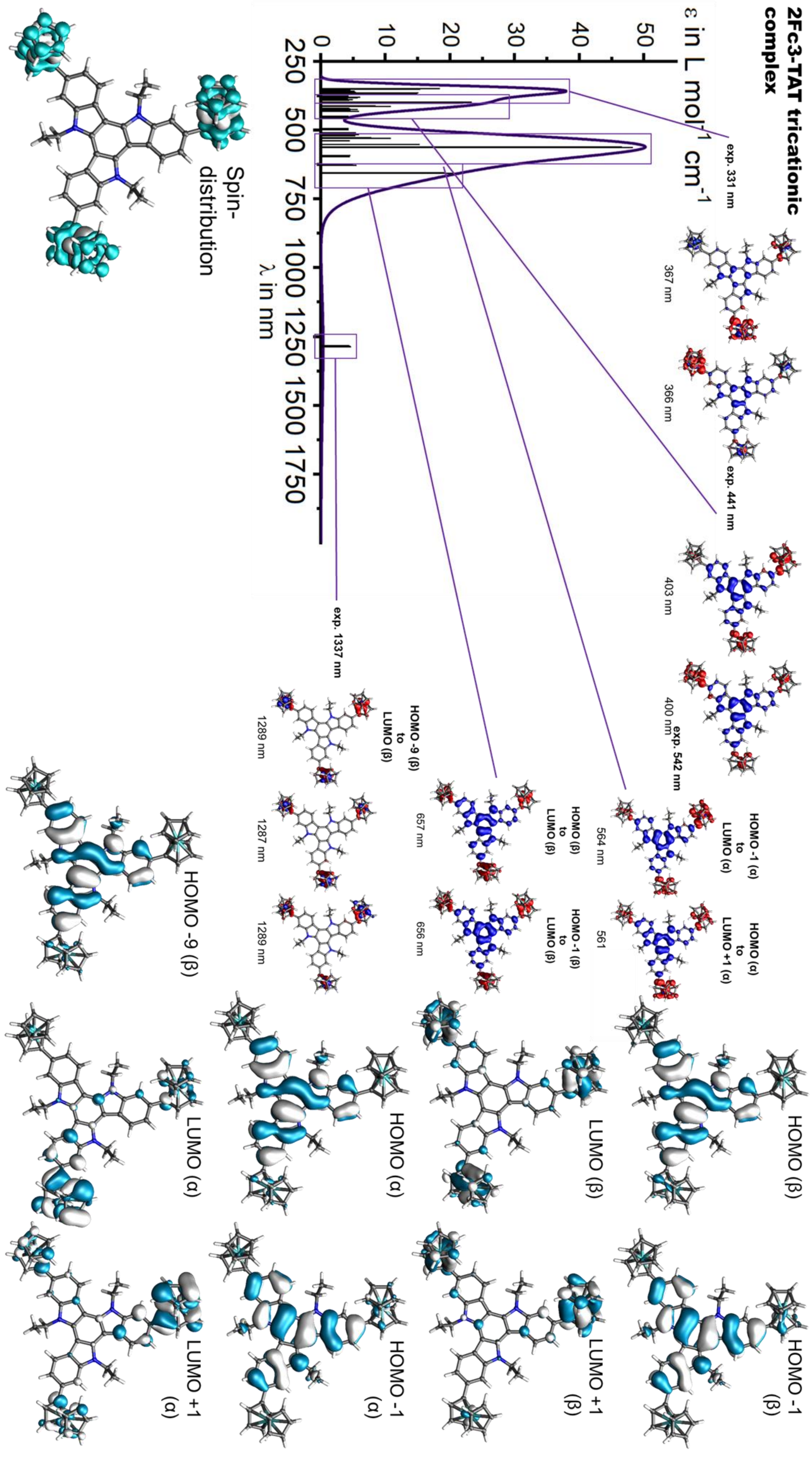

Figure S55. TD-DFT calculated UV/Vis/NIR-spectrum of **2-Fc₃-^{Et}TAT^{3+}** (quartet state) with EDDMs for the individual transitions. Contributing MOs and band assignments are also provided.

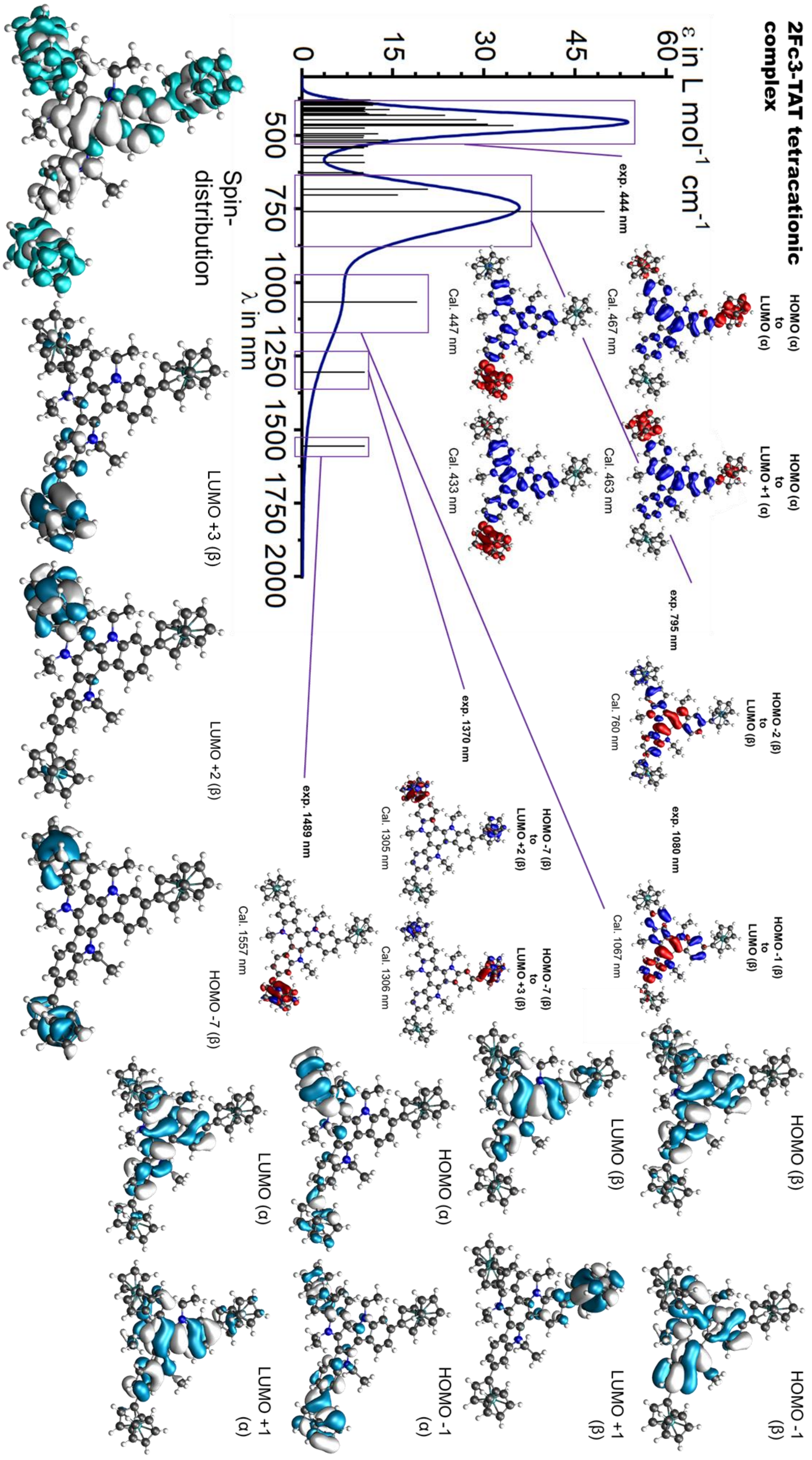

Figure S56. TD-DFT calculated UV/Vis/NIR-spectrum of **2-Fc$_3$-^{Et}TAT^{4+}** (quintet state) with EDDMs for the individual transitions. Contributing MOs and band assignments are also provided.

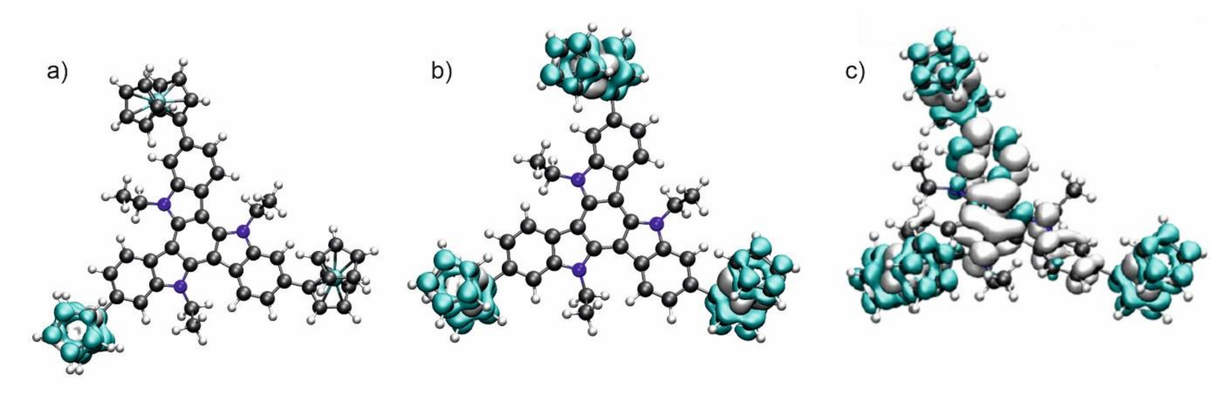

Figure S57. Calculated spin densities of a) **2-Fc$_3$-^{Et}TAT$^+$**, b) **2-Fc$_3$-^{Et}TAT^{3+}** (quartet state), and c) **2-Fc$_3$-^{Et}TAT^{4+}** (quintet state).

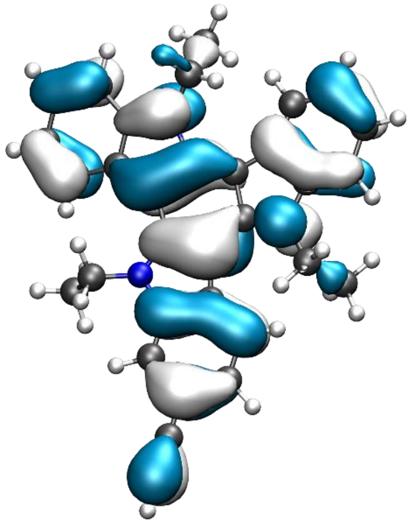

Figure S58. Contour diagram of the HOMO of **2-A$_1$-^{Et}TAT**.

STM switching

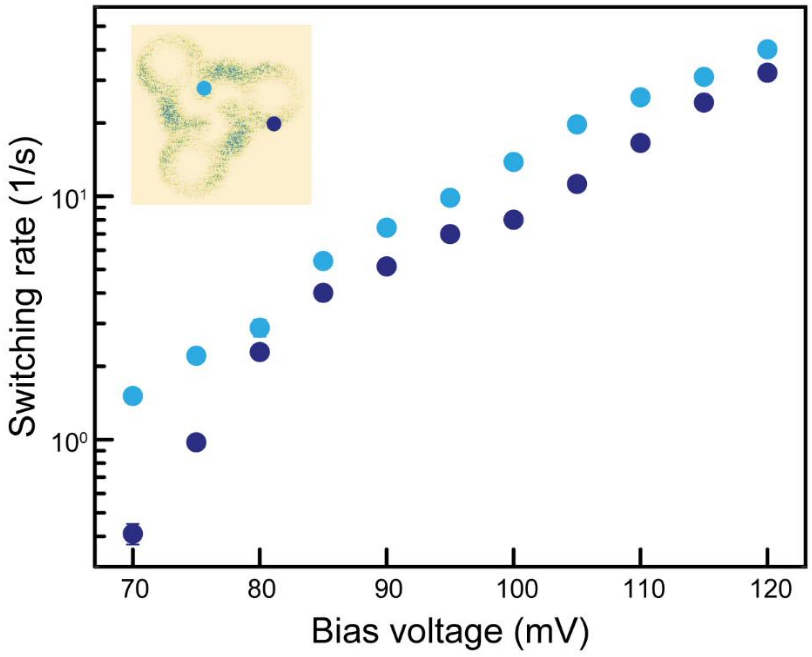

Figure S59. Bias voltage dependent switching rate (in s^{-1}) of **2-(Fc-A)$_3$-^{Et}TAT** (*R*) when the STM tip is positioned on the TAT core (blue) or a ferrocenyl protrusion (dark blue) at I_{set} = 100 pA and T = 5 K.

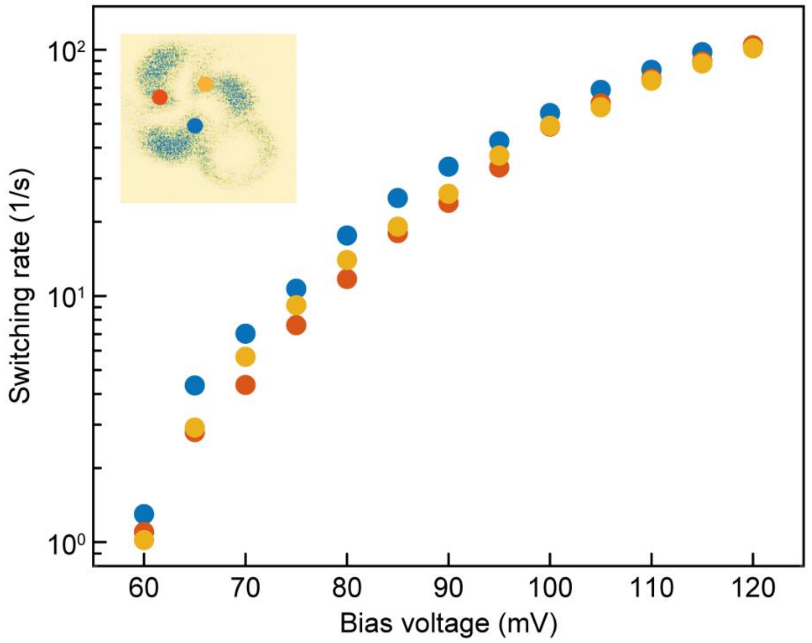

Figure S60. Bias voltage dependent switching rate (in s^{-1}) measured at the three noise clouds of **2-(Fc-A)$_1$-^{Et}TAT (*R*)** at I_{set} = 100 pA and T = 5.1 K.

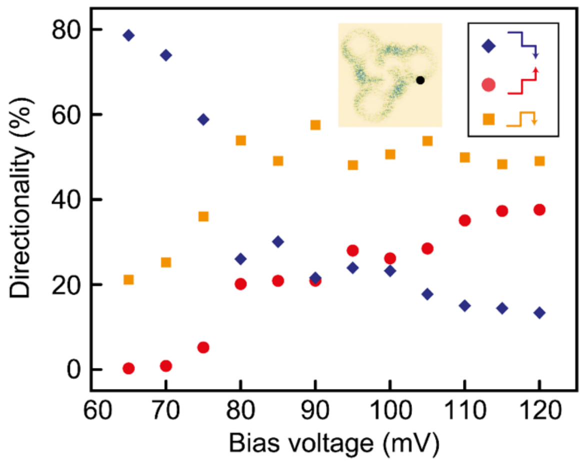

Figure S61. Switching directionality in percent against the applied bias voltage in mV of the **2-(Fc-A)$_3$-^{Et}TAT** (*R*) when the STM tip was positioned over one of the ferrocenyle units. with I_{set} = 100 pA and T = 5.0 K.

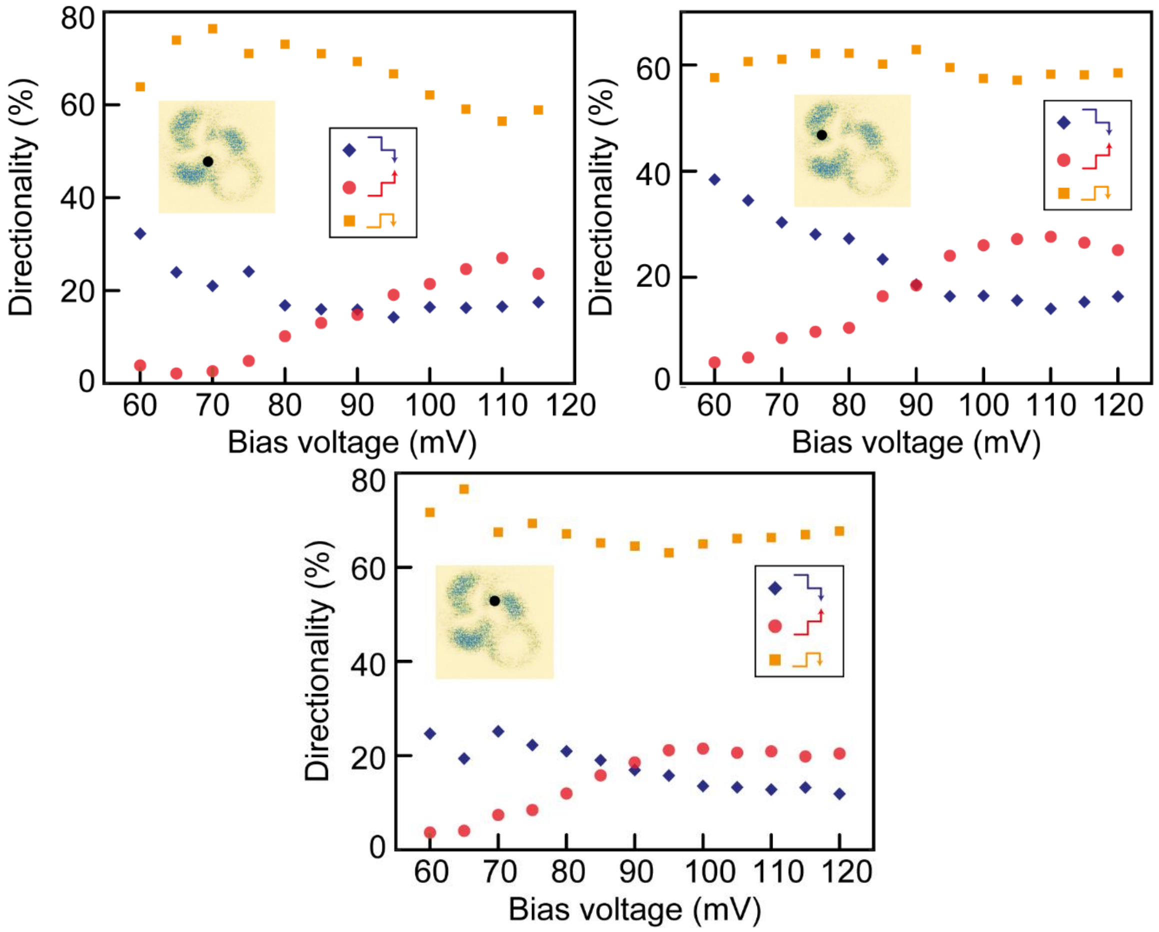

Figure S62. Switching directionality in percent of **2-(Fc-A)$_1$-^{Et}TAT** (***R***) measured at a position close to one of the three noise clouds near the TAT core (marked with a purple spot in the inset) against the applied bias voltage (in mV) with I_{set} = 100 pA and T = 5.1 K.

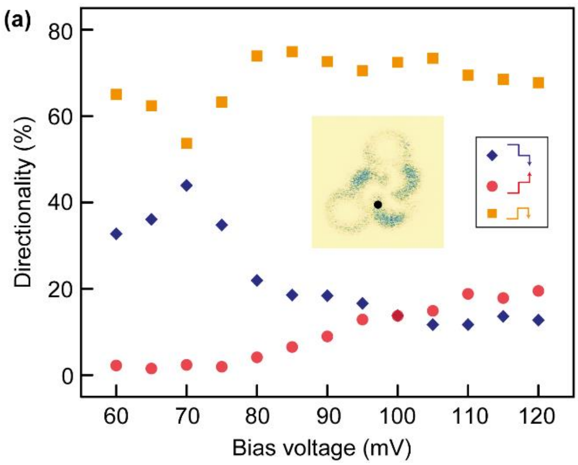

Figure S63. Switching directionality in percent of **2-(Fc-A)$_2$-^{Et}TAT** (*R*) measured at a position close to one of the three noise clouds near the TAT core (marked with a purple spot in the inset) against the applied bias voltage (in mV), with I_{set} = 100 pA and T = 5.1 K.

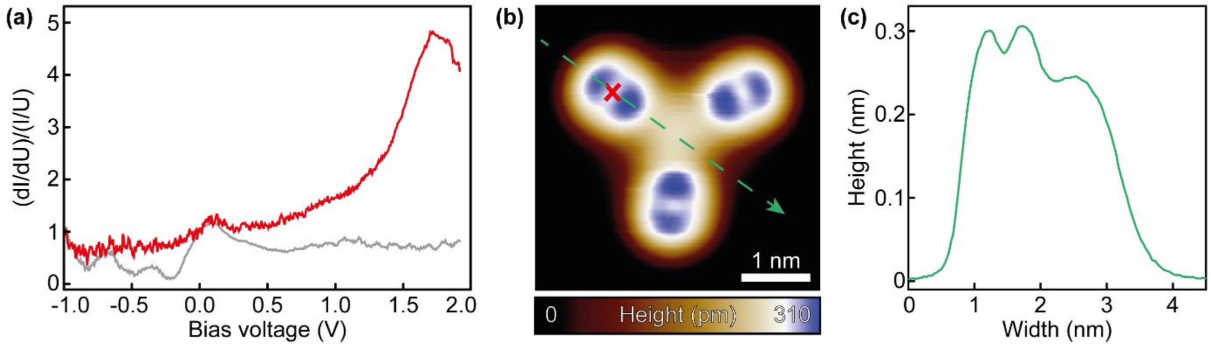

Figure S64. (a) Normalized d*I*/d*U* spectrum measured on a ferrocenyle unit of **2-(Fc-A)$_3$-^{Et}TAT** (red) with additional reference curve measured on Ag(111) in proximity to the molecule (grey). Scanning parameters: Iset = 100 pA, T = 4.8 K. (b) Topographic image of **2-(Fc-A)$_3$-^{Et}TAT** recorded at the bias voltage of 1.8 V corresponding to the LUMO resonance visible in (a). The blue donut-like features represent the ferrocenyl moieties. A red cross denotes the tip position for the d*I*/d*U* measurement. Scanning parameters: *U* = 1.8 V, I_{set} = 50 pA, T = 4.8 K. (c) Height profile measured along the green arrow shown in (b).

References

(1) Reger, D. L.; Wright, T. D.; Little, C. A.; Lamba, J. J.; Smith, M. D. Control of the stereochemical impact of the lone pair in lead(II) tris(pyrazolyl)methane complexes. Improved preparation of Na{B($_{3,5}$-$(CF_3)_2C_6H_3)_4$}. *Inorg. Chem.* **2001**, *40* (15), 3810–3814.
(2) Li, Y.; Josowicz, M.; Tolbert, L. M. Diferrocenyl molecular wires. The role of heteroatom linkers. *J. Am. Chem. Soc.* **2010**, *132* (30), 10374–10382.
(3) BASi Research Products. *DigiSim*; Bioanalytical Systems, Jena, 1994.
(4) Krejčik, M.; Daněk, M.; Hartl, F. Simple construction of an infrared optically transparent thin-layer electrochemical cell. *J. electroanal. chem. interfacial electrochem.* **1991**, *317* (1-2), 179–187.
(5) Frisch, M. J.; Trucks, G. W.; Schlegel, H. B.; Scuseria, G. E.; Robb, M. A.; Cheeseman, J. R.; Scalmani, G.; Barone, V.; Mennucci, B.; Petersson, G. A.*et al.* Inc.: Wallingford,CT, USA,2009.
(6) T. H. Dunning Jr. and P. J. Hay, In: H. F. Schaefer III, Ed. *Methods of Electronic Structure Theory Vol. 2, Plenum Press*, Vol 2, Berkeley, CA, USA, 1977.
(7) McLean, A. D.; Chandler, G. S. Contracted Gaussian basis sets for molecular calculations. I. Second row atoms, Z =11–18. *J. Chem. Phys.* **1980**, *72* (10), 5639–5648.
(8) Perdew, J. P.; Burke, K.; Ernzerhof, M. Generalized Gradient Approximation Made Simple. *Phys. Rev. Lett.* **1996**, *77* (18), 3865–3868.
(9) Cossi, M.; Rega, N.; Scalmani, G.; Barone, V. Energies, structures, and electronic properties of molecules in solution with the C-PCM solvation model. *J. Comput. Chem.* **2003**, *24* (6), 669–681.
(10) Paschke, F.; Erler, P.; Gragnaniello, L.; Dreiser, J.; Fonin, M. Electrospray Deposition and Magnetic Properties of Prototypical Molecular Magnets. *Quantum Mater Res* **2020**, 1:e200002.
(11) Ji, L.; Fang, Q.; Yuan, M.-S.; Liu, Z.-Q.; Shen, Y.-X.; Chen, H.-F. Switching high two-photon efficiency: from 3,8,13-substituted triindole derivatives to their 2,7,12-isomers. *Org. Lett.* **2010**, *12* (22), 5192–5195.
(12) Shmidt, M. S.; Perillo, I. A.; González, M.; Blanco, M. M. Reaction of isatin with alkylating agents with acidic methylenes. *Tetrahedron Lett.* **2012**, *53* (20), 2514–2517.
(13) Black, H. T.; Pelse, I.; Wolfe, R. M. W.; Reynolds, J. R. Halochromism and protonation-induced assembly of a benzogindolo2,3-bquinoxaline derivative. *ChemComm.* **2016**, *52* (87), 12877–12880.
(14) Luo, X.; Tran, D. T.; Kadlubowski, N. M.; Ho, C. H. Y.; Riley, P.; So, F.; Mei, J. Side-Chain Sequence Enabled Regioisomeric Acceptors for Conjugated Polymers. *Macromolecules* **2018**, *51* (21), 8486–8492.
(15) Roemer, M.; Nijhuis, C. A. Syntheses and purification of the versatile synthons iodoferrocene and 1,1'-diiodoferrocene. *Dalton Trans.* **2014**, *43* (31), 11815–11818.
(16) Erb, W.; Hurvois, J.-P.; Roisnel, T.; Dorcet, V. Ferroceneboronic Acid and Derivatives: Synthesis, Structure, Electronic Properties, and Reactivity in Directed C–H Bond Activation. *Organometallics* **2018**, *37* (21), 3780–3790.
